# New thiazole, thiophene and 2-pyridone compounds incorporating dimethylaniline moiety: synthesis, cytotoxicity, ADME and molecular docking studies

**DOI:** 10.1186/s13065-024-01136-z

**Published:** 2024-03-14

**Authors:** Heba M. Metwally, Norhan M. Younis, Ehab Abdel-Latif, Ali El-Rayyes

**Affiliations:** 1 Department of Chemistry, Faculty of Science, Mansoura University, Mansoura, 35516 Egypt; 2 Department of Chemistry, Faculty of Science, Northern Border University, 1321, Arar, Saudi Arabia

**Keywords:** Thiazole, Thiophene, 2-pyridone, Anticancer, MTT-assay, ADME, Docking

## Abstract

Various sets of thiazole, thiophene, and 2-pyridone ring structures containing a dimethylaniline component were synthesized. Substituted thiazoles **2**–**3** and thiophenes **5**–**7** were produced by reacting thiocarbamoyl compound **4** with α-halogenated reagents in different basic conditions. Also, a series of 2-pyridone derivatives **9a**–**f** substituted with dimethylaniline was synthesized through Michael addition of malononitrile to α,β-unsaturated nitrile derivatives **8a**–**f**. The synthesized products were structurally proven by spectroscopic methods such as IR, ^1^H NMR, ^13^C NMR, and MS data. Furthermore, the anti-cancer efficacy of the compounds was assessed using the MTT assay on two cell lines: hepatocellular carcinoma (**HepG-2**) and breast cancer (**MDA-MB-231**). The results showed the highest growth inhibition for derivatives **2**, **6**, **7**, and **9c**, which were further examined for their IC_50_ values. The IC_50_ for compound **2** showed equipotent activity (IC_50_ = 1.2 µM) against the HepG-2 cell line compared to Doxorubicin (IC_50_ = 1.1 µM). Compounds **2**, **6**, **7** and **9c** showed very good ADME assessments for further drug administration. Moreover, the PASS theoretical prediction for the compounds showed high antimitotic and antineoplastic activities for compounds **2**, **6**, **7**, and **9c**, as well as potent inhibition activity for the insulysin enzyme (IDE). Molecular docking stimulations were performed on CDK1/CyclinB1/CKS2 (PDB ID: 4y72) and BPTI (PDB ID: 2ra3). When docked into (PDB ID: 4y72), all of the tested compounds showed considerable inhibition, and the 2-pyridone derivative **9d** had the maximum binding affinity (− 8.1223 kcal/mol). While thiophene derivative **6** offered the maximum binding affinity (− 7.5094 kcal/mol) when docked into (PDB ID: 2ra3).

**Graphical Abstract:**

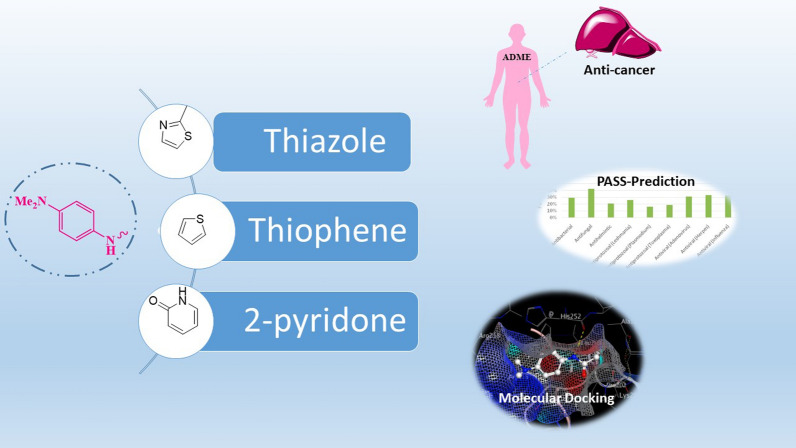

**Supplementary Information:**

The online version contains supplementary material available at 10.1186/s13065-024-01136-z.

## Introduction

**Fig. 1:**
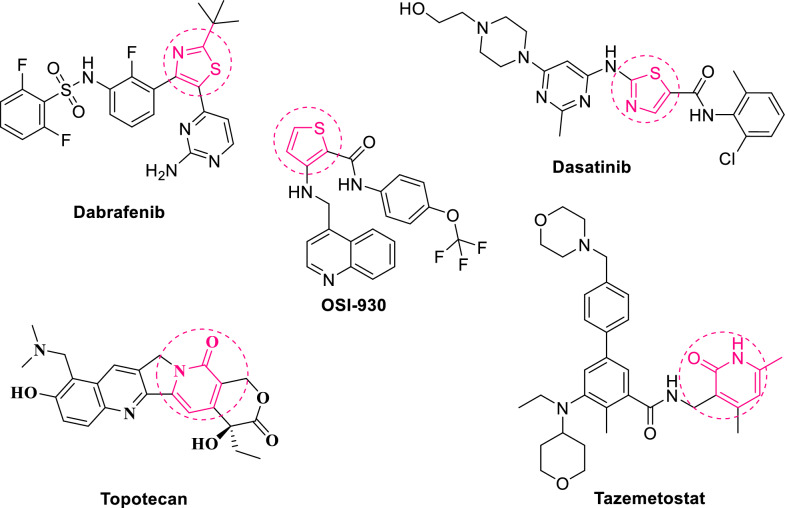
Anticancer marketed-drugs containing thiazole, thiophene and 2-pyridone

Human death leading cause in the world is attributed to cancer [1]. Breast cancer is the most common cancer in women and the main cause of death. In Egypt, breast cancer constitutes 33% of female cancer cases, and a high frequency rate counts for hepatocellular carcinoma [2]. Moreover, two main problems in searching for a new anti-tumor drug are serious side effects and the rapid development of drug resistance [3]. Several studies have been carried out using various sulfur and/or nitrogen-containing heterocyclic compounds, including thiophene, thiazole, and pyridine, directed towards different pathologies. These thiophene, thiazole, and pyridine-containing compounds show anticancer [4–8], anti-inflammatory [9–11], antibacterial [12–14], antioxidant [15], anti-oxidant [16], anti-fungal [17, 18], anti-coronavirus [19, 20] properties. Thiazoles were also found to act as anti-Alzheimer [21], anti-tubercular [22], and anti-diabetic [23]. While pyridone as well exhibits antimalarial [24], anti-hepatitis B [25], cardiotonic [26], and anti-fibrosis [27] properties. Some of these thiophene, thiazole, and pyridone-containing compounds have been transferred into clinical trials and cancer therapy, acting via multiple pathways [28]. Dabrafenib and Dasatinib are examples of thiazole-containing selective drugs with tyrosine kinase inhibitory activity (Fig. 1) [29, 30]. OSI-930 is a thiophene-containing orally specific inhibitor of Kit and kinase insert domain receptor tyrosine kinases (Fig. 1) [31]. Topotecan and Tazemetostat are 2-pyridone-containing cancer drugs that act as potent topoisomerase 1 inhibitors and selective EZH2 inhibitors, respectively (Fig. 1) [32]. The cyclin-dependent kinases (CDKs) are essential proteins that play an important role in cell-cycle control [33]. CDK1 inhibition has been found to effectively break cancer cell proliferation [34]. Some studies reveal thiazole-containing compounds as potent CDK inhibitors [35]. One of the most efficient synthetic strategies for thiophenes and thiazoles is the use of α-halogenated reagents to cyclize generous thiocarbamoyl derivatives. This method creates a varied substitution at all possible sites and an easy workup [36]. Cyanoacetanilide derivative cyclization with active methylene is one of the best methods for 2-pyridone synthesis [37]. The current work is centered on the synthesis of bioactive substituted thiazoles, thiophenes, and 2-pyridone molecules substituted with dimethylaniline. The biological evaluation included anticancer activity testing and ADME assessment for future medication delivery. Furthermore, theoretical predictions for the new compounds and molecular docking studies were performed to examine their anticancer inhibition activity.

## Results and discussion

### Chemistry

**Scheme 1:**
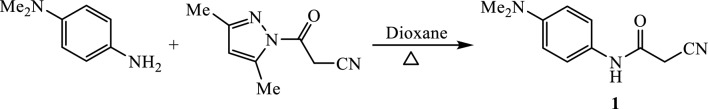
Preparation of 2-cyano-*N*-(4-(dimethylamino)phenyl)acetamide (**1**)

The synthetic strategy is based on the precursor 2-cyano-*N*-(4-(dimethylamino)phenyl)acetamide (**1**). It was synthesized in good yield through the well-known cyanoacetylation reaction of *N*,*N*-dimethylbenzene-1,4-diamine with 1-(cyanoacetyl)-3,5-dimethylpyrazole in dioxane (Scheme 1). The chemical structure and conformation of cyanoacetamide compound **1** were determined through spectral analysis. The IR absorption bands detected at 3280, 2221, and 1690 cm^−1^ correspond to the functional groups N–H, –C≡N, and –C=O, respectively. The ^1^H NMR spectrum indicated a singlet for six protons (dimethylamino group) at δ 2.83 ppm, a singlet for two protons (–CH_2_–) at δ 3.80 ppm, two doublet signals for the para-disubstituted benzene ring at δ 6.68 and 7.34 ppm, and singlet signal for the amidic proton at δ 9.98 ppm (N–H).

**Scheme 2:**
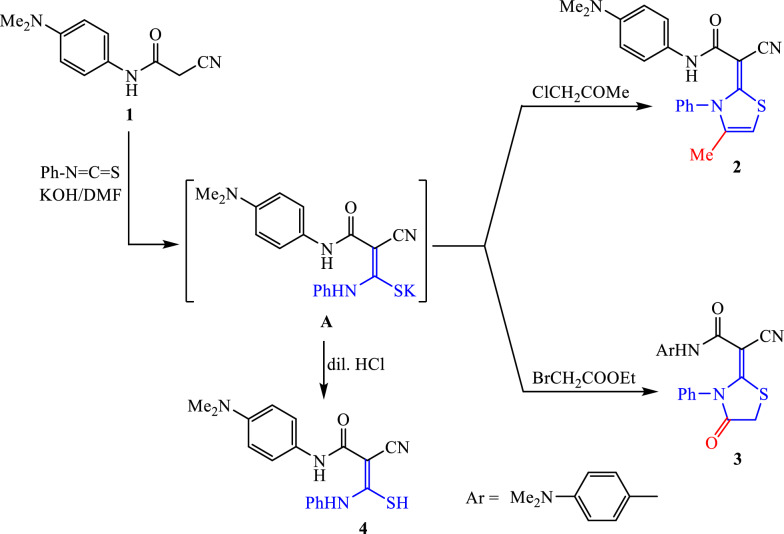
Synthesis of substituted thiazole derivatives **2** and **3**

The addition of 2-cyanoacetamide compound **1** to phenyl isothiocyanate was accomplished by stirring in DMF and potassium hydroxide to generate the non-isolated sulphide salt (intermediate A) (Scheme 2). This salt underwent in situ addition with chloroacetone to furnish the corresponding substituted thiazoline derivative **2**. The ^1^H NMR spectrum demonstrated the presence of two singlets at δ 1.28 and 6.87 ppm for the protons of the methyl group and thiazole-C5, respectively. Meanwhile, the thiazolidine-4-one compound **3** was obtained by in situ treatment of non-isolable sulfide salt (**A**) with ethyl bromoacetate. ^1^H NMR displayed four singlet signals at δ 2.82, 3.97, and 9.34 ppm, matching six protons (dimethylamino-NMe_2_), two protons (thiazolidine-CH_2_) and N–H functions. To get the notably rich thiocarbamoyl intermediate **4** (Scheme 2), the non-isolated sulphide salt was treated with water and then neutralized with diluted HCl. The structure was validated by spectral measurements (c.f. “Experimental”).

**Scheme 3:**
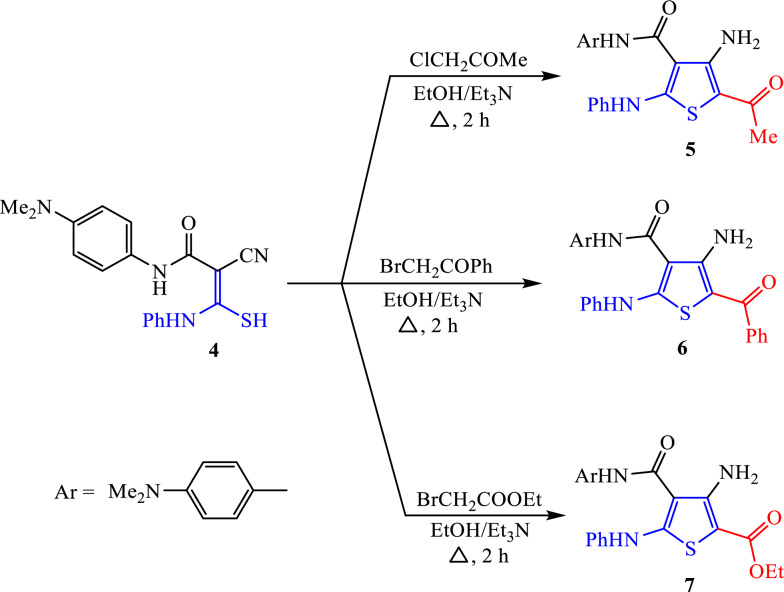
Synthesis of 4-amino-5-substititedthiophene derivatives **5**, **6** and **7**

The reactivity of thiocarbamoyl **4** towards different α-halo-ketones was investigated. Thus, nucleophilic substitution of thiocarbamoyl **4** on chloroacetone was achieved in refluxing ethanol and triethylamine. Following this, the nitrile group underwent intramolecular nucleophilic addition to produce 5-acetyl-4-aminothiophene derivative **5** (Scheme 3). Elemental studies and spectrum data confirmed the chemical structure of the newly created thiophene **5**. The formation of amino functionality was confirmed in both IR and ^1^H NMR spectra through strong absorptions at 3443 and 3282 cm^−1^ in the IR spectrum and a broad singlet signal at δ 7.47 ppm in the ^1^H NMR spectrum. Also, the ^1^H NMR spectrum indicated the lack of any signal related to the protons of the methylene group.

Similarly, the synthesis of 4-amino-5-benzoylthiophene derivative **6** and 4-amino-5-ethoxycarbonyl-thiophene derivative **7** was successful by treating thiocarbamoyl scaffold **4** with phenacyl bromide and ethyl bromoacetate in ethanol and triethylamine. The ^1^H NMR spectrum of derivative **6** indicates a characteristic singlet signal at δ 6.70 ppm and extra multiplet peaks in the region from δ 7.05 to 7.88 ppm for the protons of –NH_2_ group, which corresponds to phenyl rings. These new peaks appear next to the singlet signal at δ 2.84 ppm for six protons of two methyl groups (–N(CH_3_)_2_), two singlet signals at δ 9.62 and 9.82 ppm for two NH functions, and doublet signals at δ 7.04 to 7.88 ppm for the aromatic protons. In thiophene derivative **7**, strong absorptions at 3352, 3293, 1740, and 1663 cm^−1^ verified the presence of N–H, NH_2_ groups, and two carbonyl groups in the IR spectrum. While the ^1^H NMR spectrum indicated notable triplet and quartet signals at δ 1.34 and 4.28 ppm for protons of the ethoxy group (–OCH_2_CH_3_), besides singlet at δ 2.94 ppm for six protons (–N(CH_3_)_2_), and two singlet signals at δ 8.35 and 10.83 ppm corresponding to the protons of two (N–H) groups.

**Scheme 4:**
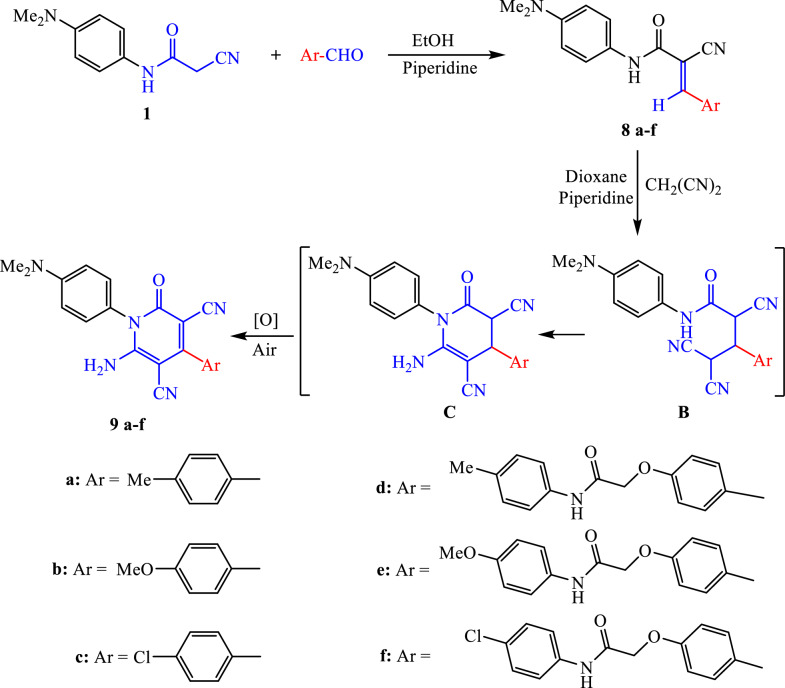
Synthesis of different *α*,*β-*unsaturated nitrile derivatives **8a**–**f** and their corresponding 2-pyridone derivatives **9a**–**f**

The synthetic strategy of 6-amino-4-aryl-1-(4-dimethylaminophenyl)-3,5-dicyano-2-oxo-2*H*-pyridine derivatives **9a**–**f** is explained in Scheme 4. Initially, the Knoevenagel condensation reaction between 2-cyanoacetanilide compound **1** and electronically different aromatic aldehydes in ethanol and piperidine offered α,β-unsaturated nitrile derivatives **8a**–**f**. Next, the cyclized targets 3,5-dicyanopyridone derivatives **9a**–**f** were achieved via Michael’s addition reaction of malononitrile to α,β-unsaturated nitrile compounds **8a**–**f** in refluxing dioxane and piperidine. The cyclization occurs through the formation of the suggested intermediates **B** and **C**. The nucleophilic addition of malononitrile to the beta-carbon of *α,β*-unsaturated functionality yields the intermediate Michael adduct **B**, which then undergoes intramolecular cyclization to give intermediate **C**. The last step is tetrahydropyridine intermediate **C** oxidation to furnish the final pyridine derivatives **9a**–**f**, whose structures were confirmed by spectroscopic examinations. We suggested that air (or the oxygen it contains) initiates the oxidation process.

Selected examples of formed structures are explored below using IR and ^1^H NMR spectra to prove the synthesized structures. For α,β-unsaturated nitrile **8d**, new absorption bands for carbonyl (C=O) and NH functions appear next to 2-cyanoacetanilide functions at 3407, 3338, 1696, and 1670 cm^−1^, respectively. Also distinguished singlet signals for the protons of methyl group (Ar–CH_3_), methylene group (CO–CH_2_–O), and olefinic proton (C=CH) were observed in the ^1^H NMR spectrum at 2.24, 4.81, and 8.15 ppm, respectively. In the same spectrum, twelve aromatic protons resonated as six doublet signals at 6.71–8.00 ppm, and a singlet signal was observed at 2.86 ppm corresponding to the –N(CH_3_)_2_. The protons of two NH functions appeared as two singlet signals at 10.00 and 10.08 ppm. For the 2-pyridone scaffold **9d**, the IR spectrum showed absorption at 3340, 3278, and 3183 cm^−1^ for the NH_2_ and N–H groups, 2213 cm^−1^ due to the nitrile group, and 1693 and 1659 cm^−1^ due to the carbonyl groups of the ester and amidic carbonyl groups, respectively. The ^1^H NMR spectrum recorded the singlet signals at 2.25, 2.97, and 4.78 ppm for the protons of the methyl and methylene groups (Ar–CH_3_, –N(CH_3_)_2_ and CO–CH_2_–O, respectively). Twelve aromatic protons appeared as doublet and multiplet signals at 6.83–7.50 ppm, while the N–H proton resonated at 10.07 ppm.

### Anticancer activity

**Fig. 2:**
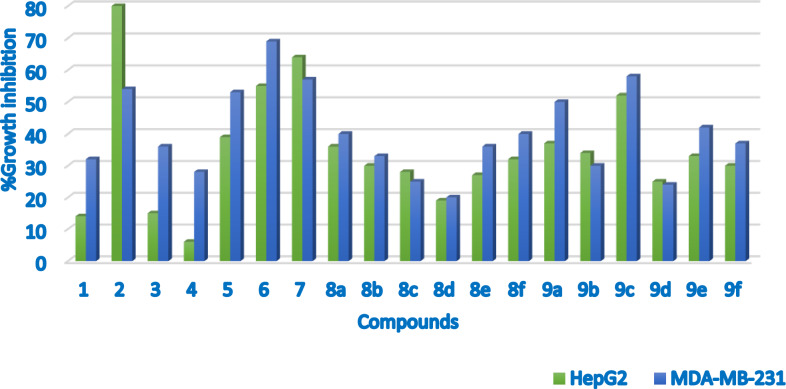
Cytotoxic activity against all compounds **1**–**9**(**a**–**f**) against HepG-2 and MDA-MB-231 cell lines

The cytotoxic effect of all compounds **1**–**9** on hepatocellular carcinoma (HepG-2) and breast cancer (MDA-MB-231) was examined using the MTT technique. The outcomes are presented in (Additional file 1: Table S4), while graphic plots of the growth inhibition curve against the compounds enable us to compare the inhibition activity for the tested compounds (Fig. 2). The results revealed that the tested drugs suppress cancer cells to varying degrees. At a 25 µM concentration after 48 h, compounds **2**, **6**, **7**, and **9c** had the most cytotoxic effect against the HepG-2 and the MDA-MB-231 cell lines. Compounds **2**, **6**, **7**, and **9c** were selected for the IC_50_ experiment using doxorubicin as a reference (Table 1, Additional file 1: Figs. S41–S43). The IC_50_ evaluation against the HepG-2 cell line showed that compound **2** containing the thiazole moiety acts as a potent anticancer with an IC_50_ value of 1.2 µM, which is equivalent to the doxorubicin IC_50_ value of 1.1 µM.

**Table 1:** IC_50_ (µM) against HepG2, and MDA-MB-231

	**2**	**6**	**7**	**9c**	**Doxorubicin**
HepG2 (A2780CP)	1.2	28.7	6.4	33.08	1.1
MDA-MB-231 (A2780)	26.8	14.1	21.9	21.73	3.16

Compounds **6** and **7** showed IC_50_ values of 28.7 and 6.4 µM, respectively. The highest inhibitory activity was observed for compound **9c**, which contains a pyridine moiety with an IC_50_ value of 33.08 µM.

IC_50_ evaluation against the MDA-MB-231 cell line shows the lowest IC_50_ values for compounds **6**, **7**, and **9c** (14.1, 21.9, and 21.73 µM), respectively. While the highest IC_50_ value (26.8 µM) is for compound **2**. The derivative **2** showed superiority as a possible anticancer agent due to its low toxicity to normal cells (PBMC human peripheral blood mononuclear cells). (IC_50_ < 30 µM) compared to Doxorubicin IC_50_ (2 µM).

### Structure activity relationship (SAR) studies

**Fig. 3:**
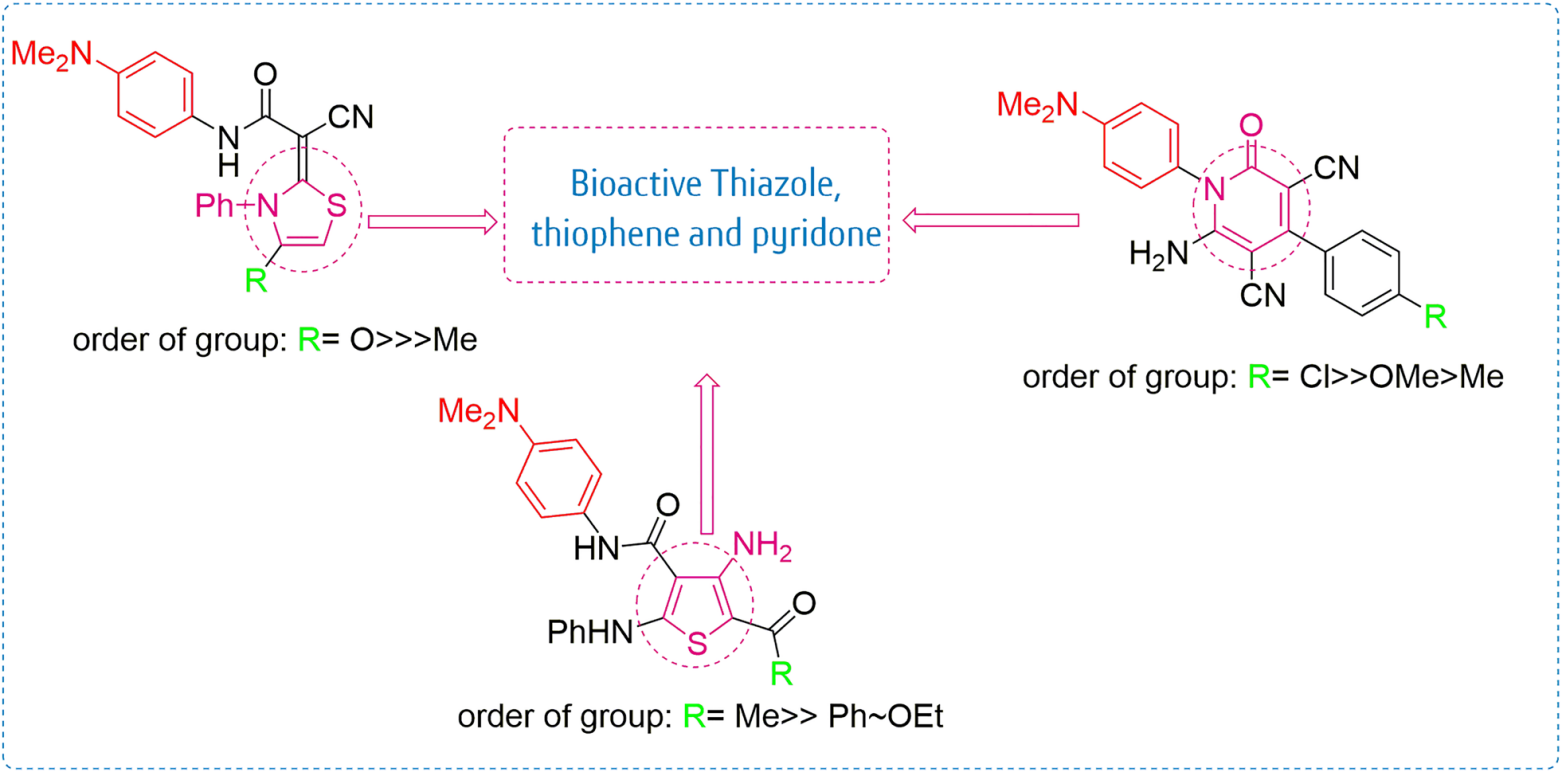
Structure–activity relationship (SAR) of thiazole, thiophene, pyrindone derivatives

By analyzing the outcomes, we uncovered important structure–activity relationship facts (Fig. 3). Thiazole derivative **2** with a substituted methyl group at position 4 of the thiazole ring markedly recorded the strongest inhibitory effect against tested HepG2 and MDA-MB-231 cell lines (80 and 54%) with IC_50_ values of 1.2 and 26.8 µM, respectively. The effect of changing the methyl group at position 4 of the thiazole ring for compound **2** with the carbonyl group for compound **3** caused a decrease in inhibitory effect against tested cell lines (15 and 36%). It is clear that the cyanoacetamide derivative **1** and mercaptocrylamide derivative **4** displayed the weakest activity (14 and 6%) against the liver cell line and (32 and 28%) against the breast cell line. Also, the absence of the thiazole moiety in derivatives **1** and **4** may lead to the loss of inhibitory activity. Among the 3-amino thiophene derivatives **5**–**7**, it was found that the presence of benzoyl and ester groups at position 2 in compounds **6** and **7** led to an increase in inhibition activity (55 and 64%) with IC_50_ values (28.7 and 14.1 µM) against the liver cell lines and (69 and 57%) with IC_50_ values (6.4 and 21.9 µM) against breast cell lines. The replacement of these groups with an acyl group at position 2 of the thiophene ring led to a decrease in the inhibitory activity. Likewise, acrylamide derivatives **8a**–**f** showed moderate to low inhibition potency against cell lines 19–40%. Moreover, pyridine derivatives **9a**–**f** showed moderate to high inhibition potency against cell lines 24–58%. Pyridine derivative **9c** with a substituted Cl group at position 4 of the phenyl ring exhibited the strongest activity among the pyridine series (52 and 58%) against liver and breast cell lines with IC_50_ values of 33.08 and 21.73 µM, respectively. The effect of exchanging the chlorine atom for compounds **9c** with methyl and methoxy groups for compounds **9a** and **9b** resulted in a decrease in inhibition growth values (37 and 34%) and (50 and 30%), respectively, towards the examined cell lines. Also, the addition of a linker (–OCH_2_CONHAr–) in pyridine derivatives **9d**–**f** resulted in reduced activity compared to their direct 4-substituted phenyl ring pyridine counterparts **9a**–**c**.

### Computational analysis

#### Prediction of in-silico ADME and oral bioavailability

During the drug discovery work, a lot of attention goes to the pharmacodynamics of the newly synthesized small molecules. While the promotion of a drug candidate focuses on the pharmacokinetics behavior of such molecules [38]. In-silico ADME screening was applied to the synthesized compounds to calculate the putative absorption, distribution, metabolism, and excretion properties [39]. Predictions for compounds **1**–**9** were listed in (Table 2, Additional file 1: Table S1) and processed using the SwissADME webtool provided through the Swiss Institute of Bioinformatics (SIB) (https://www.swissadme.ch) and AdmetSAR-2 online software predictors.

**Table 2:** ADME and drug-likeness profiles of the new compounds

Molecule	Rotatable bonds	H-bond acceptors	H-bond donors	MR	TPSA	MLOGP	ESOL solubility (mol/l)	GI absorption	BBB permeant	Pgp substrate	Lipinski violations	PAINS alerts
**1**	4	2	1	59.52	56.13	0.89	9.14E−03	High	Yes	No	0	1
**2**	5	2	1	110.76	89.3	1.92	7.26E−06	High	No	No	0	1
**3**	5	3	1	111	101.74	1.4	4.85E−05	High	No	No	0	1
**4**	6	2	2	100.61	106.96	1.82	2.07E−05	High	No	No	0	1
**5**	7	2	3	116.88	115.7	1.99	4.49E−06	High	No	No	0	1
**6**	8	2	3	136.56	115.7	2.83	1.69E−07	Low	No	No	0	1
**7**	9	3	3	122.77	124.93	2.22	1.97E−06	Low	No	No	0	1
**8a**	5	2	1	94.1	56.13	2.61	5.67E−05	High	Yes	No	0	1
**8b**	6	3	1	95.62	65.36	2.05	9.74E−05	High	Yes	No	0	1
**8c**	5	2	1	94.14	56.13	2.88	2.91E−05	High	Yes	No	0	1
**8d**	10	4	2	134.42	94.46	2.53	2.80E−06	High	No	No	0	1
**8e**	11	5	2	135.95	103.69	2.01	4.69E−06	High	No	No	0	1
**8f**	10	4	2	134.47	94.46	2.8	1.42E−06	High	No	No	0	1
**9a**	3	3	1	110.48	98.84	2.11	4.61E−05	High	No	No	0	1
**9b**	4	4	1	112.01	108.07	1.58	7.93E−05	High	No	No	0	1
**9c**	3	3	1	110.53	98.84	2.37	2.36E−05	High	No	No	0	1
**9d**	8	5	2	150.81	137.17	2.03	3.48E−06	Low	No	No	1	1
**9e**	9	6	2	152.33	146.4	1.53	5.83E−06	Low	No	No	1	1
**9f**	8	5	2	150.85	137.17	2.3	1.76E−06	Low	No	No	1	1

Generally, all the investigated compounds **1**–**9** possess acceptable physicochemical and pharmacokinetic properties with zero violation of Lipinski’s rule of five, except for derivatives **9d**, **9e**, and **9f**. Regarding the parameters associated with Lipinski’s rule of five, all compounds have 4 to 10 rotatable bonds, 2 to 6 hydrogen bond acceptors, 1 to 3 hydrogen bond donors, molar refractivity (MR) 59.52 to 150.85, topological polar surface area (TPSA) between 56.13 and 137.17, and a predicted logPo/w in the range of 0.89 to 2.88 [40]. Moreover, they displayed moderate water solubility and high GIT absorption, except for derivatives **6**, **7**, **9d**, **9e**, **9f** and one PAINS. These results support the oral bioavailability of the compounds [41]. Consequently, any compound showing two or more violations must be excluded from further study.

**Fig. 4:**
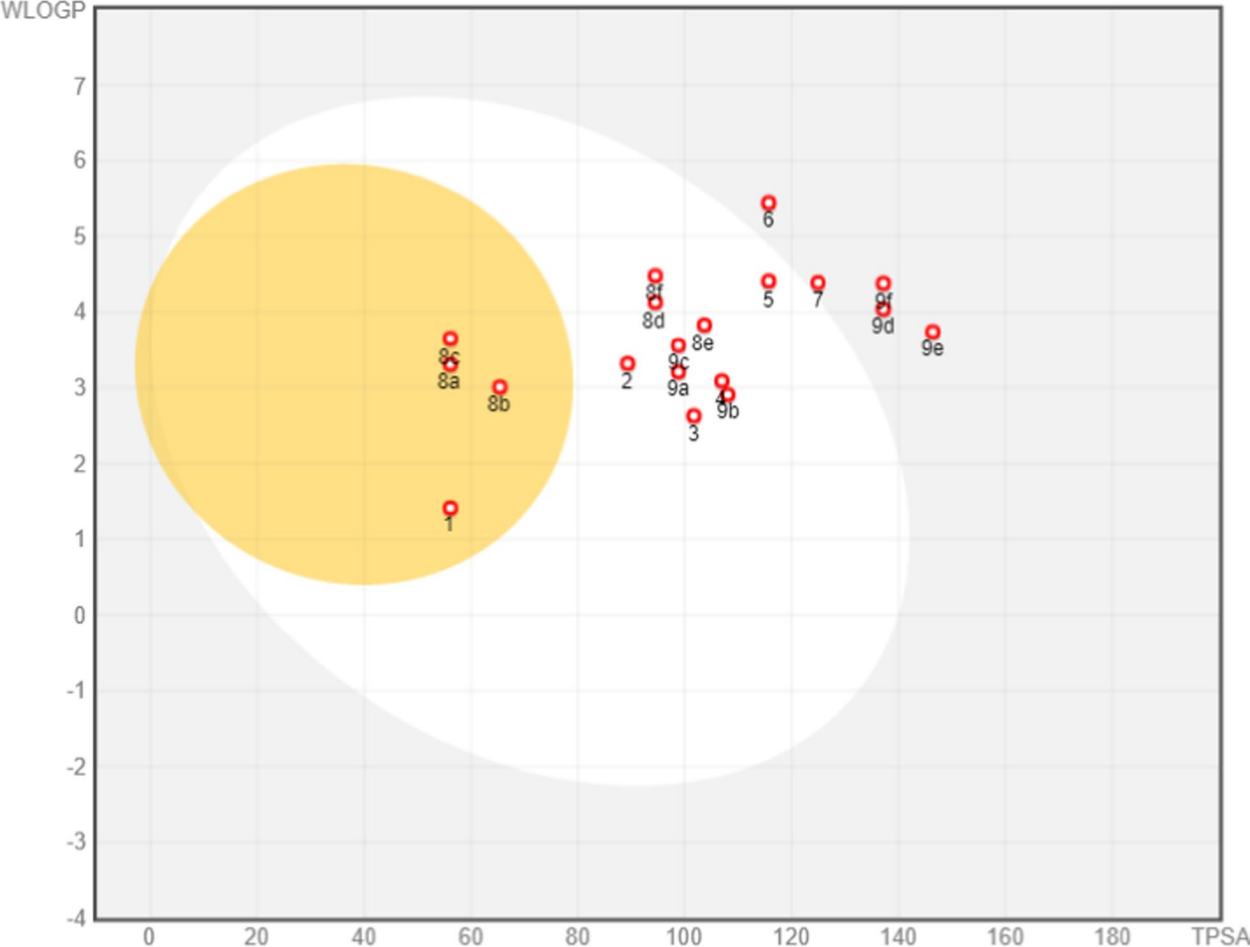
Swiss ADME boiled-egg plot for compounds **1**–**9**(**a**–**d**)

The correlation between the WLOGP and TPSA (topological polar surface area) for the newly synthesized compounds is presented in the BOILED-EGG graph [42]. Figure 4 categorizes the compounds in regions, most of them located in the region of human intestinal absorption (HIA), except **5**, **6**, **7**, **9d**, **9e**, and **9f**. The HIA region has no BBB permeability except for **1**, **8a**–**8c**, located in the yolk of the BOILED-EGG, which is expected to be BBB permeant. These results reflect good oral absorption for our compounds, along with poor penetration of the BBB, and hence no possible side effects on the CNS. Much more, a promising character for the compounds as they do not support a substrate for P-glycoprotein (Pgp), which is considered a drug efflux transporter [43]. In other words, all compounds can exist in the target cancer cells, simulating their cytotoxic effect. To conclude, the most active derivatives **2**, **6**, **7**, and **9c** fulfil the ADME profiles required for good distribution in the body and oral bioavailability, avoiding cancer cell resistance mediated by Pgp.

#### Prediction of activity spectra using PASS online predictor

With the aid of Prediction of Activity Spectra for Substances (PASS) online software, we were able to assess the putative anticancer activity, which is presented in (Table 3, Additional file 1: Table S2) The results of the PASS prediction are based on the available biological activity of compound fragments in the database through the correlation between Pa and Pi (probability to be active or inactive) [44]. The results displayed high antimitotic and antineoplastic activities for compounds **2**, **6**, **7** and **9c** (Table 3) in addition to other activities (Additional file 1: Table S2). Interestingly, almost all compounds displayed potent inhibition activity for the insulysin enzyme, also called the insulin-degrading enzyme (IDE).

**Table 3:** A mitotic inhibitor, anticancer activity assessment using PASS online software

Compound no.	Antimitotic		Antineoplastic (non-small cell lung cancer)		Insulysin inhibitor	
	Pa	Pi	Pa	Pi	Pa	Pi
**1**	NA	NA	0.225	0.054	0.683	0.011
**2**	0.595	0.005	0.203	0.076	0.680	0.011
**3**	0.392	0.014	NA	NA	0.675	0.012
**4**	NA	NA	NA	NA	0.751	0.004
**5**	0.216	0.048	NA	NA	0.427	0.076
**6**	0.454	0.010	0.153	0.153	0.428	0.076
**7**	0.195	0.060	0.175	0.114	0.397	0.089
**8a**	NA	NA	0.197	0.083	0.874	0.003
**8b**	0.145	0.092	0.241	0.043	0.832	0.003
**8c**	NA	NA	0.170	0.122	0.820	0.003
**8d**	NA	NA	0.160	0.140	0.829	0.003
**8e**	NA	NA	0.200	0.079	0.805	0.003
**8f**	NA	NA	NA	NA	0.788	0.004
**9a**	NA	NA	NA	NA	0.292	0.145
**9b**	NA	NA	0.298	0.024	0.239	0.201
**9c**	NA	NA	0.234	0.048	NA	NA
**9d**	NA	NA	0.207	0.071	0.263	0.173
**9e**	NA	NA	0.238	0.045	0.226	0.219
**9f**	NA	NA	0.193	0.088	NA	NA

### Molecular docking

**Fig. 5.**
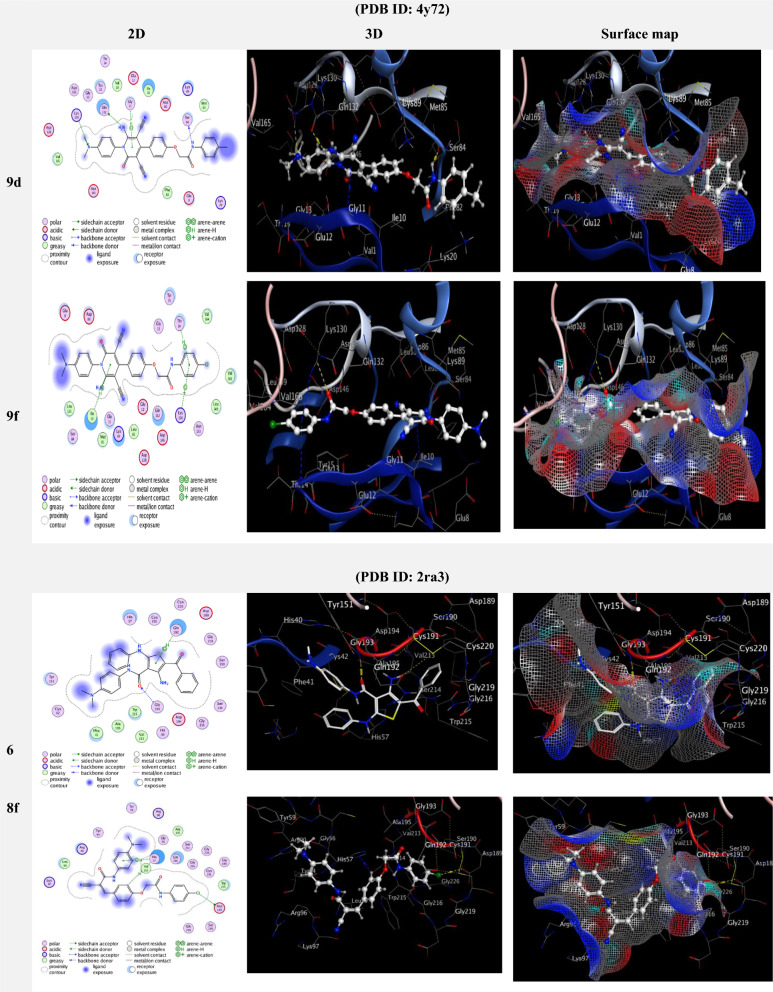
2D, 3D and surface map interaction between drugs **1**–**9**(**a**–**f**) and target proteins (4y72 and 2ra3)

In this study, the biological targets in the studied cancer cell lines were predicted in order to define the mechanism of anticancer activities for all synthesized derivatives. The interaction of the derivatives with two different targets was inspected through molecular docking studies in order to predict their binding sites and modes on enzymes. The molecular docking study was performed using MOE “v10.2015.10 software”. The choice of targets was based on the interaction of ATP-competitive inhibitors complexed with the cyclin-dependent kinase human CDK1/CyclinB1/CKS2 (PDB ID: 4y72) [45, 46]. Also, the PASS-Online software predicts inhibition activity for the insulysin enzyme, and based on that, different related PDB codes were tested. The best interaction was found to be with Human cationic trypsin complexed with bovine pancreatic trypsin inhibitor (BPTI) (PDB ID: 2ra3). The crystallographic coordinates of the proteins were downloaded from the Protein Data Bank [47]. Markedly, all of the compounds showed significant inhibition activities against 4y72 and were higher than previously prepared compounds (Table 4, Additional file 1: Table S3) [45]. A docking study of compounds **1**–**9(a**–**d**) showed that they can fit well in the ATP binding sites of CDK1 and BPTI (Additional file 1: Figs. S1–S40). The highest binding affinities for the prepared compounds with the targeted proteins 2ra3 and 4y72 are listed in (Table 4), while the binding affinities for the rest of the compounds are listed in (Additional file 1: Table S3). The compound **5** has a docking score of − 7.9160 kcal/mol. It forms strong hydrogen bonds with Asp 86. It also exhibits interactions with Gly 193, Lys 60, and Phe 41 through H-donor, H-acceptor, and π–H interactions, respectively (Fig. 5). Compound **8f** has a docking score of − 7.9504 kcal/mol, which is slightly more favorable than compound **5**. It forms potential strong binding interactions through hydrogen bonds with Lys 130 and Glu 12. Additionally, it shows interactions with Ile 10 through π–H, and H-acceptor interactions with benzene rings. Compound **9d** exhibits the highest docking score among the three compounds, with a score of − 8.1223 kcal/mol, indicating strong binding affinity. It forms hydrogen bonds with Gln 132, Ser 84, and Lys 130 with various functional groups of the compound, including *N*-Amino, *N*-Amide, and *N*-dimethylamino groups. Gly 11 also interacts with compound **9d** through a π–H interaction. The several hydrogen bonds and π–H interaction contribute to the strong binding affinity of compound **9d**. Compound **9f** has a docking score of − 8.0945 kcal/mol, which is quite favorable. It forms with Ile 10, Thr 14, and Lys 130 π–H interactions. Compound **9f** primarily interacts with the protein through its benzene ring, and these π–H interactions contribute to its binding affinity. To summarize, the docking results suggest that four compounds (**5**, **8f**, **9d**, and **9f**) strongly bind to the target protein due to various types of interactions. Compound **9d** stands out with the highest docking score, indicating the strongest predicted binding affinity. These findings provide valuable insights into the potential of these compounds as drug candidates that could target a specific protein of interest.

**Table 4:** Interaction between drugs **1**–**9**(**a**–**f**) and target proteins (4y72 and 2ra3) and their docking scores

Cpd no.	(PDB ID: 4y72)						(PDB ID: 2ra3)					
	S (energy binding score) (Kcal/mol)	RMSD	Distance (A)	Binding interaction			S (energy binding score) (Kcal/mol)	RMSD	Distance (A)	Binding interaction		
				Ligand	Receptor	Type				Ligand	Receptor	Type
**5**	− 7.9160	1.0834	3.03	*N*-Amino	Asp 86	H-donor	− 6.8706	0.9802	2.89	*O*-Amide	Gly 193	H-acceptor
									2.99	*O*-Carbonyl	Lys 60	H-acceptor
									4.64	Benzene ring	Phe 41	π–H
**8f**	− 7.9504	1.3730	3.51	*N*-Nitrile	Lys 130	H-acceptor	− 7.2367	1.3140	3.57	Cl-atom	Asp 189	H-donor
			3.88	Benzene ring	Ile 10	π–H			4.06	C-Benzene ring	His 57	H–π
			3.95	Benzene ring	Glu 12	π–H			3.95	Benzene ring	His 57	π–H
**9d**	− 8.1223	1.5557	3.17	*N*-Amino	Gln 132	H-donor	− 6.1324	1.2871	3.05	*N*-Nitrile	Asn 34	H-acceptor
			2.94	*N*-Amide	Ser 84	H-donor			3.68	Benzene ring	Gly 38	π–H
			3.38	*N*-Dimethylamino Benzene ring	Lys 130	H-acceptor						
			4.16		Gly 11	π–H						
**9f**	− 8.0945	1.4239	4.06	Benzene ring 6-benzene ring	Ile 10	π–H	− 5.9004	0.9566	3.07	*N*-Nitrile	Trp 237	H-acceptor
			4.45	Benzene ring	Thr 14	π–H			3.64	Benzene ring	Pro 92	π–H
			4.02		Lys 130	π–cation						
**Dox**	− 8.8931	1.4144	2.72	*O-*Hydroxy	Asn 133	H-donor	− 6.2060	1.4534	3.00	*N*-Amino	Arg 96	H-donor
									3.67	Benzene ring	His 57	π–H
									4.53	Quninone ring	Gln 192	π–H

## Conclusion

The study aimed to synthesize thiazole, thiophene, and 2-pyridone incorporating a dimethylaniline moiety and investigate their potential for biological activity. A library of compounds **1**–**9(a**–**f)** with varying structures to assess their potential as drug candidates. The cyclization reaction strategy for the thiocarbamoyl derivative using α-halogenated reagents in different reaction conditions serves to modify the chemical structures (thiazoles and thiophenes). Also, the Michael addition of malononitrile to α,β-unsaturated nitrile derivatives introduces functional groups into 2-pyridone derivatives. Anti-cancer activity evaluation on two different cancer cell lines (hepatocellular carcinoma and breast cancer) indicates potential anti-cancer properties. Compound **2** has equipotent activity for the Doxorubicin IC_50_ value (1.2 µM), while compounds **6**, **7**, and **9c** had the highest cytotoxic effect. Compounds **2**, **6**, **7**, and **9c** also have favorable ADME profiles. Compounds **2**, **6**, **7**, and **9c** are identified as particularly promising anticancer agents based on PASS predictions. Molecular docking stimulations with specific biological targets (CDK1/CyclinB1/CKS2 and BPTI) demonstrated that the synthesized compounds exhibited strong binding affinities with the protein residues. Meanwhile, 2-pyridone derivatives **9d** recorded an eminent docking score (-8.1223 kcal/mol) with the protein 4y72 and the thiophene **6** provided a great energy score (− 7.5094 kcal/mol) with 2ra3.

## Experimental

Experimental general remarks: melting points were determined with Gallenkamp melting point apparatus and are uncorrected. The infrared (IR) spectra were recorded on Thermo Scientific Nicolet iS10 FTIR. ^1^H NMR and ^13^C NMR spectra were recorded DMSO-*d*_6_ as a solvent using JEOL’s spectrometer at 500 MHz using tetramethylsilane (TMS) as internal standard. Chemical shifts are expressed in δ, ppm. ^1^H NMR data are reported in order: multiplicity (br, broad; s, singlet; d, doublet; t, triplet; dd, doublet of doublet; m, multiplet), approximate coupling constant in Hertz, number of protons and type of protons. The purity of the compounds was checked by ^1^H NMR and thin layer chromatography (TLC) on silica gel plates using a mixture of dichloromethane and methanol or petroleum ether and ethyl acetate as eluent. UV lamp was used as a visualizing agent. Mass analyses and elemental analyses were recorded on Thermo DSQ II spectrometer at Faculty of Science, Alazhar University. The ^13^C NMR spectra of compounds **9b**, **9c**, **9d**, **9e**, and **9d** are not recorded due to insufficient solubility in most of NMR solvents.

### Preparation of 2-cyano-*N-*(4-(dimethylamino)phenyl)acetamide (1)

A suspension of *N*,*N*-dimethylbenzene-1,4-diamine (0.04 mol, 4.32 g) and 1-cyanoacetyl-3,5-dimethylpyrazole (0.04 mol, 6.52 g) was refluxed in dioxane for 6 h. The precipitate that obtained was collected and dried to produce cyanoacetamide compound **1**.

Pale blue crystal; yield (65%); m.p. 189–191 °C. IR (ν/cm^−1^): 3280 (N–H), 2221 (C≡N), 1690 (C=O). ^1^H NMR (δ/ppm): 2.83 (s, 6H, –N(Me)_2_), 3.80 (s, 2H, CH_2_), 6.68 (d, *J* = 8.50 Hz, 2H, Ar–H), 7.34 (d, *J* = 8.50 Hz, 2H, Ar–H), 9.98 (s, 1H, NH). ^13^C NMR (δ/ppm): 30.94, 40.25, 40.61, 112.53 (2C), 116.87, 122.44 (2C), 128.76, 147.26, 165.38. Analysis Calcd. for C_11_H_13_N_3_O (203.11): C, 65.01; H, 6.45; N, 20.68%; found: C, 64.94; H, 6.40; N, 20.61%.

### Preparation of 2-cyano-*N*-(4-(dimethylamino)phenyl)-2-(4-methyl-3-phenylthiazol-2(3*H*)-ylidene)acetamide (2)

Stirring of cyanoacetamide compound **1** (0.005 mol, 1.02 g) with phenyl isothiocyanate (0.005 mol, 0.61 ml) in 20 ml DMF containing KOH (0.005 mol, 0.28 g) was continued for 6 h. Then chloroacetone (0.002 mol, 0.164 ml) was added and continue stirring overnight. Pouring the mixture into ice-cold water. Finally, the solid underwent filtration, drying, and recrystallized by heating in ethanol.

Gray solid; yield (43%); m.p. 168–170 °C. IR ($\overline{\nu }$/cm^−1^): 3405 (N–H), 2176 (C≡N), 1634(C=O). ^1^H NMR (δ/ppm): 1.28 (s, 3H, CH_3_), 2.81 (s, 6H, –N(Me)_2_), 6.62 (d, *J* = 8.50 Hz, 2H, Ar–H), 6.87 (s, 1H, CH), 7.23–7.32 (m, 3H, Ar–H), 7.35–7.41 (m, 1H, Ar–H), 7.41–7.50 (m, 3H, Ar–H), 8.56 (s, 1H, NH). ^13^C NMR (δ/ppm): 25.76, 40.38, 40.55, 70.89, 95.53, 112.46 (2C), 116.02, 122.13 (2C), 128.58, 128.90 (2C), 129.13 (2C), 130.28 (2C), 137.52, 147.13, 163.89, 169.80. Analysis Calcd. for C_21_H_20_N_4_OS (376.14): C, 67.00; H, 5.35; N, 14.88%; found: C, 66.87; H, 5.30; N, 14.96%.

### Synthesis 2-cyano-*N*-(4-(dimethylamino)phenyl)-2-(4-oxo-3-phenylthiazolidin-2-ylidene)acetamide (3)

Stirring of cyanoacetamide compound **1** (0.005 mol, 1.02 g) with phenyl isothiocyanate (0.005 mol, 0.60 ml) in 20 ml DMF containing KOH (0.005 mol, 0.28 g) was continued for 6 h. Then ethyl bromoacetate (0.002 mol, 0.84 ml) was added and continue stirring overnight. For precipitation, pouring the mixture into ice-cold water, then filtrated, and recrystallized by heating in ethanol.

Yellow crystals; yield (33%); m.p. 138–140 °C. IR ($\overline{\nu }$/cm^−1^): 3368 (N–H), 2191 (C≡N), 1714, 1622 (C=O). ^1^H NMR (δ/ppm): 2.82 (s, 6H, –N(Me)_2_), 3.97 (s, 2H, CH_2_), 6.63 (d, *J* = 9.00 Hz, 2H, Ar–H), 7.19 (t, *J* = 7.50 Hz, 1H, Ar–H), 7.24 (d, *J* = 9.00 Hz, 2H, Ar–H), 7.30 (d, *J* = 9.00 Hz, 2H, Ar–H), 7.33–7.39 (m, 2H, Ar–H), 9.34 (s, 1H, NH). ^13^C NMR (δ/ppm): 33.25, 40.47 (2C), 77.54, 112.67 (2C), 114.81, 122.06 (2C), 127.84 (2C), 128.29, 128.93, 129.75 (2C), 138.17, 148.94, 162.18, 169.33, 170.69. Analysis Calcd. for C_20_H_18_N_4_O_2_S (378.12): C, 63.47; H, 4.79; N, 14.80%; found: C, 63.68; H, 4.72; N, 14.91%.

### Synthesis of 2-cyano-*N-*(4-(dimethylamino)phenyl)-3-mercapto-3-(phenylamino)acrylamide (4)

A suspension of cyanoacetamide compound **1** (0.005 mol, 1.02 g) and phenyl isothiocyanate (0.005 mol, 0.60 ml) was stirred in 20 ml DMF containing KOH (0.005 mol, 0.28 g) for 6 h. Firstly, the mixture was diluted with cold water, then acidified with diluted hydrochloric acid. The precipitate was filtered and washed with cold ethyl alcohol.

Pale green solid; yield (79%); m.p. 208–210 °C. IR (ν/cm^−1^): 3411 (N–H), 2169 (C≡N), 1641 (C=O). ^1^H NMR (δ/ppm): 2.98 (s, 6H, –N(Me)_2_), 6.85 (d, *J* = 8.50 Hz, 2H, Ar–H), 6.93 (t, *J* = 7.50 Hz, 1H, Ar–H), 7.16–7.22 (m, 4H, Ar–H), 7.45 (d, *J* = 8.50 Hz, 2H, Ar–H), 10.06 (s, 1H, NH), 10.75 (s, 1H, NH). ^13^C NMR (δ/ppm): 40.64 (2C), 83.96, 113.21 (2C), 114.42, 122.45 (2C), 124.46 (2C), 125.33, 127.36, 129.60 (2C), 136.79, 150.08, 164.80, 167.28. Mass analysis (m/z, %): 338 (M^+^, 32.7%). Analysis Calcd. for C_18_H_18_N_4_OS (338.12): C, 63.88; H, 5.36; N, 16.56%; found: C, 64.94; H, 6.41; N, 16.51%.

### Preparation of 5-acetyl-4-amino-*N*-(4-(dimethylamino)phenyl)-2-(phenylamino)thiophene-3-carboxamide (5)

A suspension of acrylamide compound **4** (0.002 mol, 0.67 g), chloroacetone (0.002 mol, 0.16 ml), and 0.5 ml triethylamine was refluxed in ethanol for 2 h. The solid that formed was collected to produce the targeted 5-acetyl-4-aminothiophene compound **5**.

Off white powder; yield (38%); m.p. 218–220 °C. IR (ν/cm^−1^): 3443, 3282 (NH_2_, N–H), 1698, 1639 (C=O). ^1^H NMR (δ/ppm): 2.09 (s, 3H, CH_3_), 2.83 (s, 6H, –N(Me)_2_), 6.68 (d, *J* = 8.50 Hz, 2H), 7.08 (t, *J* = 7.50 Hz, 1H), 7.30–7.45 (m, 4H), 7.43 (d, *J* = 9.00 Hz, 2H), 7.47 (br. s, 2H, NH_2_), 9.51 (s, 1H, NH), 9.77 (s, 1H, NH). ^13^C NMR (δ/ppm): 28.05, 40.52 (2C), 96.27, 106.40, 112.36 (2C), 119.97 (2C), 121.88 (2C), 123.74, 128.38, 129.38 (2C), 141.00, 147.33, 154.38, 156.80, 161.86, 186.70. Analysis Calcd. for C_21_H_22_N_4_O_2_S (394.15): C, 63.94; H, 5.62; N, 14.20%; found: C, 63.89; H, 5.58; N, 14.18%.

### Synthesis of 4-amino-5-benzoyl-*N*-(4-(dimethylamino)phenyl)-2-(phenyl amino)thiophene -3-carboxamide (6)

A solution of acrylamide compound **4** (0.002 mol, 0.67 g) was refluxed with phenacyl bromide (0.002 mol, 0.39 g) and triethylamine (0.5 ml) in ethanol for 2 h. The solid that formed upon cooling was collected and dried to furnish the targeted aminothiophene compound **6**.

Yellow powder; yield (89%); m.p. 198–200 °C. IR ($\overline{\nu }$/cm^−1^): 3405, 3284 (NH_2_ and N–H), 1650, 1616 (C=O). ^1^H NMR (δ/ppm): 2.84 (s, 6H, –N(Me)_2_), 6.70 (br. s, 2H, NH_2_), 7.05–7.08 (m, 1H, Ar–H), 7.28–7.35 (m, 4H, Ar–H), 7.42–7.47 (m, 5H, Ar–H), 7.56–7.58 (dd, *J*_*1*_ = 8.00, *J*_2_ = 2.50 Hz, 2H, Ar–H), 7.88 (s, 2H, NH_2_), 8.07 (s, 2H, Ar–H), 9.62 (s, 1H, NH), 9.82 (s, 1H, NH). ^13^C NMR (δ/ppm): 40.65 (2C), 95.83, 106.22, 112.51 (2C), 119.95 (2C), 122.10 (2C), 123.58, 128.26, 128.76 (2C), 129.40 (2C), 129.64 (2C), 132.16, 137.20, 139.94, 146.03, 153.98, 157.18, 161.78, 185.17. Analysis Calcd. for C_26_H_24_N_4_O_2_S (456.16): C, 68.40; H, 5.30; N, 12.27%; found: C, 68.25; H, 5.36; N, 12.20%.

### Preparation of ethyl 4-amino-3-((4-(dimethylamino)phenyl)carbamoyl)-2-(phenylamino)thiophene-5-carboxylate (7)

Acrylamide compound **4** (0.002 mol, 0.67 g) was refluxed with ethyl bromoacetate (0.002 mol, 0.22 ml) and triethylamine (0.5 ml) in ethanol for 2 h. The formed precipitate underwent filtration, was left to dry, then recrystallized from ethyl alcohol.

Gray powder; yield (89%); m.p. 158–160 °C. IR ($\overline{\nu }$/cm^−1^): 3352, 3293 (NH_2_, N–H), 1740 (C=O), 1663 (C=O). ^1^H NMR (δ/ppm): 1.34 (t, *J* = 7.00 Hz, 3H, CH_3_), 2.94 (s, 6H, –N(Me)_2_), 4.28 (q, *J* = 7.00 Hz, 2H, CH_2_), 5.67 (s, 2H, NH_2_), 6.74 (d, *J* = 9.00 Hz, 2H, Ar–H), 7.10 (t, *J* = 7.50 Hz, 1H, Ar–H), 7.28–7.31 (m, 2H, Ar–H), 7.35–7.38 (m, 4H, Ar–H), 8.35 (s,1H, NH), 10.83 (s, 1H, NH). ^13^C NMR (δ/ppm): 14.58, 40.63 (2C), 59.05, 106.10, 112.46 (2C), 118.54, 119.92 (2C), 121.90 (2C), 123.67, 128.30, 129.36 (2C), 137.86, 140.98, 154.04, 157.15, 162.83, 163.51. Analysis Calcd. for C_22_H_24_N_4_O_3_S (424.16): C, 62.25; H, 5.70; N, 13.20%; found: C, 62.38; H, 5.74; N, 13.15%.

### General procedure for the synthesis of α,β-unsaturated nitrile derivatives 8a–f

A suspension of cyanoacetamide scaffold **1** (0.001 mol, 0.203 g) and the appropriate aromatic aldehyde (0.001 mol) [namely; 4-methylbenzaldehyde, 4-methoxybenzaldehyde, 4-chlorobenzaldehyde, 2-(4-formylphenoxy)-*N-(p*-tolyl)acetamide, 2-(4-formylphenoxy)-*N*-(4-methoxyphenyl)acetamide, *N*-(4-chlorophenyl)-2-(4-formylphenoxy)acetamide] was refluxed for 2 h in absolute ethanol containing drops of piperidine. The precipitate was filtered and dried to produce the corresponding unsaturated nitriles **8a**–**f**.

#### 2-Cyano-*N*-(4-(dimethylamino)phenyl)-3-(*p*-tolyl)acrylamide (8a)

Red crystals; yield (81%); m.p. 198–200 °C. IR ($\overline{\nu }$/cm^−1^): 3381 (N–H), 2206 (C≡N), 1673 (C=O). ^1^H NMR (δ/ppm): 2.38 (s, 3H, CH_3_), 2.86 (s, 6H, –N(Me)_2_), 6.71 (d, *J* = 8.50 Hz, 2H, Ar–H), 7.39 (d, *J* = 8.50 Hz, 2H, Ar–H), 7.46 (d, *J* = 8.50 Hz, 2H, Ar–H), 7.88 (d, *J* = 8.00 Hz, 2H, Ar–H), 8.17 (s, 1H, C=CH), 10.07 (s, 1H, NH). ^13^C NMR (δ/ppm): 21.17, 40.48 (2C), 106.81, 112.41 (2C), 116.23, 122.13 (2C), 127.56, 128.96 (2C), 129.52 (2C), 131.63, 138.39, 147.88, 150.70, 165.08. Mass analysis (m/z, %): 305.25 (M^+^, 36.05%), 68.78 (69.89), 59.91 (100.0), 47.92 (51.88), 45.94 (49.28), 43.98 (51.08), 43.01 (87.65), 40.24 (71.48). Analysis Calcd. for C_19_H_19_N_3_O (305.15): C, 74.73; H, 6.27; N, 13.76%; found: C, 74.59; H, 6.20; N, 13.64%.

#### 3-(*p*-Anisyl)-2-cyano-*N*-(4-(dimethylamino)phenyl)acrylamide (8b)

Orange crystals; yield (85%); m.p. 205–206 °C. IR ($\overline{\nu }$/cm^−1^): 3354 (N–H), 2206 (C≡N), 1672 (C=O). ^1^H NMR (δ/ppm): 2.86 (s, 6H, –N(Me)_2_), 3.85 (s, 3H, OCH_3_), 6.71 (d, *J* = 9.00 Hz, 2H, Ar–H), 7.15 (d, *J* = 9.00 Hz, 2H, Ar–H), 7.45 (d, *J* = 9.00 Hz, 2H, Ar–H), 7.99 (d, *J* = 9.00 Hz, 2H, Ar–H), 8.14 (s, 1H, C=CH), 9.99 (s, 1H, NH). ^13^C NMR (δ/ppm): 40.54 (2C), 56.02, 106.90, 112.63 (2C), 114.33 (2C), 116.18, 122.29 (2C), 124.92, 127.65, 130.48 (2C), 148.14, 150.58, 159.72, 164.87. Mass analysis (m/z, %): 321.20 (M^+^, 23.58%), 292.29 (100.0), 285.97 (97.91), 256.37 (79.44), 145.25 (75.57), 125.68 (68.39), 109.92 (73.09), 59.86 (77.72). Analysis Calcd. for C_19_H_19_N_3_O_2_ (321.15): C, 71.01; H, 5.96; N, 13.08%; found: C, C, 70.90; H, 5.89; N, 12.85%.

#### 3-(4-Chlorophenyl)-2-cyano-*N*-(4-(dimethylamino)phenyl)acrylamide (8c)

Yellow powder; yield (87%); m.p. 248–250 °C. IR ($\overline{\nu }$/cm^−1^): 3321 (N–H), 2224 (C≡N), 1670 (C=O). ^1^H NMR (δ/ppm): 2.86 (s, 6H, –N(Me)_2_), 6.71 (d, *J* = 8.50 Hz, 2H, Ar–H), 7.46 (d, *J* = 9.00 Hz, 2H, Ar–H), 7.67 (d, *J* = 9.00 Hz, 2H, Ar–H), 7.98 (d, *J* = 8.50 Hz, 2H, Ar–H), 8.21 (s, 1H, C=CH), 10.14 (s, 1H, NH). ^13^C NMR (δ/ppm): 40.57 (2C), 106.83, 112.74 (2C), 116.09, 122.35 (2C), 127.57, 128.78 (2C), 131.11 (2C), 132.40, 133.73, 147.96, 150.64, 164.90. Mass analysis (m/z, %): 326.15 (M^+^, 30.94%), 324.05 (79.78), 309.90 (93.05), 268.94 (64.34), 252.80 (80.02), 142.10 (83.88), 73.07 (71.04), 71.13 (100.0). Analysis Calcd. for C_18_H_16_ClN_3_O (325.10): C, 66.36; H, 4.95; N, 12.90%; found: C, 66.24; H, 4.89; N, 12.81%.

#### 2-Cyano-*N*-(4-(dimethylamino)phenyl)-3-(4-(2-oxo-2-(*p-*tolylamino)ethoxy)-phenyl)acrylamide (8d)

Orange powder; yield (95%); m.p. 258–260 °C. IR ($\overline{\nu }$/cm^−1^): 3407, 3338 (N–H), 2213 (C≡N), 1696, 1670 (C=O). ^1^H NMR (δ/ppm): 2.24 (s, 3H, CH_3_), 2.86 (s, 6H, –N(Me)_2_), 4.81 (s, 2H, CH_2_), 6.71 (d, *J* = 9.00 Hz, 2H, Ar–H), 7.12 (d, *J* = 8.50 Hz, 2H, Ar–H), 7.19 (d, *J* = 9.00 Hz, 2H, Ar–H), 7.45 (d, *J* = 9.00 Hz, 2H, Ar–H), 7.50 (d, *J* = 9.00 Hz, 2H, Ar–H), 8.00 (d, *J* = 8.50 Hz, 2H, Ar–H), 8.15 (s, 1H, C=CH), 10.00 (s, 1H, NH), 10.08 (s, 1H, NH). ^13^C NMR (δ/ppm): 21.15, 40.51 (2C), 66.79, 105.63, 112.51 (2C), 115.61 (2C), 116.44, 120.32 (2C), 122.17 (2C), 124.97, 127.38, 129.55 (2C), 130.25 (2C), 134.77, 136.49, 147.70, 149.88, 159.84, 161.67, 166.01. Mass analysis (m/z, %): 454.45 (M^+^, 6.32%), 387.22 (62.14), 212.14 (100.00), 125.09 (51.07), 118.12 (45.58), 91.12 (50.40), 77.10 (46.57), 65.11 (46.64). Analysis Calcd. for C_27_H_26_N_4_O_3_ (454.20): C, 71.35; H, 5.77; N, 12.33%; found: C, 71.30; H, 5.74; N, 12.70%.

#### 2-Cyano-*N*-(4-(dimethylamino)phenyl)-3-(4-(2-((4-methoxy phenyl)amino)-2-oxoethoxy)phenyl) acrylamide (8e)

Red powder; yield (93%); m.p. 238–240 °C. IR ($\overline{\nu }$/cm^−1^): 3398 (N–H), 2199 (C≡N), 1685, 1665 (C=O). ^1^H NMR (δ/ppm): 2.86 (s, 6H, –N(Me)_2_), 3.71 (s, 3H, OCH_3_), 4.80 (s, 2H, CH_2_), 6.71 (d, *J* = 9.50 Hz, 2H, Ar–H), 6.89 (d, *J* = 9.50 Hz, 2H, Ar–H), 7.19 (d, *J* = 9.00 Hz, 2H, Ar–H), 7.45 (d, *J* = 9.50 Hz, 2H, Ar–H), 7.53 (d, *J* = 9.00 Hz, 2H, Ar–H), 8.00 (d, *J* = 9.00 Hz, 2H, Ar–H), 8.15 (s, 1H, C=CH), 10.00 (s, 1H, NH), 10.03 (s, 1H, NH). ^13^C NMR (δ/ppm): 40.57 (2C), 55.96, 66.87, 105.53, 112.44 (2C), 114.48 (2C), 115.54 (2C), 116.60, 122.11 (2C), 122.97 (2C), 125.05, 127.35, 130.19 (2C), 131.82, 147.76, 149.79, 159.08, 159.92, 161.84, 166.23. Mass analysis (m/z, %): 470.12 (M^+^, 18.62%), 374.51 (79.57), 276.61 (58.38), 256.62 (100.00), 248.14 (43.70), 213.40 (49.57), 83.70 (31.43), 75.55 (64.69). Analysis Calcd. for C_27_H_26_N_4_O_4_ (454.20): C, 68.92; H, 5.57; N, 11.91%; found: C, 68.82; H, 5.49; N, 11.87%.

#### 3-(4-(2-((4-Chlorophenyl)amino)-2-oxoethoxy)phenyl)-2-cyano-*N*-(4-(dimethylamino)phenyl)acrylamide (8f)

Orange powder; yield (88%); m.p. 258–260 °C. IR ($\overline{\nu }$/cm^−1^): 3407, 3347(N–H), 2211 (C≡N), 1702, 1668 (C=O). ^1^H NMR (δ/ppm): 2.86 (s, 6H, –N(Me)_2_), 4.84 (s, 2H, CH_2_), 6.70 (d, *J* = 9.00 Hz, 2H, Ar–H), 7.19 (d, *J* = 8.50 Hz, 2H, Ar–H), 7.39 (d, *J* = 9.00 Hz, 2H, Ar–H), 7.45 (d, *J* = 9.50 Hz, 2H, Ar–H), 7.65 (d, *J* = 9.50 Hz, 2H, Ar–H), 8.00 (d, *J* = 9.50 Hz, 2H, Ar–H), 8.15 (s, 1H, C=CH), 10.01 (s, 1H, NH), 10.32 (s, 1H, NH). ^13^C NMR (δ/ppm): 40.36 (2C), 66.98, 104.28, 112.37 (2C), 115.50 (2C), 116.93, 121.22 (2C), 122.06 (2C), 125.13, 127.34, 127.76, 128.74 (2C), 132.34 (2C), 137.33, 147.64, 149.66, 160.04, 161.04, 166.15. Analysis Calcd. for C_26_H_23_ClN_4_O_3_ (474.15): C, 65.75; H, 4.88; N, 11.80%; found: C, 65.75; H, 4.88; N, 11.87%.

### General procedure for the synthesis of 3,5-dicyanopyridone derivatives 9a–f

To a solution of each α,β-unsaturated nitrile derivative **8a**–**f** (0.001 mol) in 10 ml dioxane, malononitrile (0.001 mol, 0.07 g) and two drops of piperidine were added. The mixture was refluxed for 4–6 h. The solid that produced was filtered to obtain the corresponding pyridine derivatives **9a**–**f**.

#### 6-Amino-3,5-dicyano-1-(4-(dimethylamino)phenyl)-2-oxo-4-(*p*-tolyl)-1,2-dihydropyridine (9a)

Yellow powder; yield (39%); m.p. over 300 °C. IR ($\overline{\nu }$/cm^−1^): 3272, 3184 (NH_2_), 2216 (C≡N), 1664 (C=O). ^1^H NMR (δ/ppm): 2.39 (s, 3H, CH_3_), 2.97 (s, 6H, –N(Me)_2_), 6.83 (d, *J* = 9.00 Hz, 2H, Ar–H), 7.11 (d, *J* = 9.00 Hz, 2H, Ar–H), 7.36 (d, *J* = 8.00 Hz, 2H, Ar–H), 7.41 (d, *J* = 8.00 Hz, 2H, Ar–H). ^13^C NMR (δ/ppm): 20.99, 39.50 (2C), 66.36, 74.91, 88.05, 113.17 (2C), 115.90, 116.66, 121.40, 127.95 (2C), 128.73 (2C), 129.19 (2C), 131.82, 140.13, 150.94, 157.72, 159.96, 161.00. Analysis Calcd. for C_22_H_19_N_5_O (369.16): C, 71.53; H, 5.18; N, 18.96%; found: C, 71.73; H, 5.10; N, 18.1%.

#### 6-Amino-4-(*p*-anisyl)-3,5-dicyano-1-(4-(dimethylamino)phenyl)-2-oxo-1,2-dihydropyridine (9b)

Yellow powder; yield (46%); m.p. over 300 °C. IR ($\overline{\nu }$/cm^−1^): 3263, 3181 (NH_2_), 2214 (C≡N), 1661 (C=O). ^1^H NMR (δ/ppm): 2.97 (s, 6H, –N(Me)_2_), 3.83 (s, 3H, OCH_3_), 6.84 (d, *J* = 13 Hz, 2H, Ar–H), 7.09 (d, *J* = 5.5 Hz, 2H, Ar–H), 7.11 (d, *J* = 4.5 Hz, 2H, Ar–H), 7.49 (d, *J* = 12 Hz, 2H, Ar–H). Mass analysis (m/z, %): 385.83 (M^+^, 23.74%), 369.93 (100.00), 340.98 (65.94), 320.02 (38.82), 239.72 (41.35), 146.69 (92.94), 121.34 (44.15), 81.69 (35.74). Analysis Calcd. for C_22_H_19_N_5_O_2_ (385.15): C, 68.56; H, 4.97; N, 18.17%; found: C, 68.43; H, 4.88; N, 18.04%.

#### 6-Amino-3,5-dicyano-4-(4-chlorophenyl)-1-(4-(dimethylamino)phenyl)-2-oxo-1,2-dihydropyridine (9c)

Yellow powder; yield (42%); m.p. over 300 °C. IR ($\overline{\nu }$/cm^−1^): 3268, 3180 (NH_2_), 2219 (C≡N), 1661 (C=O). ^1^H NMR (δ/ppm): 2.97 (s, 6H, –N(Me)_2_), 6.84 (d, *J* = 9.00 Hz, 2H, Ar–H), 7.09 (d, *J* = 9.00 Hz, 2H, Ar–H), 7.56 (d, *J* = 9.00 Hz, 2H, Ar–H), 7.65 (d, *J* = 9.00 Hz, 2H, Ar–H). Mass analysis (m/z, %): 391.39 (M^+^, 6.25%), 211.16 (36.82), 185.14 (39.83), 78.12 (42.31), 69.11 (100.00), 57.13 (55.89), 44.08 (81.77), 43.12 (54.55). Analysis Calcd. for C_21_H_16_ClN_5_O (389.10): C, 64.70; H, 4.14; N, 17.96%; found: C, 64.86; H, 4.21; N, 17.86%.

#### 2-((5′-Amino-2′,6′-dicyano-4″-(dimethylamino)-3′-oxo-3′,4′-dihydro-[1,1′:4′,1″-terphenyl]-4-yl)oxy)-*N*-(*p*-tolyl)acetamide (9d)

Yellow powder; yield (48%); m.p. over 300 °C. IR ($\overline{\nu }$/cm^−1^): 3340, 3278, 3183 (NH_2_ and N–H), 2213 (C≡N), 1693, 1659 (C=O). ^1^H NMR (δ/ppm): 2.25 (s, 3H, CH_3_) 2.97 (s, 6H, –N(Me)_2_), 4.78 (s, 2H, CH_2_), 6.83 (d, *J* = 9.00 Hz, 2H, Ar–H), 7.16–7.09 (m, 6H, Ar–H), 7.54–7.50 (m, 4H, Ar–H), 10.07 (s, 1H, NH). Mass analysis (m/z, %): 518.20 (M^+^, 24.25%), 216.88 (60.94), 200.02 (62.47), 195.92 (65.18), 190.34 (100.00), 155.28 (80.86), 118.20 (76.35), 58.91 (53.19). Analysis Calcd. for C_31_H_27_N_5_O_3_ (517.21): C, 71.94; H, 5.26; N, 13.53%; found: C, 71.86; H, 5.20; N, 13.48%.

#### 2-((5′-Amino-2′,6′-dicyano-4″-(dimethylamino)-3′-oxo-3′,4′-dihydro-[1,1′:4′,1″-terphenyl]-4-yl)oxy)-*N*-(*p*-anisyl)acetamide (9e)

Yellow powder; yield (47%); m.p. over 300 °C. IR ($\overline{\nu }$/cm^−1^): 3337, 3285, 3191 (NH_2_ and N–H), 2212(C≡N), 1661 (C=O). ^1^H NMR (δ/ppm): 2.97 (s, 6H, –N(Me)_2_), 3.71 (s, 3H, OCH_3_), 4.76 (s, 2H, CH_2_), 6.83 (d, *J* = 9.00 Hz, 2H, Ar–H), 6.89 (d, *J* = 9.00 Hz, 2H, Ar–H), 7.11 (d, *J* = 8.50 Hz, 2H, Ar–H), 7.16 (d, *J* = 9.00 Hz, 2H, Ar–H), 7.51 (d, *J* = 9.00 Hz, 2H, Ar–H), 7.55 (d, *J* = 8.50 Hz, 2H, Ar–H), 10.02 (s, 1H, NH). Mass analysis (m/z, %): 534.85 (M^+^, 11.25%), 208.33 (100.00), 154.20 (83.73), 125.14 (65.09), 112.28 (75.16), 106.20 (93.18), 79.56 (49.87), 45.42 (60.04). Analysis Calcd. for C_31_H_27_N_5_O_4_ (533.21): C, 69.78; H, 5.10; N, 13.13%; found: C, 69.67; H, 5.02; N, 13.01%.

#### 2-((5′-Amino-2′,6′-dicyano-4″-(dimethylamino)-3′-oxo-3′,4′-dihydro-[1,1′:4′,1″-terphenyl]-4-yl)oxy)-*N*-(4-chlorophenyl)acetamide (9f)

Yellow powder; yield (54%); m.p. over 300 °C. IR ($\overline{\nu }$/cm^−1^): 3407, 3346 (NH_2_ and N–H), 2212 (C≡N), 1702, 1664 (C=O). ^1^H NMR (δ/ppm): 2.97 (s, 6H, –N(Me)_2_), 4.81 (s, 2H, CH_2_), 6.83 (d, *J* = 9.00 Hz, 2H, Ar–H), 7.10 (d, *J* = 9.00 Hz, 2H, Ar–H), 7.15 (d, *J* = 9.00 Hz, 2H, Ar–H), 7.38 (d, *J* = 9.00 Hz, 2H, Ar–H), 7.51 (d, *J* = 8.50 Hz, 2H, Ar–H), 7.69 (d, *J* = 8.50 Hz, 2H, Ar–H), 10.31 (s, 1H, NH). Mass analysis (m/z, %): 538.58 (M^+^, 23.63%), 277.49 (80.64), 266.16 (75.22), 177.04 (66.62), 145.70 (92.77), 79.77 (97.11), 79.26 (100.00), 69.49 (65.21). Analysis Calcd. for C_30_H_24_ClN_5_O_3_ (537.16): C, 66.98; H, 4.50; N, 13.02%; found: C, 66.90; H, 4.53; N, 13.11%.

### Biological activity assays

#### Cell lines and reagents

**HepG2** and **MDA-MB-231** cell lines were purchased from Nawah Scientific Company, Egypt. Cells were grown in DMEM medium (BioWhittaker™) supplemented with bovine serum albumin (10%, Life Science Group L, UK, Cat No: S-001B-BR) and with 100 IU/ml penicillin/streptomycin (100 µg/ml) (Lonza, 17-602E). **Doxorubicin** was obtained from Sigma-Aldrich, solubilized in DMSO and kept at − 20 °C as a stock solution. The tested compounds were prepared in dimethyl sulfoxide (10 mM stock) (DMSO Cat. No. 20385.02, Serva, Heidelberg, Germany) and stored at − 20 °C.

#### Cytotoxic assay

Using the MTT assay to determine the cytotoxicity of tested compounds **1**–**9(a**–**d)**. MTT or 3-(4,5-dimethylthiazol-2-yl)-2,5-diphenyltetrazolium bromide was purchased from SERVA, Germany. The cell lines were introduced into 96-well plates at a density of 4 × 10^4^ cells/well in 100 μl of complete medium (tests were done in duplicates). These plates were incubated for 24 h, 5% CO_2_, at 37 °C for settle down and adhesion. The drug (**Doxorubicin**) solutions were earlier produced in DMSO (control) at 5 and 50 μM concentrations, these medication solutions were administered to the line cells for 48 h after adhesion. MTT (3-(4,5-dimethylthiazoyl)-2,5-diphenyl-tetrazolium bromide (MTT) (5 mg/ml (PBS) Phosphate Buffered Saline) was added, and the plate was incubated for 4 h. After that, acidified via sodium dodecyl sulfate (SDS) solution (10% SDS containing 0.01N HCl in 1× PBS) was used to solubilize formazan crystals. The absorbance was measured after 14 h of incubation at λ570–630 nm by a Biotek plate reader (Gen5™) [48, 49]. According to the initial screening, the cells underwent incubation with serial dilutions (25, 12.5, 6.25, 3.125, 1.56, and 0.78 µM) of compounds **1**–**9**(**a**–**d**) for 48 h, then the viability was determined by using MTT reagent. The IC_50_% of the compounds **2**, **6**, **7**, and **9c** were calculated by using Prism 8.0 Software. The results were shown as percentage of control group (DMSO). Inhibition of cell growth was assessed using the equation:

$$\% inhibition = \frac{ Acontrol - Asample}{{Acontrol}} \times 100.$$

The concentration that kills 50% of cells was identified after incubating the cells with six-point serial dilutions (50, 25, and 12.5 µM) of compounds **2**, **6**, **7**, and **9c**. Doxorubicin was used as reference. The IC_50_ was calculated by Prism Software.

### Estimation of the pharmacokinetic parameters

The pharmacokinetics and drug-likeness properties were predicted for the synthesized compounds were predicted by online tool SwissADME predictor software (http://swissadme.ch/index.php) made by Swiss Institute of Bioinformatics. The 2D chemical structures of the tested compounds was copied into SMILEY mode from chemdraw program. The boiled-egg was generated based on the properties of each compound.

### PASS online predictor for anticancer activity

The tool used for prediction is (PASS) (http://www.way2drug.com/passonline/predict.php). Prediction of activity spectra for substance. The 2D chemical structures of the tested compounds was copied into SMILEY mode from chemdraw program. Pa (probability to be active) and Pi (probability to be inactive) values were given by PASS online software for different anti-cancer activities and IDE inhibition.

### Molecular docking study

All the molecular modeling studies were carried out using Molecular Operating Environment (MOE, 2015.01) software. The three-dimensional structure (3D) of the selected proteins (PDB ID 4y72), (PDB ID 2ra3) were downloaded from the PDB website. The water molecules and repeated chains were removed. Protons were added and the energy of the protein was minimized. The preparation of compounds for docking were carried out by energy minimization and potential energy calculation inside MOE program. MOE conducted the docking of the newly synthesized compounds, calculated the binding energies, and provided their binding modes.

### Supplementary Information

**Additional file 1**: **Table S1**. Pharmaceutical prediction of in silico ADMET properties compounds 1-9f. **Table S2**. Computational prediction of the biological activity spectrum for compounds 1-9f.**Fig. S1**. The binding interaction of 1 with (PDB ID: 4y72). **Fig. S2** The binding interaction of 2 with (PDB ID: 4y72).**Fig. S3** The binding interaction of 3 with (PDB ID: 4y72).**Fig. S4** The binding interaction of 4 with (PDB ID: 4y72).**Fig. S5** The binding interaction of 5 with (PDB ID: 4y72). **Fig. S6** The binding interaction of 6 with (PDB ID: 4y72).**Fig. S7** The binding interaction of 7with (PDB ID: 4y72). Fig. S8 The binding interaction of 8awith (PDB ID: 4y72).**Fig. S9 **The binding interaction of 8b with (PDB ID: 4y72). Fig. S10 The binding interaction of 8c with (PDB ID: 4y72). **Fig. S11** The binding interaction of 8d with (PDB ID: 4y72). **Fig. S12** The binding interaction of 8e with (PDB ID: 4y72). **Fig. S13** The binding interaction of 8f with (PDB ID: 4y72). **Fig. S14** The binding interaction of 9awith (PDB ID: 4y72). **Fig. S15** The binding interaction of 9b with (PDB ID: 4y72). **Fig. S16** The binding interaction of 9c with (PDB ID: 4y72). **Fig. S17** The binding interaction of 9d with (PDB ID: 4y72). **Fig. S18** The binding interaction of 9e with (PDB ID: 4y72). **Fig. S19** The binding interaction of 9f with (PDB ID: 4y72). **Fig. S20** The binding interaction Doxorubicin with (PDB ID: 4y72). **Fig. S21** The binding interaction of 1 with (PDB ID: 2ra3). **Fig. S22** The binding interaction of 2 with (PDB ID: 2ra3). **Fig. S23** The binding interaction of 3 with (PDB ID: 2ra3). **Fig. S24** The binding interaction of 4 with (PDB ID: 2ra3). **Fig. S25** The binding interaction of 5 with (PDB ID: 2ra3). **Fig. S26** The binding interaction of 6 with (PDB ID: 2ra3). **Fig. S27** The binding interaction of 7with (PDB ID: 2ra3). **Fig. S28** The binding interaction of 8awith (PDB ID: 2ra3). **Fig. S29** The binding interaction of 8b with (PDB ID: 2ra3). **Fig. S30** The binding interaction of 8c with (PDB ID: 2ra3). **Fig. S31** The binding interaction of 8d with (PDB ID: 2ra3). **Fig. S32** The binding interaction of 8e with (PDB ID: 2ra3 ). **Fig. S33** The binding interaction of 8f with (PDB ID: 2ra3). **Fig. S34** The binding interaction of 9awith (PDB ID: 2ra3). **Fig. S35** The binding interaction of 9b with (PDB ID: 2ra3). **Fig. S36** The binding interaction of 9c with (PDB ID: 2ra3). **Fig. S37** The binding interaction of 9d with (PDB ID: 2ra3). **Fig. S38** The binding interaction of 9e with (PDB ID: 2ra3). **Fig. S39** The binding interaction of 9f with (PDB ID: 2ra3). **Fig. S40** The binding interaction Doxorubicin with (PDB ID: 2ra3). **Table S3**. Interaction between drugs 1-9(a-f) and target proteins (4y72 and 2ra3) and their docking scores. **Table S4**. Cell viability and growth inhibition percent after treatment of cells with 25 µM of the tested compounds. **Fig S41**. IC50% for the compound 2 against HepG2, MDA-MB-231 cell lines. **Fig. S42** Microscopic images of HepG2 cells following 48 h of exposure to compounds 2, 6, 7, and 9c with different concentrations (50, 25, and 12.5 µM). **Figure S43**. Microscopic images of MDA-MB-231 cells following 48 h of exposure to exposure to compounds 2, 6, 7, and 9c with different concentrations (50, 25, and 12.5 μM). **Fig. S44** 1H-NMR spectrum of compound 1. **Fig. S45** 13C-NMR spectrum of compound 1. **Fig. S46** 1H-NMR spectrum of compound 2. **Fig. S47** 13C-NMR spectrum of compound 2. **Fig. S48** 1H-NMR spectrum of compound 3. **Fig. S49** 13C-NMR spectrum of compound 3. **Fig. S50** 1H-NMR spectrum of compound 4. **Fig. S51** 13C-NMR spectrum of compound 4. **Fig. S52** 1H-NMR spectrum of compound 5. **Fig. S53** 13C-NMR spectrum of compound 5. **Fig. S54** 1H-NMR spectrum of compound 6. **Fig. S55** 13C-NMR spectrum of compound 6. **Fig. S56** 1H-NMR spectrum of compound 7. **Fig. S57** 13C-NMR spectrum of compound 7. **Fig. S58** 1H-NMR spectrum of compound 8a. **Fig. S59** 13C-NMR spectrum of compound 8a. **Fig. S60** 1H-NMR spectrum of compound 8b. **Fig. S61** 13C-NMR spectrum of compound 8b. **Fig. S62** 1H-NMR spectrum of compound 8c. **Fig. S63** 13C-NMR spectrum of compound 8c. **Fig. S64** 1H-NMR spectrum of compound 8d. **Fig. S65** 13C-NMR spectrum of compound 8d. **Fig. S66** 1H-NMR spectrum of compound 8e. **Fig. S67** 13C-NMR spectrum of compound 8e. **Fig. S68** 1H-NMR spectrum of compound 8f. **Fig. S69** 13C-NMR spectrum of compound 8f. **Fig. S70** 1H-NMR spectrum of compound 9a. **Fig. S71** 1H-NMR spectrum of compound 9b. **Fig. S72** 1H-NMR spectrum of compound 9c. **Fig. S73** 1H-NMR spectrum of compound 9d. **Fig. S74** 1H-NMR spectrum of compound 9e. **Fig. S75** 1H-NMR spectrum of compound 9f.

## Data Availability

The datasets used and/or analyzed during the current study available from the corresponding author on reasonable request.
