# Supplementary material for: New thiazole, thiophene and 2-pyridone compounds incorporating dimethylaniline moiety: synthesis, cytotoxicity, ADME and molecular docking studies
Source: BMC Chem. 2024 Mar 14;18(1):52. doi: 10.1186/s13065-024-01136-z (PMC10941513; doi:10.1186/s13065-024-01136-z)
Supplement: Supplementary file 1 — Additional file 1: Table S1. Pharmaceutical prediction of in silico ADMET properties compounds 1-9f. Table S2. Computational prediction of the biological activity spectrum for compounds 1-9f.Fig. S1. The binding interaction of 1 with (PDB ID: 4y72). Fig. S2 The binding interaction of 2 with (PDB ID: 4y72).Fig. S3 The binding interaction of 3 with (PDB ID: 4y72).Fig. S4 The binding interaction of 4 with (PDB ID: 4y72).Fig. S5 The binding interaction of 5 with (PDB ID: 4y72). Fig. S6 The binding interaction of 6 with (PDB ID: 4y72).Fig. S7 The binding interaction of 7with (PDB ID: 4y72). Fig. S8 The binding interaction of 8awith (PDB ID: 4y72).Fig. S9 The binding interaction of 8b with (PDB ID: 4y72). Fig. S10 The binding interaction of 8c with (PDB ID: 4y72). Fig. S11 The binding interaction of 8d with (PDB ID: 4y72). Fig. S12 The binding interaction of 8e with (PDB ID: 4y72). Fig. S13 The binding interaction of 8f with (PDB ID: 4y72). Fig. S14 The binding interaction of 9awith (PDB ID: 4y72). Fig. S15 The binding interaction of 9b with (PDB ID: 4y72). Fig. S16 The binding interaction of 9c with (PDB ID: 4y72). Fig. S17 The binding interaction of 9d with (PDB ID: 4y72). Fig. S18 The binding interaction of 9e with (PDB ID: 4y72). Fig. S19 The binding interaction of 9f with (PDB ID: 4y72). Fig. S20 The binding interaction Doxorubicin with (PDB ID: 4y72). Fig. S21 The binding interaction of 1 with (PDB ID: 2ra3). Fig. S22 The binding interaction of 2 with (PDB ID: 2ra3). Fig. S23 The binding interaction of 3 with (PDB ID: 2ra3). Fig. S24 The binding interaction of 4 with (PDB ID: 2ra3). Fig. S25 The binding interaction of 5 with (PDB ID: 2ra3). Fig. S26 The binding interaction of 6 with (PDB ID: 2ra3). Fig. S27 The binding interaction of 7with (PDB ID: 2ra3). Fig. S28 The binding interaction of 8awith (PDB ID: 2ra3). Fig. S29 The binding interaction of 8b with (PDB ID: 2ra3). Fig. S30 The binding interaction of 8c with (PDB ID: 2ra3). Fig. S31 The binding interaction of [file 13065_2024_1136_MOESM1_ESM.docx]

**Supporting Information**

**New Thiazole, Thiophene and 2-Pyridone Compounds Incorporating Dimethylaniline Moiety: Synthesis, Cytotoxicity, ADME and Molecular Docking Studies**

Heba M. Metwally^1^*, Norhan. M. Younis^1^, Ehab. Abdel-Latif^1^ and Ali El-Rayyes^2^*

^1^ Department of Chemistry, Faculty of Science, Mansoura University, Mansoura 35516, Egypt.

^2^ Department of Chemistry, Faculty of Science, Northern Border University, Arar 1321, Saudi Arabia.

**Corresponding author E-mail:* [*hebama@mans.edu.eg*](mailto:hebama@mans.edu.eg)*;* [*ali.elrayyes@nbu.edu.sa*](mailto:ali.elrayyes@nbu.edu.sa)

**Table S1.** Pharmaceutical prediction of in silico ADMET properties compounds **1-9f**. **Table S2.** Computational prediction of the biological activity spectrum for compounds **1-9f**. **Fig. S1.** The binding interaction of **1** with (PDB ID: 4y72). **Fig. S2** The binding interaction of **2** with (PDB ID: 4y72). **Fig. S3** The binding interaction of **3** with (PDB ID: 4y72). **Fig. S4** The binding interaction of **4** with (PDB ID: 4y72). **Fig. S5** The binding interaction of **5** with (PDB ID: 4y72).

**Fig. S6** The binding interaction of **6** with (PDB ID: 4y72). **Fig. S7** The binding interaction of **7**with (PDB ID: 4y72). **Fig. S8** The binding interaction of **8a**with (PDB ID: 4y72). **Fig. S9** The binding interaction of **8b** with (PDB ID: 4y72). **Fig. S10** The binding interaction of **8c** with (PDB ID: 4y72). **Fig. S11** The binding interaction of **8d** with (PDB ID: 4y72). **Fig. S12** The binding interaction of **8e** with (PDB ID: 4y72). **Fig. S13** The binding interaction of **8f** with (PDB ID: 4y72). **Fig. S14** The binding interaction of **9a**with (PDB ID: 4y72). **Fig. S15** The binding interaction of **9b** with (PDB ID: 4y72). **Fig. S16** The binding interaction of **9c** with (PDB ID: 4y72). **Fig. S17** The binding interaction of **9d** with (PDB ID: 4y72). **Fig. S18** The binding interaction of **9e** with (PDB ID: 4y72). **Fig. S19** The binding interaction of **9f** with (PDB ID: 4y72). **Fig. S20** The binding interaction Doxorubicin with (PDB ID: 4y72). **Fig. S21** The binding interaction of **1** with (PDB ID: **2ra3**). **Fig. S22** The binding interaction of **2** with (PDB ID: **2ra3**).

**Fig. S23** The binding interaction of **3** with (PDB ID: **2ra3**). **Fig. S24** The binding interaction of **4** with (PDB ID: **2ra3**). **Fig. S25** The binding interaction of **5** with (PDB ID: **2ra3**). **Fig. S26** The binding interaction of **6** with (PDB ID: **2ra3**). **Fig. S27** The binding interaction of **7**with (PDB ID: **2ra3**). **Fig. S28** The binding interaction of **8a**with (PDB ID: **2ra3**). **Fig. S29** The binding interaction of **8b** with (PDB ID: **2ra3**). **Fig. S30** The binding interaction of **8c** with (PDB ID: **2ra3**). **Fig. S31** The binding interaction of **8d** with (PDB ID: **2ra3**). **Fig. S32** The binding interaction of **8e** with (PDB ID: **2ra3** ). **Fig. S33** The binding interaction of **8f** with (PDB ID: **2ra3**). **Fig. S34** The binding interaction of **9a**with (PDB ID: **2ra3**). **Fig. S35** The binding interaction of **9b** with (PDB ID: **2ra3**). **Fig. S36** The binding interaction of **9c** with (PDB ID: **2ra3**). **Fig. S37** The binding interaction of **9d** with (PDB ID: **2ra3**). **Fig. S38** The binding interaction of **9e** with (PDB ID: **2ra3**). **Fig. S39** The binding interaction of **9f** with (PDB ID: **2ra3**).

**Fig. S40** The binding interaction Doxorubicin with (PDB ID: **2ra3**). **Table S3.** Interaction between drugs **1-9(a-f)** and target proteins (4y72 and 2ra3) and their docking scores. **Table S4.** Cell viability and growth inhibition percent after treatment of cells with 25 µM of the tested compounds. **Fig S41.** IC_50_% for the compound 2 against HepG2, MDA-MB-231 cell lines. **Fig. S42** Microscopic images of HepG2 cells following 48 h of exposure to compounds 2, 6, 7, and 9c with different concentrations (50, 25, and 12.5 µM). **Fig. S43.** Microscopic images of MDA-MB-231 cells following 48 h of exposure to exposure to compounds 2, 6, 7, and 9c with different concentrations (50, 25, and 12.5 µM) **Fig. S44** ^1^H-NMR spectrum of compound **1. Fig. S45** ^13^C-NMR spectrum of compound **1. Fig. S46** ^1^H-NMR spectrum of compound **2. Fig. S47** ^13^C-NMR spectrum of compound **2. Fig. S48** ^1^H-NMR spectrum of compound **3. Fig. S49** ^13^C-NMR spectrum of compound **3. Fig. S50** ^1^H-NMR spectrum of compound **4. Fig. S51** ^13^C-NMR spectrum of compound **4. Fig. S52** ^1^H-NMR spectrum of compound **5. Fig. S53** ^13^C-NMR spectrum of compound **5. Fig. S54** ^1^H-NMR spectrum of compound **6. Fig. S55** ^13^C-NMR spectrum of compound **6. Fig. S56** ^1^H-NMR spectrum of compound **7. Fig. S57** ^13^C-NMR spectrum of compound **7. Fig. S58** ^1^H-NMR spectrum of compound **8a. Fig. S59** ^13^C-NMR spectrum of compound **8a. Fig. S60** ^1^H-NMR spectrum of compound **8b. Fig. S61** ^13^C-NMR spectrum of compound **8b. Fig. S62** ^1^H-NMR spectrum of compound **8c. Fig. S63** ^13^C-NMR spectrum of compound **8c. Fig. S64** ^1^H-NMR spectrum of compound **8d. Fig. S65** ^13^C-NMR spectrum of compound **8d. Fig. S66** ^1^H-NMR spectrum of compound **8e. Fig. S67** ^13^C-NMR spectrum of compound **8e. Fig. S68** ^1^H-NMR spectrum of compound **8f. Fig. S69** ^13^C-NMR spectrum of compound **8f. Fig. S70** ^1^H-NMR spectrum of compound **9a. Fig. S71** ^1^H-NMR spectrum of compound **9b. Fig. S72** ^1^H-NMR spectrum of compound **9c. Fig. S73** ^1^H-NMR spectrum of compound **9d. Fig. S74** ^1^H-NMR spectrum of compound **9e. Fig. S75** ^1^H-NMR spectrum of compound **9f.**

**Computational Study**

**Table S1. Pharmaceutical prediction of in silico ADMET properties of compounds 1-9f**

| **Molecule** | **Canonical SMILES** | **Formula** | **MW** |
| --- | --- | --- | --- |
| **1** | O=C(CC#N)NC1=CC=C(N(C)C)C=C1 | C_11_H_13_N_3_O | 203.2 |
| **2** | N#C/C(=c/1\scc(n1c1ccccc1)C)/C(=O)Nc1ccc(cc1)N(C)C | C_21_H_20_N_4_OS | 376.47 |
| **3** | CN(c1ccc(cc1)NC(=O)/C(=C\1/SCC(=O)N1c1ccccc1)/C#N)C | C_20_H_18_N_4_O_2_S | 378.45 |
| **4** | CN(c1ccc(cc1)NC(=O)/C(=C(\Nc1ccccc1)/S)/C#N)C | C_18_H_18_N_4_OS | 338.43 |
| **5** | O=C(c1c(Nc2ccccc2)sc(c1N)C(=O)C)Nc1ccc(cc1)N(C)C | C_21_H_22_N_4_O_2_S | 394.49 |
| **6** | O=C(c1c(Nc2ccccc2)sc(c1N)C(=O)c1ccccc1)Nc1ccc(cc1)N(C)C | C_26_H_24_N_4_O_2_S | 456.56 |
| **7** | CCOC(=O)c1sc(c(c1N)C(=O)Nc1ccc(cc1)N(C)C)Nc1ccccc1 | C_22_H_24_N_4_O_3_S | 424.52 |
| **8a** | N#C/C(=C\c1ccc(cc1)C)/C(=O)Nc1ccc(cc1)N(C)C | C_19_H_19_N_3_O | 305.37 |
| **8b** | COc1ccc(cc1)/C=C(/C(=O)Nc1ccc(cc1)N(C)C)\C#N | C_19_H_19_N_3_O_2_ | 321.37 |
| **8c** | N#C/C(=C\c1ccc(cc1)Cl)/C(=O)Nc1ccc(cc1)N(C)C | C_18_H_16_ClN_3_O | 325.79 |
| **8d** | N#C/C(=C\c1ccc(cc1)OCC(=O)Nc1ccc(cc1)C)/C(=O)Nc1ccc(cc1)N(C)C | C_27_H_26_N_4_O_3_ | 454.52 |
| **8e** | COc1ccc(cc1)NC(=O)COc1ccc(cc1)/C=C(/C(=O)Nc1ccc(cc1)N(C)C)\C#N | C_27_H_26_N_4_O_4_ | 470.52 |
| **8f** | N#C/C(=C\c1ccc(cc1)OCC(=O)Nc1ccc(cc1)Cl)/C(=O)Nc1ccc(cc1)N(C)C | C_26_H_23_ClN_4_O_3_ | 474.94 |
| **9a** | N#Cc1c(c2ccc(cc2)C)c(C#N)c(=O)n(c1N)c1ccc(cc1)N(C)C | C_22_H_19_N_5_O | 369.42 |
| **9b** | COc1ccc(cc1)c1c(C#N)c(N)n(c(=O)c1C#N)c1ccc(cc1)N(C)C | C_22_H_19_N_5_O_2_ | 385.42 |
| **9c** | N#Cc1c(c2ccc(cc2)Cl)c(C#N)c(=O)n(c1N)c1ccc(cc1)N(C)C | C_21_H_16_ClN_5_O | 389.84 |
| **9d** | N#Cc1c(c2ccc(cc2)OCC(=O)Nc2ccc(cc2)C)c(C#N)c(=O)n(c1N)c1ccc(cc1)N(C)C | C_30_H_26_N_6_O_3_ | 518.57 |
| **9e** | COc1ccc(cc1)NC(=O)COc1ccc(cc1)c1c(C#N)c(N)n(c(=O)c1C#N)c1ccc(cc1)N(C)C | C_30_H_26_N_6_O_4_ | 534.57 |
| **9f** | N#Cc1c(c2ccc(cc2)OCC(=O)Nc2ccc(cc2)Cl)c(C#N)c(=O)n(c1N)c1ccc(cc1)N(C)C | C_29_H_23_ClN_6_O_3_ | 538.98 |
| **Doxorubicin** | OCC(=O)[C@@]1(O)C[C@H](O[C@H]2C[C@H](N)[C@H]([C@@H](O2)C)O)c2c(C1)c(O)c1c(c2O)C(=O)c2c(C1=O)cccc2OC | C_27_H_29_NO_11_ | 543.52 |

| **Molecule** | **MR** | **TPSA** | **iLOGP** | **XLOGP3** | **WLOGP** | **MLOGP** | **Silicos-IT Log P** | **Consensus Log P** | **ESOL Log S** |
| --- | --- | --- | --- | --- | --- | --- | --- | --- | --- |
| **1** | 59.52 | 56.13 | 1.26 | 1.44 | 1.41 | 0.89 | 0.88 | 1.18 | -2.04 |
| **2** | 110.76 | 89.3 | 3.05 | 4.49 | 3.32 | 1.92 | 3.67 | 3.29 | -5.14 |
| **3** | 111 | 101.74 | 2.66 | 3.38 | 2.63 | 1.4 | 2.04 | 2.42 | -4.31 |
| **4** | 100.61 | 106.96 | 2.51 | 4.4 | 3.09 | 1.82 | 1.79 | 2.72 | -4.68 |
| **5** | 116.88 | 115.7 | 3.03 | 4.88 | 4.41 | 1.99 | 3.43 | 3.55 | -5.35 |
| **6** | 136.56 | 115.7 | 3.41 | 6.53 | 5.44 | 2.83 | 4.5 | 4.54 | -6.77 |
| **7** | 122.77 | 124.93 | 3.42 | 5.41 | 4.39 | 2.22 | 3.38 | 3.76 | -5.71 |
| **8a** | 94.1 | 56.13 | 2.85 | 3.9 | 3.31 | 2.61 | 3.08 | 3.15 | -4.25 |
| **8b** | 95.62 | 65.36 | 2.83 | 3.5 | 3.01 | 2.05 | 2.63 | 2.8 | -4.01 |
| **8c** | 94.14 | 56.13 | 2.62 | 4.16 | 3.65 | 2.88 | 3.2 | 3.3 | -4.54 |
| **8d** | 134.42 | 94.46 | 3.19 | 5.02 | 4.13 | 2.53 | 3.89 | 3.75 | -5.55 |
| **8e** | 135.95 | 103.69 | 3.53 | 4.63 | 3.83 | 2.01 | 3.44 | 3.49 | -5.33 |
| **8f** | 134.47 | 94.46 | 3.02 | 5.29 | 4.48 | 2.8 | 4 | 3.92 | -5.85 |
| **9a** | 110.48 | 98.84 | 2.64 | 3.06 | 3.21 | 2.11 | 3.12 | 2.83 | -4.34 |
| **9b** | 112.01 | 108.07 | 2.67 | 2.66 | 2.91 | 1.58 | 2.66 | 2.5 | -4.1 |
| **9c** | 110.53 | 98.84 | 2.6 | 3.32 | 3.56 | 2.37 | 3.23 | 3.02 | -4.63 |
| **9d** | 150.81 | 137.17 | 3.05 | 3.93 | 4.04 | 2.03 | 3.85 | 3.38 | -5.46 |
| **9e** | 152.33 | 146.4 | 3.3 | 3.54 | 3.74 | 1.53 | 3.4 | 3.1 | -5.23 |
| **9f** | 150.85 | 137.17 | 3.36 | 4.2 | 4.38 | 2.3 | 3.96 | 3.64 | -5.76 |
| **Doxorubicin** | 132.66 | 206.07 | 2.5 | 1.27 | -0.32 | -2.1 | 1.17 | 0.5 | -3.91 |

| **Molecule** | **ESOL Solubility (mg/ml)** | **ESOL Solubility (mol/l)** | **ESOL Class** | **Ali Log S** | **Ali Solubility (mg/ml)** | **Ali Solubility (mol/l)** |
| --- | --- | --- | --- | --- | --- | --- |
| **1** | 1.86E+00 | 9.14E-03 | Soluble | -2.22 | 1.21E+00 | 5.97E-03 |
| **2** | 2.74E-03 | 7.26E-06 | Moderately soluble | -6.09 | 3.09E-04 | 8.21E-07 |
| **3** | 1.83E-02 | 4.85E-05 | Moderately soluble | -5.2 | 2.41E-03 | 6.38E-06 |
| **4** | 7.00E-03 | 2.07E-05 | Moderately soluble | -6.36 | 1.47E-04 | 4.33E-07 |
| **5** | 1.77E-03 | 4.49E-06 | Moderately soluble | -7.04 | 3.56E-05 | 9.02E-08 |
| **6** | 7.71E-05 | 1.69E-07 | Poorly soluble | -8.76 | 7.99E-07 | 1.75E-09 |
| **7** | 8.36E-04 | 1.97E-06 | Moderately soluble | -7.79 | 6.91E-06 | 1.63E-08 |
| **8a** | 1.73E-02 | 5.67E-05 | Moderately soluble | -4.78 | 5.10E-03 | 1.67E-05 |
| **8b** | 3.13E-02 | 9.74E-05 | Moderately soluble | -4.56 | 8.94E-03 | 2.78E-05 |
| **8c** | 9.47E-03 | 2.91E-05 | Moderately soluble | -5.05 | 2.93E-03 | 8.98E-06 |
| **8d** | 1.27E-03 | 2.80E-06 | Moderately soluble | -6.74 | 8.19E-05 | 1.80E-07 |
| **8e** | 2.21E-03 | 4.69E-06 | Moderately soluble | -6.53 | 1.38E-04 | 2.93E-07 |
| **8f** | 6.72E-04 | 1.42E-06 | Moderately soluble | -7.02 | 4.49E-05 | 9.46E-08 |
| **9a** | 1.70E-02 | 4.61E-05 | Moderately soluble | -4.8 | 5.83E-03 | 1.58E-05 |
| **9b** | 3.06E-02 | 7.93E-05 | Moderately soluble | -4.58 | 1.01E-02 | 2.62E-05 |
| **9c** | 9.22E-03 | 2.36E-05 | Moderately soluble | -5.07 | 3.30E-03 | 8.47E-06 |
| **9d** | 1.80E-03 | 3.48E-06 | Moderately soluble | -6.51 | 1.60E-04 | 3.09E-07 |
| **9e** | 3.12E-03 | 5.83E-06 | Moderately soluble | -6.3 | 2.68E-04 | 5.02E-07 |
| **9f** | 9.47E-04 | 1.76E-06 | Moderately soluble | -6.79 | 8.74E-05 | 1.62E-07 |
| **Doxorubicin** | 6.72E-02 | 1.24E-04 | Soluble | -5.2 | 3.46E-03 | 6.36E-06 |

| **Molecule** | **Ali Class** | **Silicos-IT LogSw** | **Silicos-IT Solubility (mg/ml)** | **Silicos-IT Solubility (mol/l)** | **Silicos-IT class** | **GI absorption** |
| --- | --- | --- | --- | --- | --- | --- |
| **1** | Soluble | -3 | 2.01E-01 | 9.89E-04 | Soluble | High |
| **2** | Poorly soluble | -6.08 | 3.15E-04 | 8.38E-07 | Poorly soluble | High |
| **3** | Moderately soluble | -5.25 | 2.14E-03 | 5.66E-06 | Moderately soluble | High |
| **4** | Poorly soluble | -5.33 | 1.58E-03 | 4.66E-06 | Moderately soluble | High |
| **5** | Poorly soluble | -6.77 | 6.75E-05 | 1.71E-07 | Poorly soluble | High |
| **6** | Poorly soluble | -8.84 | 6.61E-07 | 1.45E-09 | Poorly soluble | Low |
| **7** | Poorly soluble | -6.89 | 5.52E-05 | 1.30E-07 | Poorly soluble | Low |
| **8a** | Moderately soluble | -5.58 | 8.11E-04 | 2.65E-06 | Moderately soluble | High |
| **8b** | Moderately soluble | -5.31 | 1.58E-03 | 4.92E-06 | Moderately soluble | High |
| **8c** | Moderately soluble | -5.79 | 5.25E-04 | 1.61E-06 | Moderately soluble | High |
| **8d** | Poorly soluble | -8.12 | 3.43E-06 | 7.55E-09 | Poorly soluble | High |
| **8e** | Poorly soluble | -7.85 | 6.71E-06 | 1.43E-08 | Poorly soluble | High |
| **8f** | Poorly soluble | -8.33 | 2.22E-06 | 4.68E-09 | Poorly soluble | High |
| **9a** | Moderately soluble | -6.4 | 1.47E-04 | 3.99E-07 | Poorly soluble | High |
| **9b** | Moderately soluble | -6.13 | 2.88E-04 | 7.46E-07 | Poorly soluble | High |
| **9c** | Moderately soluble | -6.61 | 9.53E-05 | 2.44E-07 | Poorly soluble | High |
| **9d** | Poorly soluble | -8.91 | 6.31E-07 | 1.22E-09 | Poorly soluble | Low |
| **9e** | Poorly soluble | -8.64 | 1.23E-06 | 2.31E-09 | Poorly soluble | Low |
| **9f** | Poorly soluble | -9.12 | 4.09E-07 | 7.59E-10 | Poorly soluble | Low |
| **Doxorubicin** | Moderately soluble | -3.46 | 1.87E-01 | 3.44E-04 | Soluble | Low |

| **Molecule** | **BBB permeant** | **Pgp substrate** | **CYP1A2 inhibitor** | **CYP2C19 inhibitor** | **CYP2C9 inhibitor** | **CYP2D6 inhibitor** | **CYP3A4 inhibitor** | **log Kp (cm/s)** |
| --- | --- | --- | --- | --- | --- | --- | --- | --- |
| 1 | Yes | No | Yes | No | No | No | No | -6.52 |
| 2 | No | No | Yes | Yes | Yes | No | Yes | -5.41 |
| 3 | No | No | No | Yes | Yes | Yes | Yes | -6.21 |
| 4 | No | No | Yes | Yes | Yes | Yes | Yes | -5.24 |
| 5 | No | No | Yes | Yes | Yes | Yes | Yes | -5.24 |
| 6 | No | No | No | Yes | Yes | Yes | Yes | -4.45 |
| 7 | No | No | No | Yes | Yes | Yes | Yes | -5.05 |
| 8a | Yes | No | Yes | Yes | Yes | Yes | Yes | -5.39 |
| 8b | Yes | No | Yes | Yes | Yes | Yes | Yes | -5.78 |
| 8c | Yes | No | Yes | Yes | Yes | Yes | Yes | -5.33 |
| 8d | No | No | No | Yes | Yes | Yes | Yes | -5.51 |
| 8e | No | No | No | Yes | Yes | Yes | Yes | -5.88 |
| 8f | No | No | No | Yes | Yes | Yes | Yes | -5.44 |
| 9a | No | No | Yes | No | Yes | No | Yes | -6.38 |
| 9b | No | No | No | No | Yes | No | Yes | -6.76 |
| 9c | No | No | Yes | No | Yes | No | Yes | -6.32 |
| 9d | No | No | No | Yes | Yes | No | Yes | -6.67 |
| 9e | No | No | No | Yes | Yes | Yes | Yes | -7.05 |
| 9f | No | No | No | Yes | Yes | No | Yes | -6.61 |
| **Doxorubicin** | No | Yes | No | No | No | No | No | -8.71 |

| **Molecule** | **Lipinski #violations** | **Ghose #violations** | **Veber #violations** | **Egan #violations** | **Muegge #violations** | **Bioavailability Score** |
| --- | --- | --- | --- | --- | --- | --- |
| **1** | 0 | 0 | 0 | 0 | 0 | 0.55 |
| **2** | 0 | 0 | 0 | 0 | 0 | 0.55 |
| **3** | 0 | 0 | 0 | 0 | 0 | 0.55 |
| **4** | 0 | 0 | 0 | 0 | 0 | 0.55 |
| **5** | 0 | 0 | 0 | 0 | 0 | 0.55 |
| **6** | 0 | 1 | 0 | 0 | 1 | 0.55 |
| **7** | 0 | 0 | 0 | 0 | 1 | 0.55 |
| **8a** | 0 | 0 | 0 | 0 | 0 | 0.55 |
| **8b** | 0 | 0 | 0 | 0 | 0 | 0.55 |
| **8c** | 0 | 0 | 0 | 0 | 0 | 0.55 |
| **8d** | 0 | 1 | 0 | 0 | 1 | 0.55 |
| **8e** | 0 | 1 | 1 | 0 | 0 | 0.55 |
| **8f** | 0 | 1 | 0 | 0 | 1 | 0.55 |
| **9a** | 0 | 0 | 0 | 0 | 0 | 0.55 |
| **9b** | 0 | 0 | 0 | 0 | 0 | 0.55 |
| **9c** | 0 | 0 | 0 | 0 | 0 | 0.55 |
| **9d** | 1 | 2 | 0 | 1 | 0 | 0.55 |
| **9e** | 1 | 2 | 1 | 1 | 0 | 0.55 |
| **9f** | 1 | 2 | 0 | 1 | 0 | 0.55 |
| **Doxorubicin** | 3 | 2 | 1 | 1 | 3 | 0.17 |

| **Molecule** | **PAINS #alerts** | **Brenk #alerts** | **Leadlikeness #violations** | **Synthetic Accessibility** |
| --- | --- | --- | --- | --- |
| **1** | 1 | 0 | 1 | 1.41 |
| **2** | 1 | 0 | 2 | 3.61 |
| **3** | 1 | 2 | 1 | 3.8 |
| **4** | 1 | 3 | 1 | 3.21 |
| **5** | 1 | 0 | 2 | 3.7 |
| **6** | 1 | 0 | 3 | 4 |
| **7** | 1 | 0 | 3 | 3.92 |
| **8a** | 1 | 2 | 1 | 2.72 |
| **8b** | 1 | 2 | 0 | 2.62 |
| **8c** | 1 | 2 | 1 | 2.62 |
| **8d** | 1 | 2 | 3 | 3.37 |
| **8e** | 1 | 2 | 3 | 3.42 |
| **8f** | 1 | 2 | 3 | 3.26 |
| **9a** | 1 | 0 | 1 | 3.2 |
| **9b** | 1 | 0 | 1 | 3.15 |
| **9c** | 1 | 0 | 1 | 3.08 |
| **9d** | 1 | 0 | 3 | 3.78 |
| **9e** | 1 | 0 | 3 | 3.83 |
| **9f** | 1 | 0 | 3 | 3.66 |
| **Doxorubicin** | 1 | 1 | 1 | 5.81 |

**Table S2. Computational prediction of the biological activity spectrum for compounds 1-9f**

| **Compounds** | | **1** | | | **2** | | | | **3** | | | **4** | |
| --- | --- | --- | --- | --- | --- | --- | --- | --- | --- | --- | --- | --- | --- |
| Biological activity | | Pa | Pi | | Pa | | Pi | | Pa | | Pi | Pa | Pi |
| **Antituberculosic** | | **0.791** | **0.003** | | **0.676** | | **0.004** | | **0.301** | | **0.07** | **0.304** | **0.068** |
| (R)-6-hydroxynicotine oxidase inhibitor | | 0.446 | 0.067 | | 0.248 | | 0.2 | | NA | | NA | NA | NA |
| (R)-aminopropanol dehydrogenase inhibitor | | 0.105 | 0.034 | | NA | | NA | | NA | | NA | NA | NA |
| (R)-limonene 6-monooxygenase inhibitor | | 0.155 | 0.038 | | NA | | NA | | NA | | NA | NA | NA |
| (R)-Pantolactone dehydrogenase (flavin) inhibitor | | 0.339 | 0.075 | | NA | | NA | | NA | | NA | NA | NA |
| (R,R)-butanediol dehydrogenase inhibitor | | 0.187 | 0.05 | | NA | | NA | | NA | | NA | NA | NA |
| (S)-2-hydroxy-acid oxidase inhibitor | | 0.127 | 0.071 | | NA | | NA | | NA | | NA | NA | NA |
| (S)-2-Methylmalate dehydratase inhibitor | | 0.087 | 0.036 | | NA | | NA | | NA | | NA | NA | NA |
| (S)-3-amino-2-methylpropionate transaminase inhibitor | | 0.296 | 0.023 | | 0.195 | | 0.07 | | NA | | NA | NA | NA |
| (S)-3-hydroxyacid ester dehydrogenase inhibitor | | 0.344 | 0.018 | | 0.155 | | 0.079 | | NA | | NA | NA | NA |
| (S)-6-hydroxynicotine oxidase inhibitor | | 0.365 | 0.07 | | NA | | NA | | NA | | NA | NA | NA |
| (S)-carnitine 3-dehydrogenase inhibitor | | 0.118 | 0.061 | | NA | | NA | | NA | | NA | NA | NA |
| [acyl-carrier-protein] S-acetyltransferase inhibitor | | 0.324 | 0.037 | | 0.248 | | 0.072 | | 0.155 | | 0.145 | NA | NA |
| [acyl-carrier-protein] S-malonyltransferase inhibitor | | 0.076 | 0.065 | | NA | | NA | | NA | | NA | NA | NA |
| [myelin basic protein]-arginine N-methyltransferase inhibitor | | 0.084 | 0.049 | | NA | | NA | | NA | | NA | NA | NA |
| 1-Acylglycerol-3-phosphate O-acyltransferase inhibitor | | 0.291 | 0.105 | | NA | | NA | | NA | | NA | NA | NA |
| 1-Alkyl-2-acetyl-glycerophosphocholine esterase inhibitor | | 0.031 | 0.019 | | NA | | NA | | NA | | NA | NA | NA |
| 1-Alkylglycero-phosphocholine O-acetyltransferase inhibitor | | 0.279 | 0.081 | | NA | | NA | | NA | | NA | NA | NA |
| 1-Aminocyclopropane-1-carboxylate deaminase inhibitor | | 0.188 | 0.025 | | NA | | NA | | NA | | NA | NA | NA |
| 1-Deoxy-D-xylulose-5-phosphate reductoisomerase inhibitor | | 0.047 | 0.046 | | NA | | NA | | NA | | NA | NA | NA |
| 1-Phosphofructokinase inhibitor | | 0.054 | 0.047 | | NA | | NA | | NA | | NA | NA | NA |
| 1,4-Alpha-glucan branching enzyme inhibitor | | 0.185 | 0.062 | | 0.148 | | 0.095 | | NA | | NA | NA | NA |
| 1,4-Lactonase inhibitor | | 0.462 | 0.06 | | 0.334 | | 0.148 | | NA | | NA | NA | NA |
| **Compounds** | **5** | | | **6** | | | | **7** | | | | **8a** | |
| Biological activity | Pa | | Pi | Pa | | Pi | | Pa | | Pi | | Pa | Pi |
| **Antituberculosic** | **0.278** | | **0.084** | **0.278** | | **0.084** | | **0.297** | | **0.072** | | **0.412** | **0.028** |
| (R)-6-hydroxynicotine oxidase inhibitor | NA | | NA | NA | | NA | | NA | | NA | | NA | NA |
| (R)-aminopropanol dehydrogenase inhibitor | NA | | NA | NA | | NA | | NA | | NA | | NA | NA |
| (R)-limonene 6-monooxygenase inhibitor | NA | | NA | NA | | NA | | NA | | NA | | NA | NA |
| (R)-Pantolactone dehydrogenase (flavin) inhibitor | NA | | NA | NA | | NA | | NA | | NA | | NA | NA |
| (R,R)-butanediol dehydrogenase inhibitor | NA | | NA | NA | | NA | | NA | | NA | | NA | NA |
| (S)-2-hydroxy-acid oxidase inhibitor | NA | | NA | NA | | NA | | NA | | NA | | NA | NA |
| (S)-2-Methylmalate dehydratase inhibitor | NA | | NA | NA | | NA | | NA | | NA | | NA | NA |
| (S)-3-amino-2-methylpropionate transaminase inhibitor | NA | | NA | NA | | NA | | NA | | NA | | NA | NA |
| (S)-3-hydroxyacid ester dehydrogenase inhibitor | NA | | NA | NA | | NA | | NA | | NA | | NA | NA |
| (S)-6-hydroxynicotine oxidase inhibitor | NA | | NA | NA | | NA | | NA | | NA | | NA | NA |
| (S)-carnitine 3-dehydrogenase inhibitor | NA | | NA | NA | | NA | | NA | | NA | | NA | NA |
| [acyl-carrier-protein] S-acetyltransferase inhibitor | NA | | NA | NA | | NA | | NA | | NA | | NA | NA |
| [acyl-carrier-protein] S-malonyltransferase inhibitor | NA | | NA | NA | | NA | | NA | | NA | | NA | NA |
| [myelin basic protein]-arginine N-methyltransferase inhibitor | NA | | NA | NA | | NA | | NA | | NA | | NA | NA |
| 1-Acylglycerol-3-phosphate O-acyltransferase inhibitor | NA | | NA | NA | | NA | | NA | | NA | | NA | NA |
| 1-Alkyl-2-acetyl glycerophosphocholine esterase inhibitor | NA | | NA | NA | | NA | | NA | | NA | | NA | NA |
| 1-Alkylglycero-phosphocholine O-acetyltransferase inhibitor | NA | | NA | NA | | NA | | NA | | NA | | NA | NA |
| 1-Aminocyclopropane-1-carboxylate deaminase inhibitor | NA | | NA | NA | | NA | | NA | | NA | | NA | NA |
| 1-Deoxy-D-xylulose-5-phosphate reductoisomerase inhibitor | NA | | NA | NA | | NA | | NA | | NA | | NA | NA |
| 1-Phosphofructokinase inhibitor | NA | | NA | NA | | NA | | NA | | NA | | NA | NA |
| 1,4-Alpha-glucan branching enzyme inhibitor | NA | | NA | NA | | NA | | NA | | NA | | NA | NA |
| 1,4-Lactonase inhibitor | NA | | NA | NA | | NA | | NA | | NA | | NA | NA |

| **Compounds** | **8b** | | **8c** | | **8d** | | **8e** | |
| --- | --- | --- | --- | --- | --- | --- | --- | --- |
| Biological activity | Pa | Pi | Pa | Pi | Pa | Pi | Pa | Pi |
| **Antituberculosic** | **0.385** | **0.036** | **0.386** | **0.035** | **0.436** | **0.022** | **0.272** | **0.089** |
| (R)-6-hydroxynicotine oxidase inhibitor | NA | NA | NA | NA | NA | NA | NA | NA |
| (R)-aminopropanol dehydrogenase inhibitor | NA | NA | NA | NA | NA | NA | NA | NA |
| (R)-limonene 6-monooxygenase inhibitor | NA | NA | NA | NA | NA | NA | NA | NA |
| (R)-Pantolactone dehydrogenase (flavin) inhibitor | NA | NA | NA | NA | NA | NA | NA | NA |
| (R,R)-butanediol dehydrogenase inhibitor | NA | NA | NA | NA | NA | NA | NA | NA |
| (S)-2-hydroxy-acid oxidase inhibitor | NA | NA | NA | NA | NA | NA | NA | NA |
| (S)-2-Methylmalate dehydratase inhibitor | NA | NA | NA | NA | NA | NA | NA | NA |
| (S)-3-amino-2-methylpropionate transaminase inhibitor | NA | NA | NA | NA | NA | NA | NA | NA |
| (S)-3-hydroxyacid ester dehydrogenase inhibitor | NA | NA | NA | NA | 0.146 | 0.084 | NA | NA |
| (S)-6-hydroxynicotine oxidase inhibitor | NA | NA | NA | NA | NA | NA | NA | NA |
| (S)-carnitine 3-dehydrogenase inhibitor | NA | NA | NA | NA | NA | NA | NA | NA |
| [acyl-carrier-protein] S-acetyltransferase inhibitor | NA | NA | NA | NA | NA | NA | NA | NA |
| [acyl-carrier-protein] S-malonyltransferase inhibitor | NA | NA | NA | NA | NA | NA | NA | NA |
| [myelin basic protein]-arginine N-methyltransferase inhibitor | NA | NA | NA | NA | NA | NA | NA | NA |
| 1-Acylglycerol-3-phosphate O-acyltransferase inhibitor | NA | NA | NA | NA | 0.259 | 0.135 | NA | NA |
| 1-Alkyl-2-acetylglycerophosphocholine esterase inhibitor | NA | NA | NA | NA | NA | NA | NA | NA |
| 1-Alkylglycerophosphocholine O-acetyltransferase inhibitor | NA | NA | NA | NA | 0.171 | 0.145 | NA | NA |
| 1-Aminocyclopropane-1-carboxylate deaminase inhibitor | NA | NA | NA | NA | NA | NA | NA | NA |
| 1-Deoxy-D-xylulose-5-phosphate reductoisomerase inhibitor | NA | NA | NA | NA | NA | NA | NA | NA |
| 1-Phosphofructokinase inhibitor | NA | NA | NA | NA | NA | NA | NA | NA |
| 1,4-Alpha-glucan branching enzyme inhibitor | NA | NA | NA | NA | NA | NA | NA | NA |
| 1,4-Lactonase inhibitor | NA | NA | NA | NA | 0.265 | 0.241 | NA | NA |

| **Compounds** | **8f** | | **9a** | | **9b** | | **9c** | |
| --- | --- | --- | --- | --- | --- | --- | --- | --- |
| Biological activity | Pa | Pi | Pa | Pi | Pa | Pi | Pa | Pi |
| **Antituberculosic** | **0.781** | **0.003** | **0.796** | **0.003** | **0.782** | **0.003** | **0.794** | **0.003** |
| (R)-6-hydroxynicotine oxidase inhibitor | NA | NA | NA | NA | NA | NA | NA | NA |
| (R)-aminopropanol dehydrogenase inhibitor | NA | NA | NA | NA | NA | NA | NA | NA |
| (R)-limonene 6-monooxygenase inhibitor | NA | NA | NA | NA | NA | NA | NA | NA |
| (R)-Pantolactone dehydrogenase (flavin) inhibitor | NA | NA | NA | NA | NA | NA | NA | NA |
| (R,R)-butanediol dehydrogenase inhibitor | NA | NA | NA | NA | NA | NA | NA | NA |
| (S)-2-hydroxy-acid oxidase inhibitor | NA | NA | NA | NA | NA | NA | NA | NA |
| (S)-2-Methylmalate dehydratase inhibitor | NA | NA | NA | NA | NA | NA | NA | NA |
| (S)-3-amino-2-methylpropionate transaminase inhibitor | NA | NA | NA | NA | NA | NA | NA | NA |
| (S)-3-hydroxyacid ester dehydrogenase inhibitor | NA | NA | NA | NA | NA | NA | NA | NA |
| (S)-6-hydroxynicotine oxidase inhibitor | NA | NA | NA | NA | NA | NA | NA | NA |
| (S)-carnitine 3-dehydrogenase inhibitor | NA | NA | NA | NA | NA | NA | NA | NA |
| [acyl-carrier-protein] S-acetyltransferase inhibitor | NA | NA | NA | NA | NA | NA | NA | NA |
| [acyl-carrier-protein] S-malonyltransferase inhibitor | NA | NA | NA | NA | NA | NA | NA | NA |
| [myelin basic protein]-arginine N-methyltransferase inhibitor | NA | NA | NA | NA | NA | NA | NA | NA |
| 1-Acylglycerol-3-phosphate O-acyltransferase inhibitor | NA | NA | NA | NA | NA | NA | NA | NA |
| 1-Alkyl-2-acetylglycero-phosphocholine esterase inhibitor | NA | NA | NA | NA | NA | NA | NA | NA |
| 1-Alkylglycerophosphocholine O-acetyltransferase inhibitor | NA | NA | NA | NA | NA | NA | NA | NA |
| 1-Aminocyclopropane-1-carboxylate deaminase inhibitor | NA | NA | NA | NA | NA | NA | NA | NA |
| 1-Deoxy-D-xylulose-5-phosphate reductoisomerase inhibitor | NA | NA | NA | NA | NA | NA | NA | NA |
| 1-Phosphofructokinase inhibitor | NA | NA | NA | NA | NA | NA | NA | NA |
| 1,4-Alpha-glucan branching enzyme inhibitor | NA | NA | NA | NA | NA | NA | NA | NA |
| 1,4-Lactonase inhibitor | NA | NA | NA | NA | NA | NA | NA | NA |

| **Compounds** | **9d** | | **9e** | | **9f** | |
| --- | --- | --- | --- | --- | --- | --- |
| Biological activity | Pa | Pi | Pa | Pi | Pa | Pi |
| **Antituberculosic** | **0.546** | **0.009** | **0.675** | **0.004** | **0.454** | **0.018** |
| (R)-6-hydroxynicotine oxidase inhibitor | 0.282 | 0.167 | NA | NA | NA | NA |
| (R)-aminopropanol dehydrogenase inhibitor | 0.128 | 0.022 | NA | NA | NA | NA |
| (R)-limonene 6-monooxygenase inhibitor | 0.107 | 0.078 | NA | NA | NA | NA |
| (R)-Pantolactone dehydrogenase (flavin) inhibitor | 0.234 | 0.18 | NA | NA | NA | NA |
| (R,R)-butanediol dehydrogenase inhibitor | NA | NA | NA | NA | NA | NA |
| (S)-2-hydroxy-acid oxidase inhibitor | NA | NA | NA | NA | NA | NA |
| (S)-2-Methylmalate dehydratase inhibitor | NA | NA | NA | NA | NA | NA |
| (S)-3-amino-2-methylpropionate transaminase inhibitor | 0.194 | 0.071 | 0.201 | 0.065 | 0.141 | 0.136 |
| (S)-3-hydroxyacid ester dehydrogenase inhibitor | 0.196 | 0.055 | NA | NA | NA | NA |
| (S)-6-hydroxynicotine oxidase inhibitor | 0.204 | 0.171 | NA | NA | NA | NA |
| (S)-carnitine 3-dehydrogenase inhibitor | NA | NA | NA | NA | NA | NA |
| [acyl-carrier-protein] S-acetyltransferase inhibitor | 0.242 | 0.075 | 0.19 | 0.111 | 0.171 | 0.129 |
| [acyl-carrier-protein] S-malonyltransferase inhibitor | NA | NA | NA | NA | NA | NA |
| [myelin basic protein]-arginine N-methyltransferase inhibitor | NA | NA | NA | NA | NA | NA |
| 1-Acylglycerol-3-phosphate O-acyltransferase inhibitor | 0.398 | 0.041 | NA | NA | NA | NA |
| 1-Alkyl-2-acetylglycerophosphocholine esterase inhibitor | NA | NA | NA | NA | NA | NA |
| 1-Alkylglycero-phosphocholine O-acetyltransferase inhibitor | 0.166 | 0.15 | NA | NA | NA | NA |
| 1-Aminocyclopropane-1-carboxylate deaminase inhibitor | 0.15 | 0.037 | NA | NA | NA | NA |
| 1-Deoxy-D-xylulose-5-phosphate reductoisomerase inhibitor | NA | NA | NA | NA | NA | NA |
| 1-Phosphofructokinase inhibitor | NA | NA | NA | NA | NA | NA |
| 1,4-Alpha-glucan branching enzyme inhibitor | 0.137 | 0.108 | NA | NA | NA | NA |
| 1,4-Lactonase inhibitor | 0.326 | 0.157 | NA | NA | NA | NA |

**Molecular docking**

**PDB ID: 4y72**

| 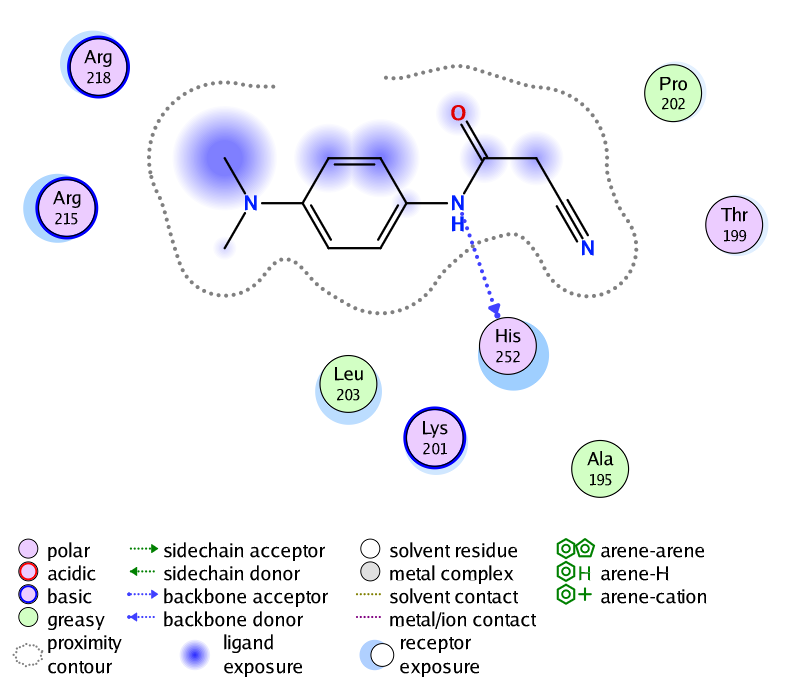 | 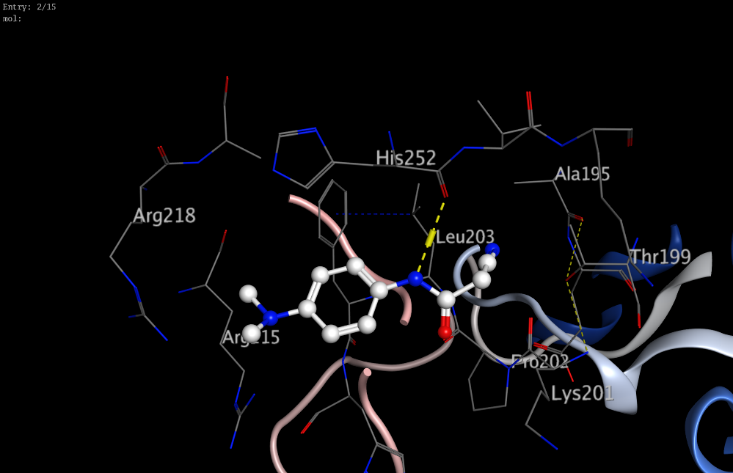 |
| --- | --- |
| **2D** | **3D** |
| **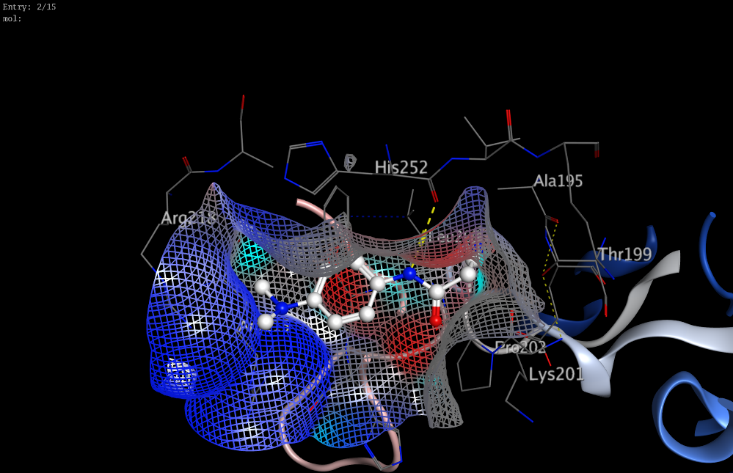** | |
| **Surface map** | |

**Figure S1:-** The binding interaction of **1** with (PDB ID: 4y72).

| 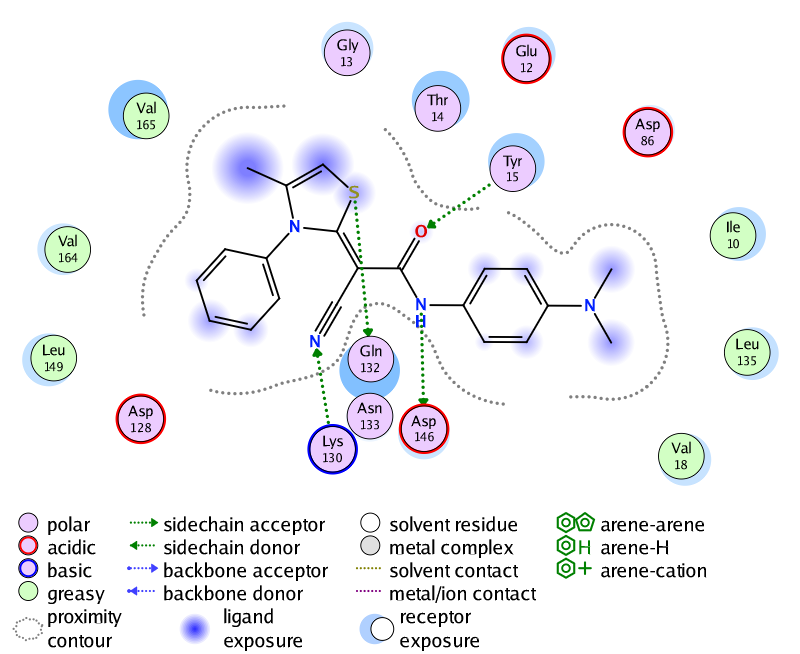 | 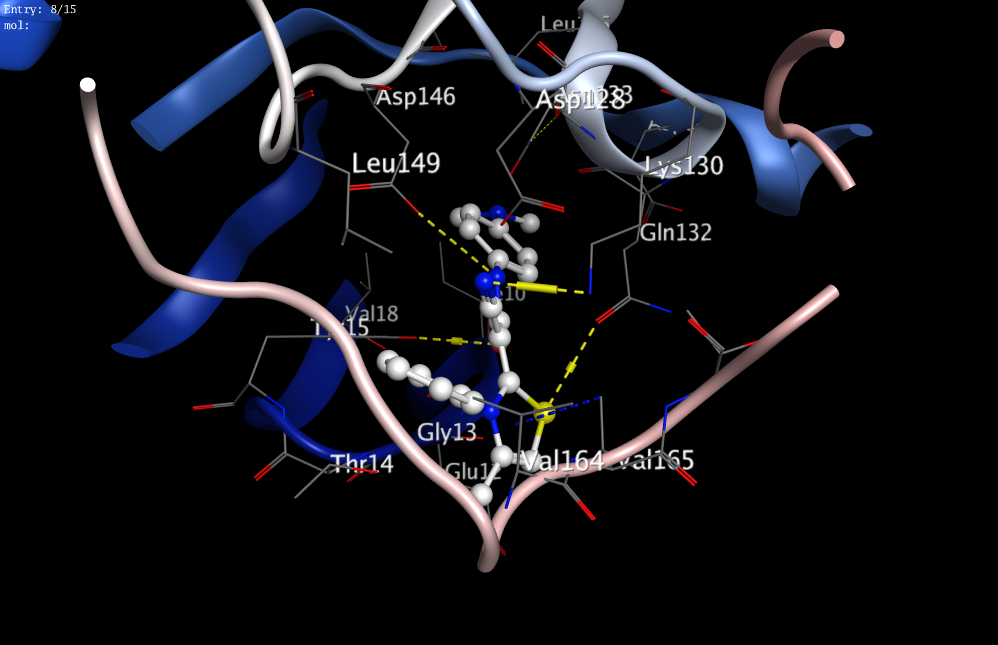 |
| --- | --- |
| **2D** | **3D** |
| 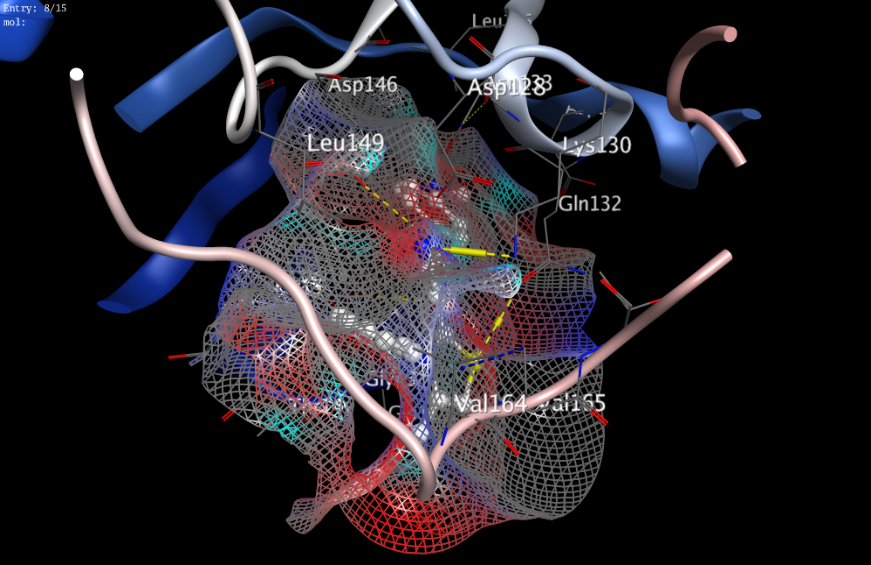 | |
| **Surface map** | |

**Figure S2:-** The binding interaction of **2** with (PDB ID: 4y72).

| 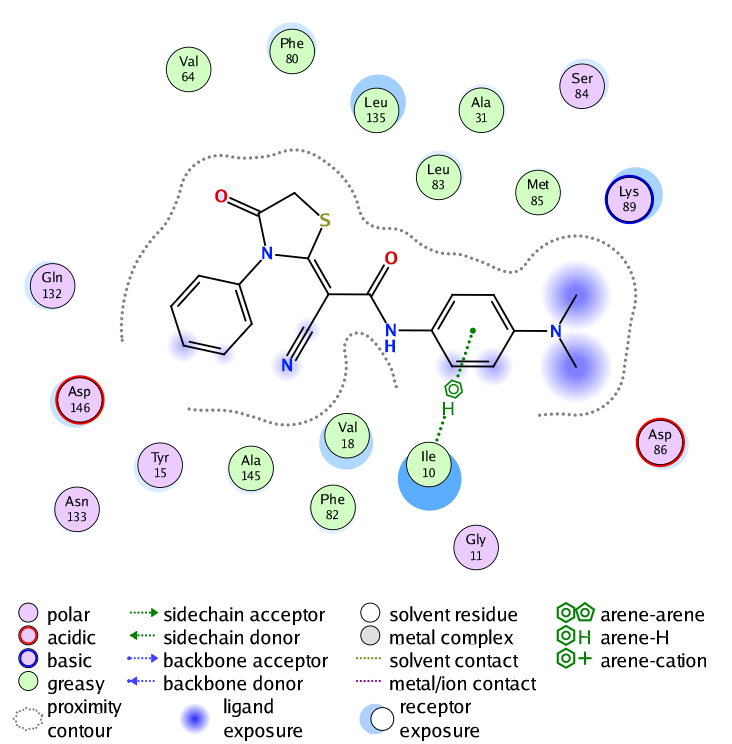 | 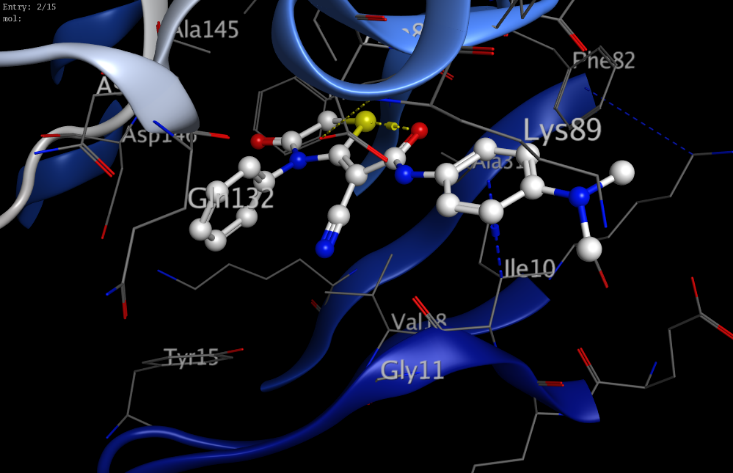 |
| --- | --- |
| **2D** | **3D** |
| **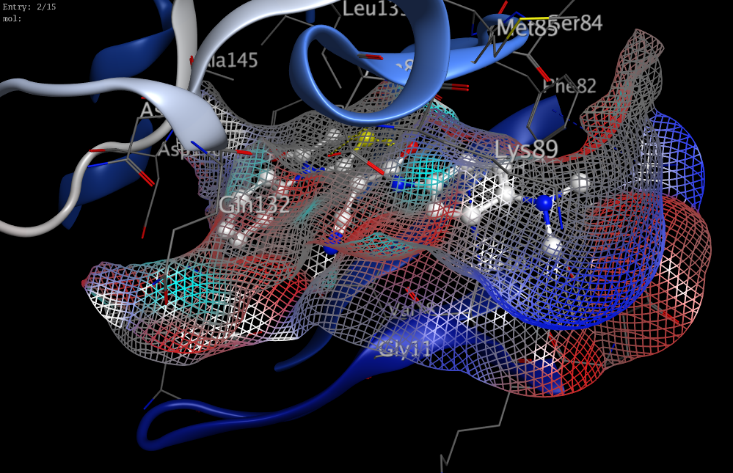** | |
| **Surface map** | |

**Figure S3:-** The binding interaction of **3** with (PDB ID: 4y72)

| 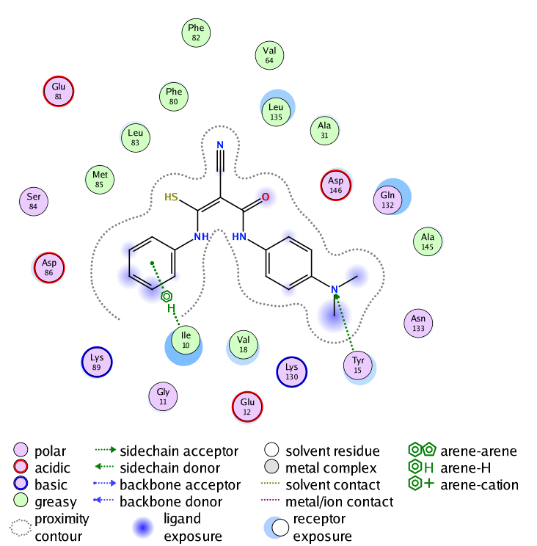 | 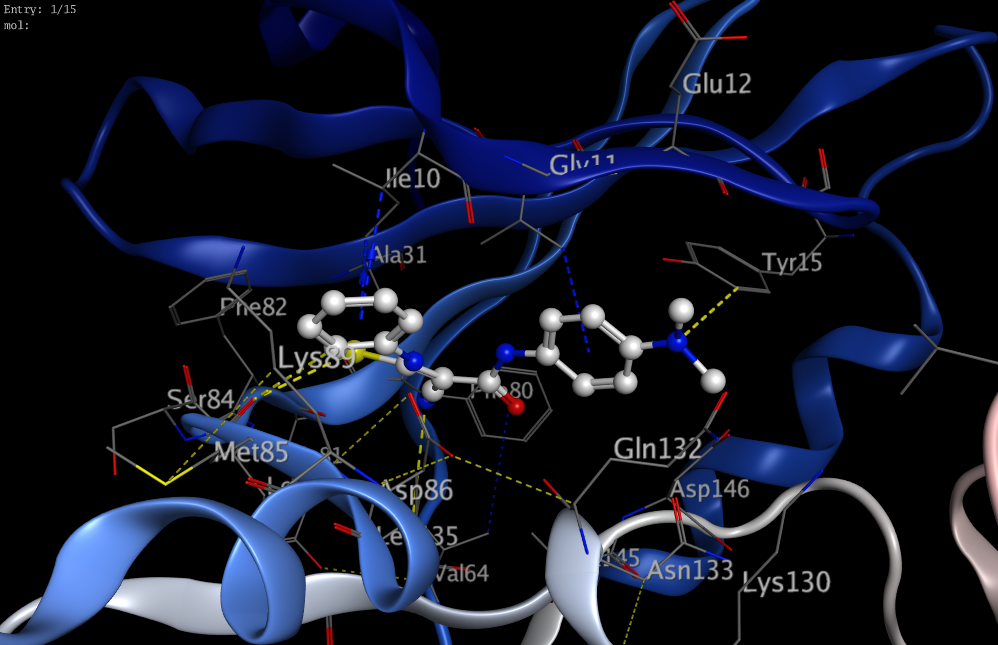 |
| --- | --- |
| **2D** | **3D** |
| **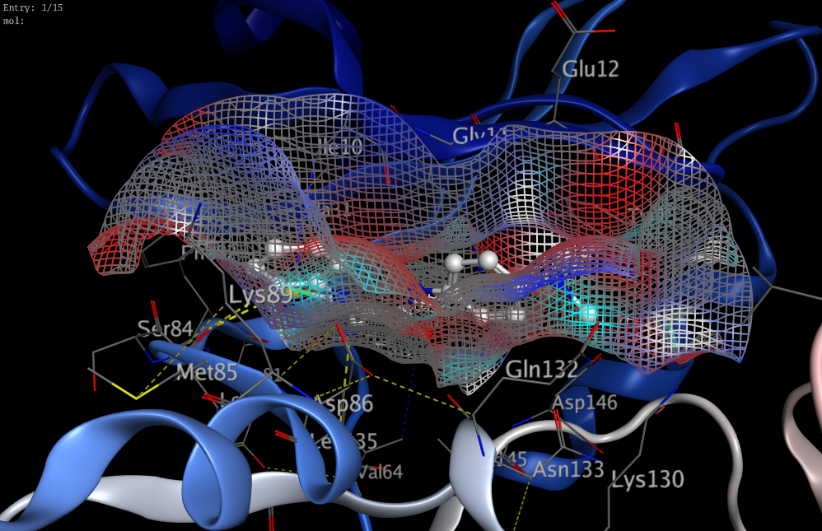** | |
| **Surface map** | |

**Figure S4:-** The binding interaction of **4** with (PDB ID: 4y72).

| 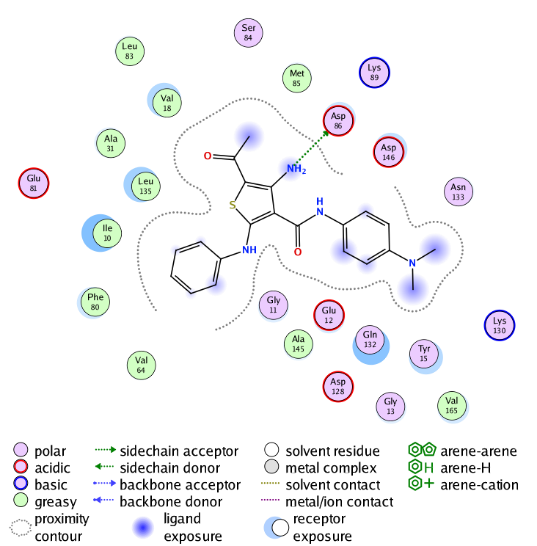 | 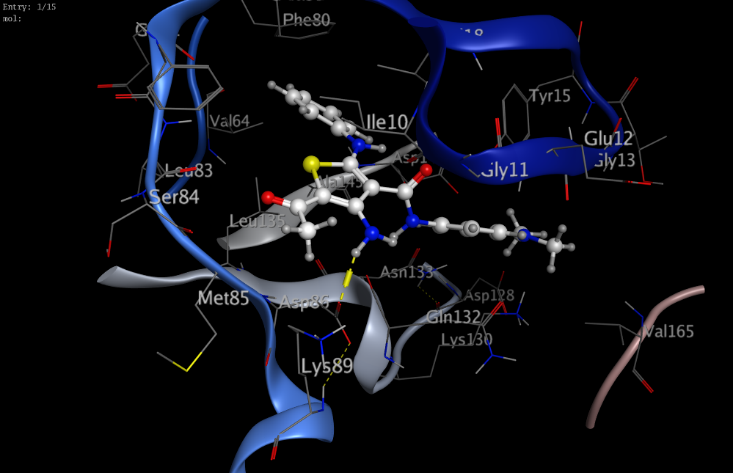 |
| --- | --- |
| **2D** | **3D** |
| **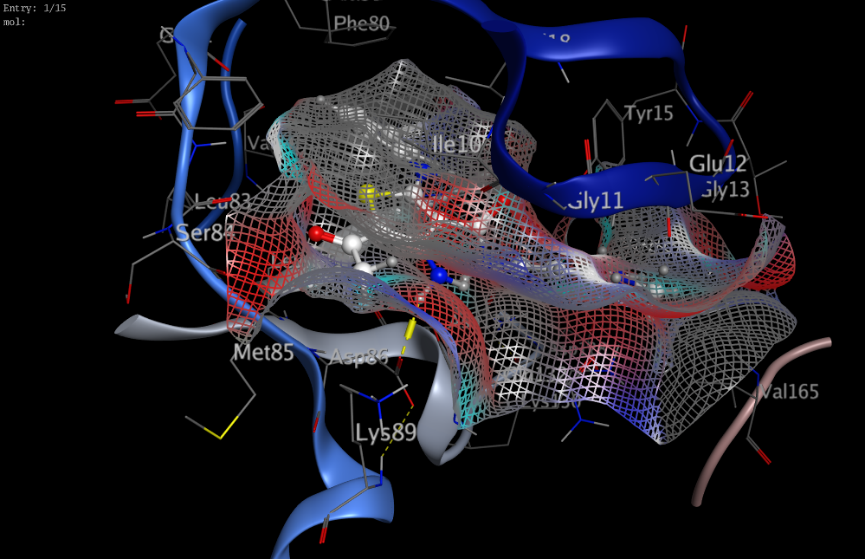** | |
| **Surface map** | |

**Figure S5:-** The binding interaction of **5** with (PDB ID: 4y72).

| 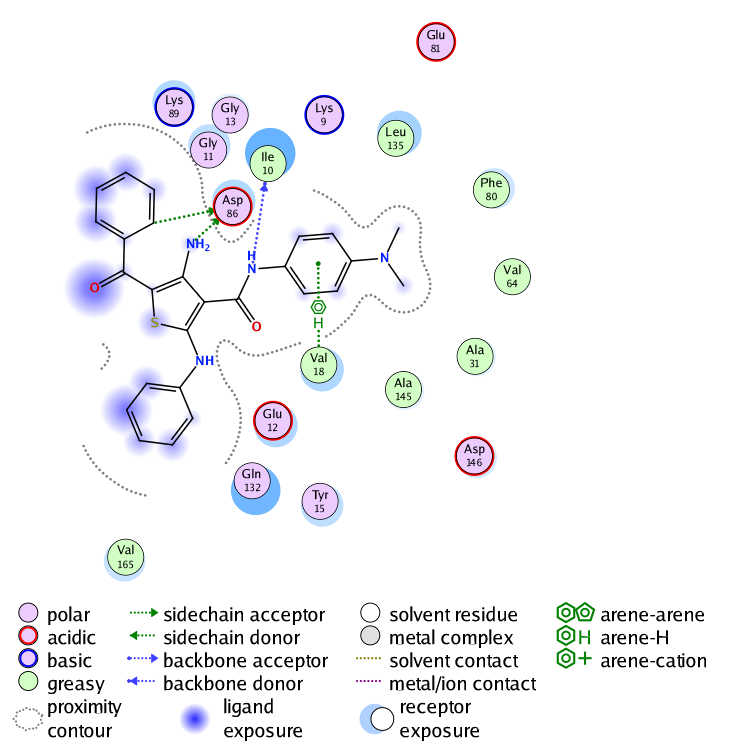 | 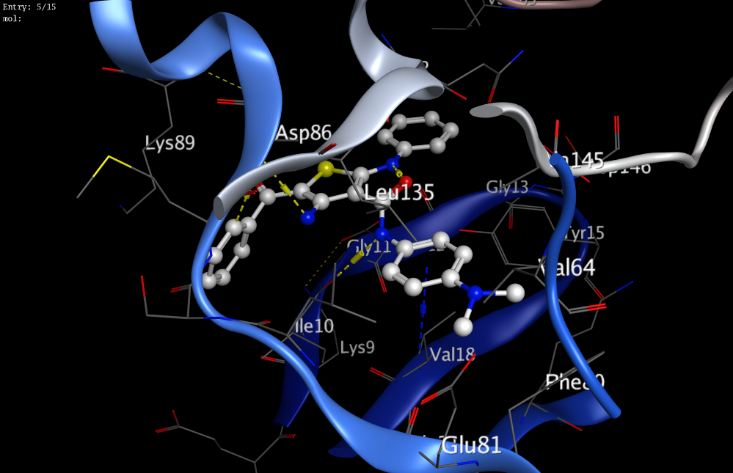 |
| --- | --- |
| **2D** | **3D** |
| 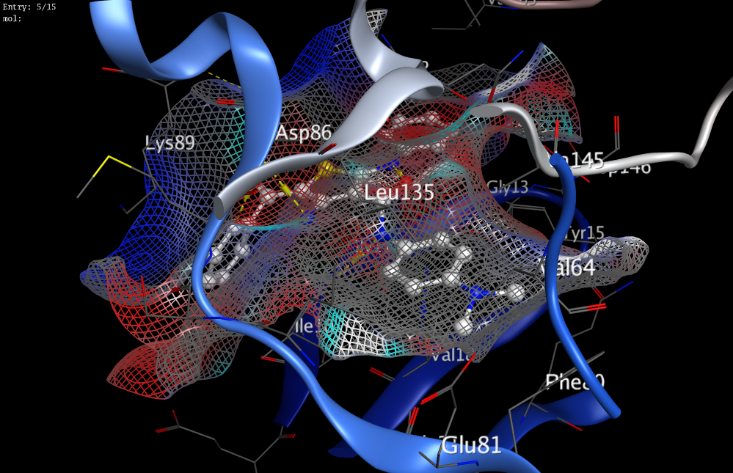 | |
| **Surface map** | |

**Figure S6:-** The binding interaction of **6** with (PDB ID: 4y72).

| 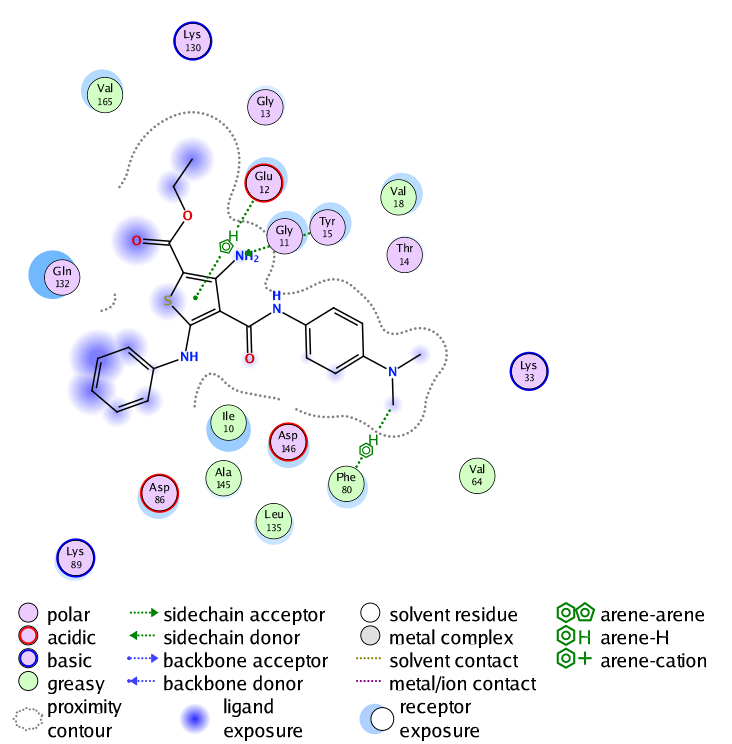 | 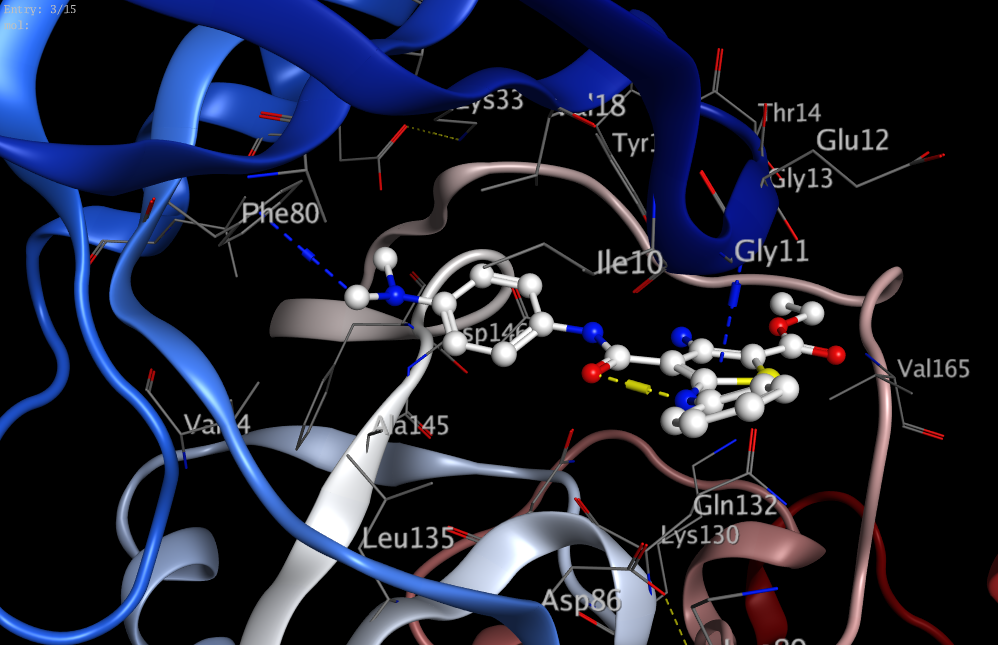 |
| --- | --- |
| **2D** | **3D** |
| **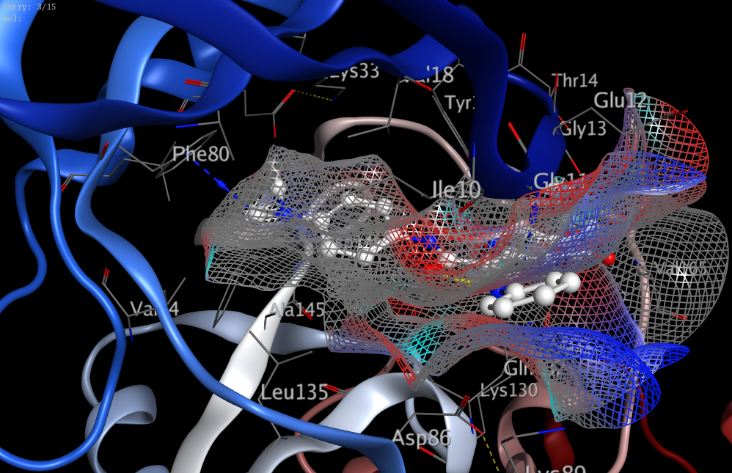** | |
| **Surface map** | |

**Figure S7:-** The binding interaction of **7**with (PDB ID: 4y72).

| 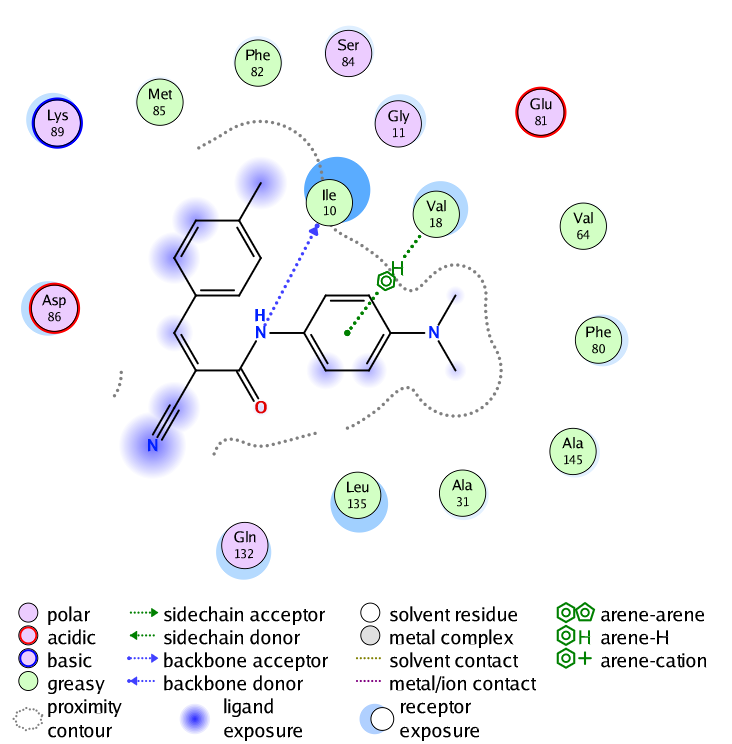 | 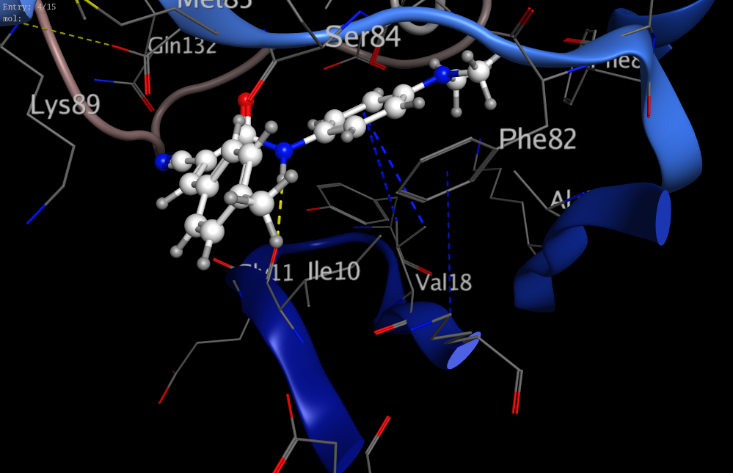 |
| --- | --- |
| **2D** | **3D** |
| **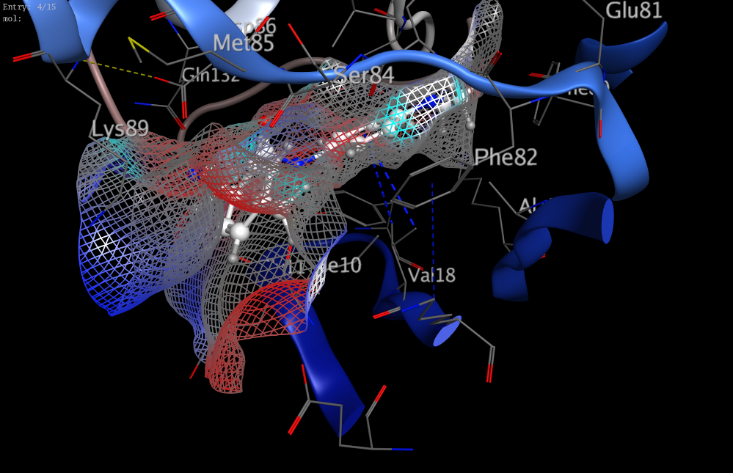** | |
| **Surface map** | |

**Figure S8:-** The binding interaction of **8a**with (PDB ID: 4y72).

| 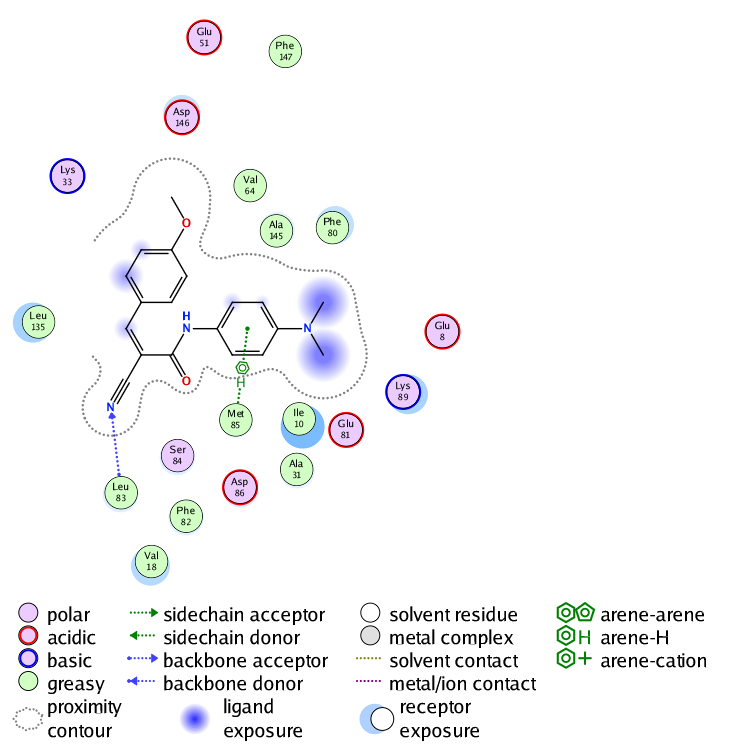 | 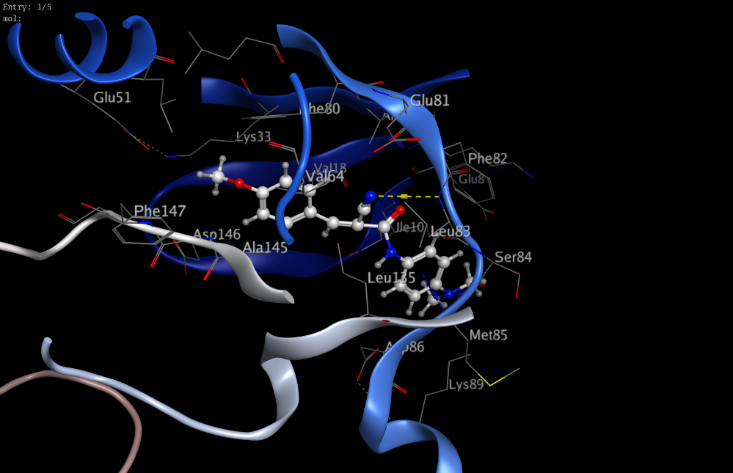 |
| --- | --- |
| **2D** | **3D** |
| **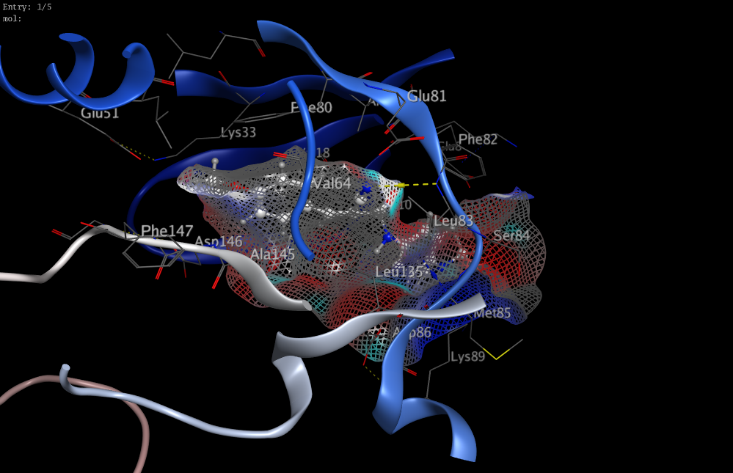** | |
| **Surface map** | |

**Figure S9:-** The binding interaction of **8b** with (PDB ID: 4y72).

| 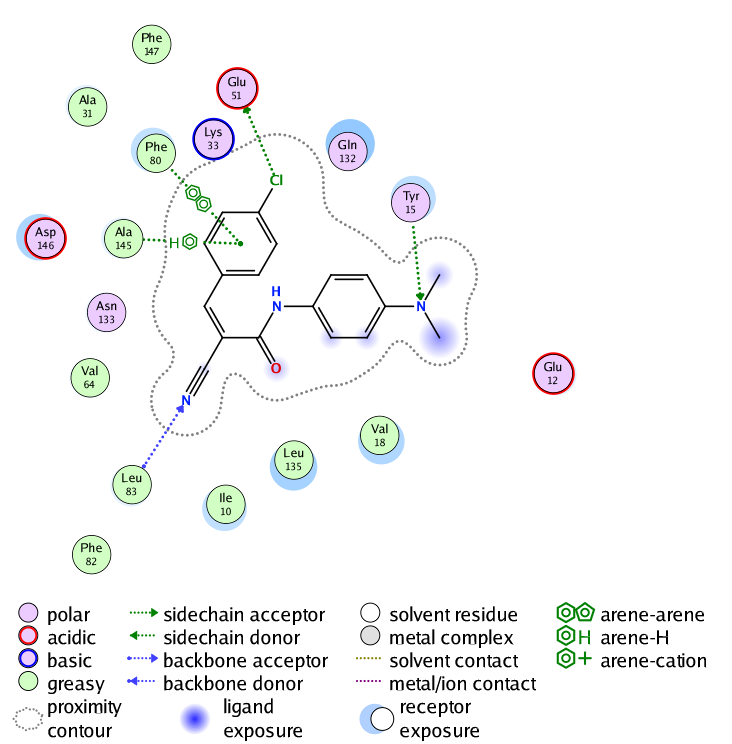 | 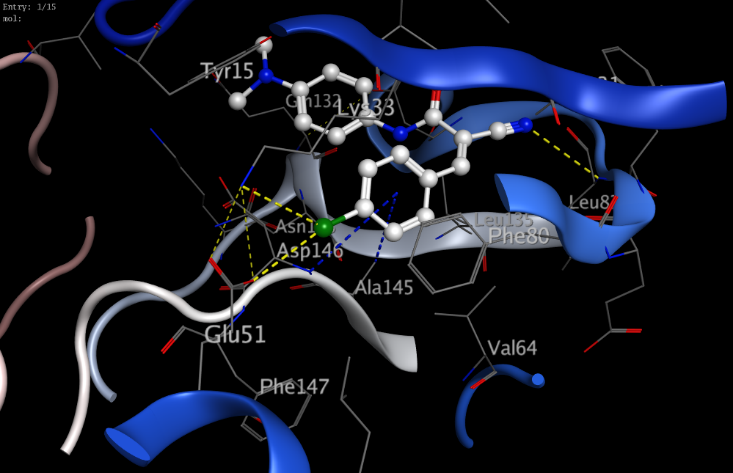 |
| --- | --- |
| **2D** | **3D** |
| **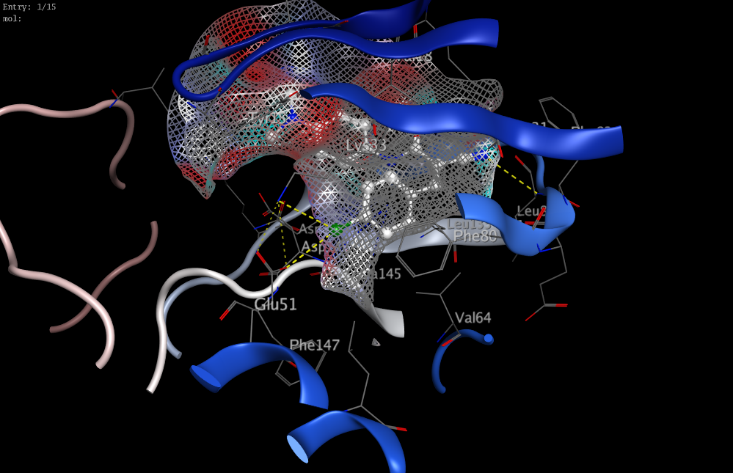** | |
| **Surface map** | |

**Figure S10:-** The binding interaction of **8c** with (PDB ID: 4y72).

| 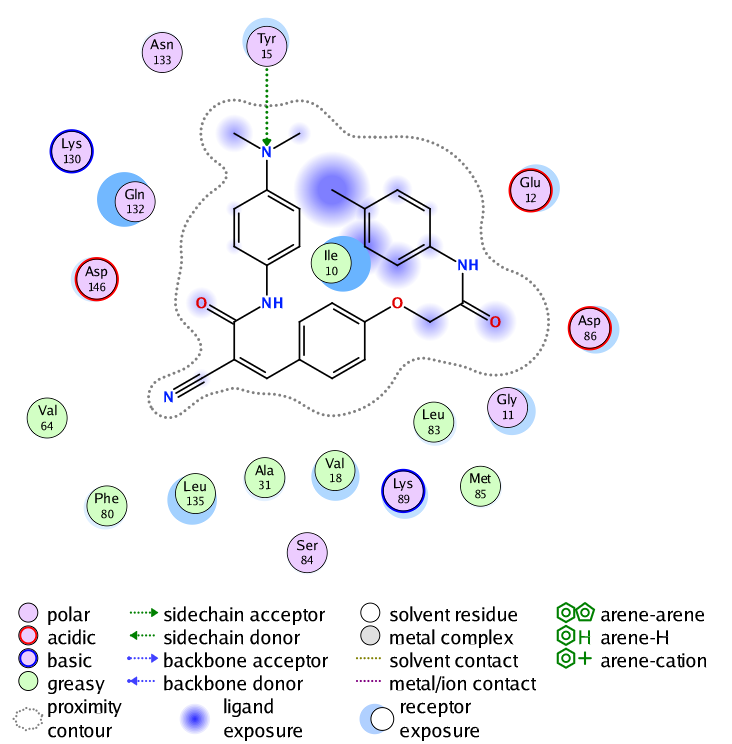 | 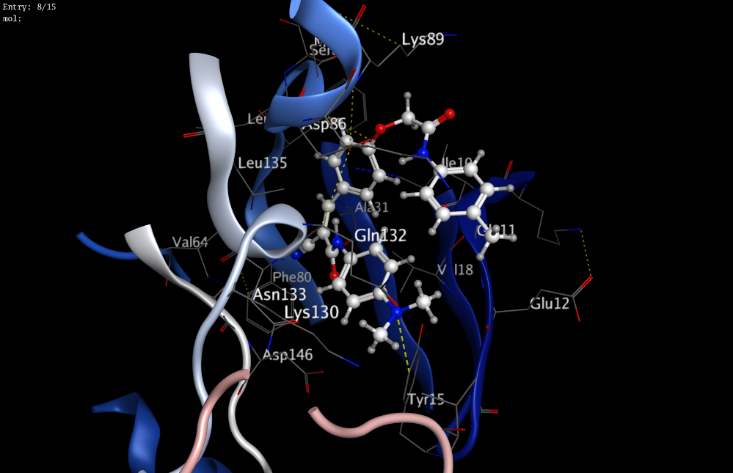 |
| --- | --- |
| **2D** | **3D** |
| 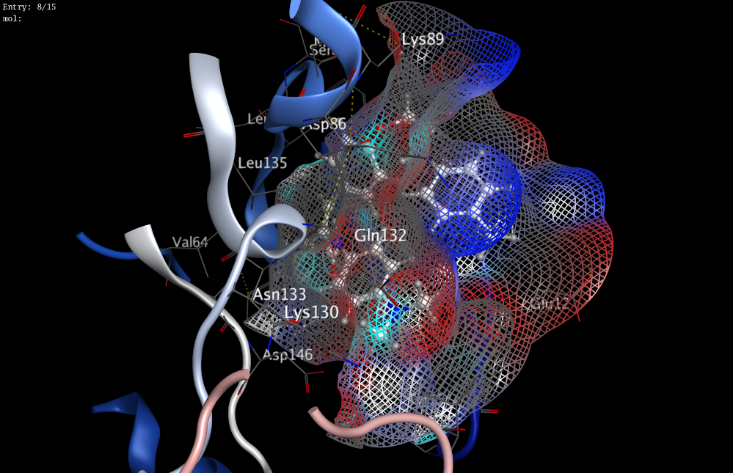 | |
| **Surface map** | |

**Figure S11:-** The binding interaction of **8d** with (PDB ID: 4y72).

| 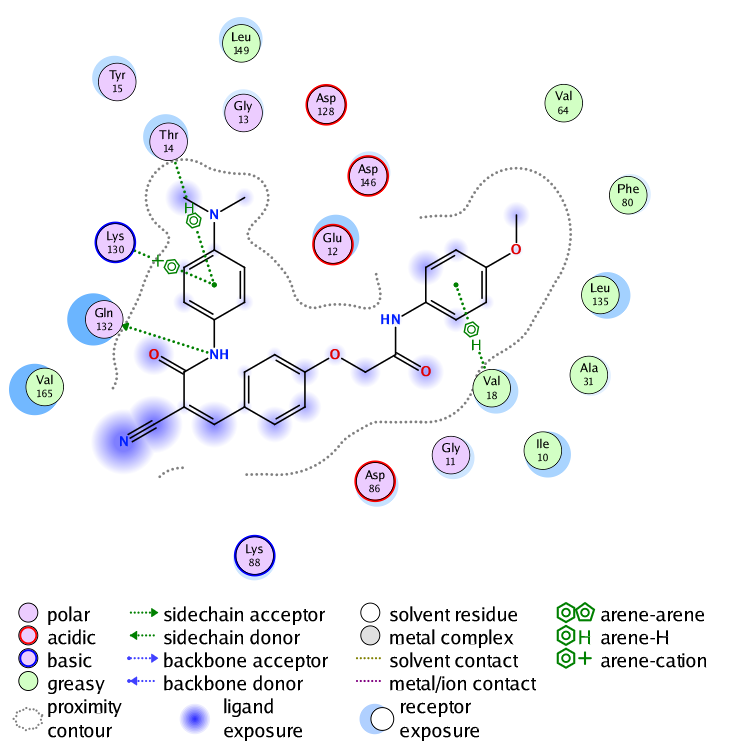 | 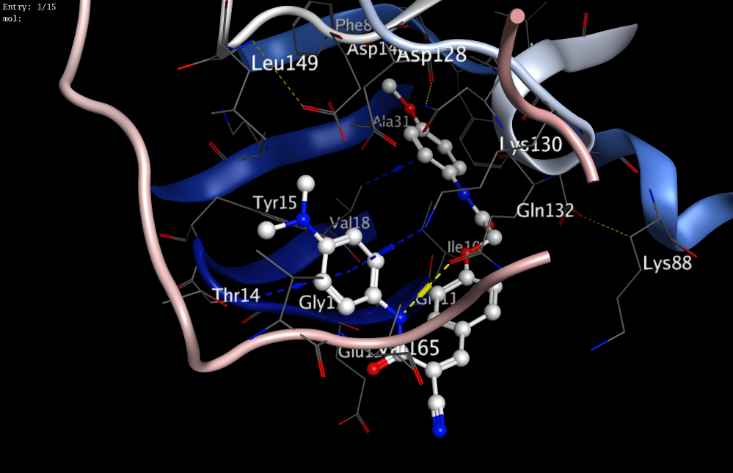 |
| --- | --- |
| **2D** | **3D** |
| 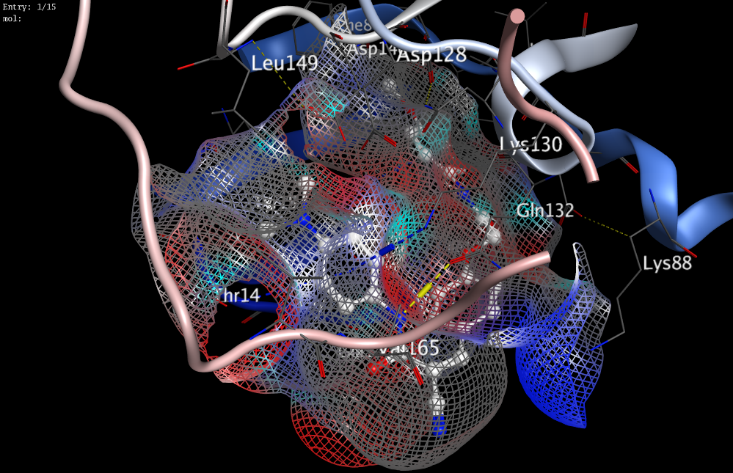 | |
| **Surface map** | |

**Figure S12:-** The binding interaction of **8e** with (PDB ID: 4y72).

| 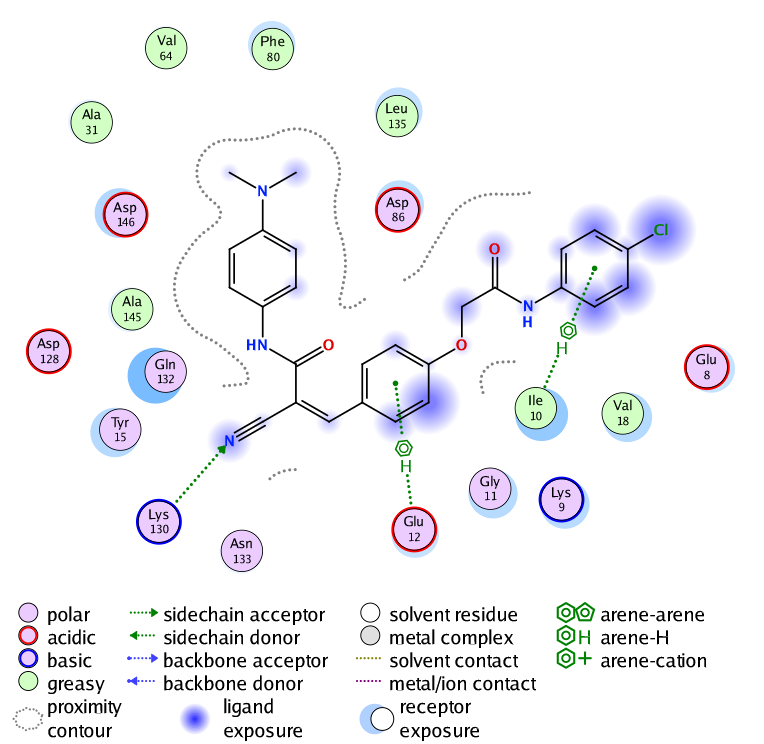 | 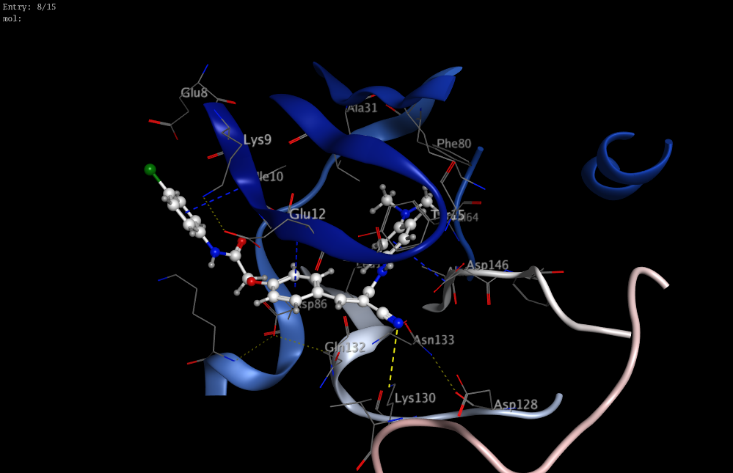 |
| --- | --- |
| **2D** | **3D** |
| 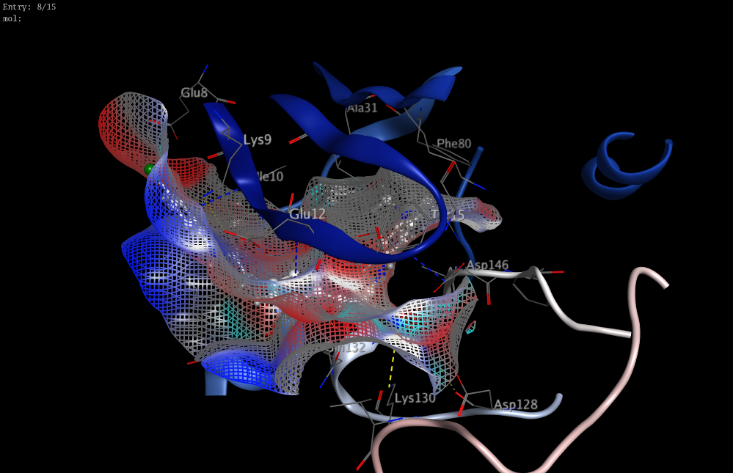 | |
| **Surface map** | |

**Figure S13:-** The binding interaction of **8f** with (PDB ID: 4y72).

| 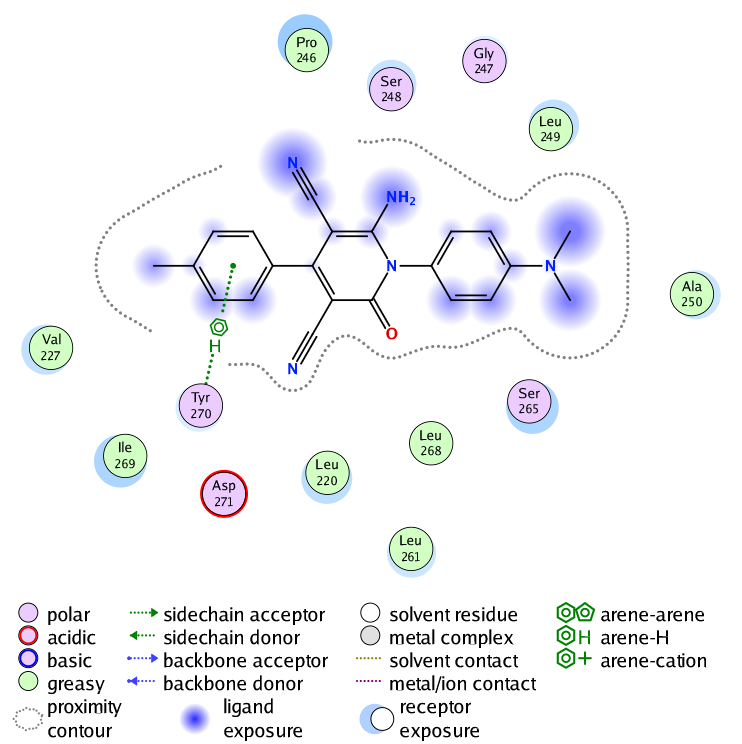 | 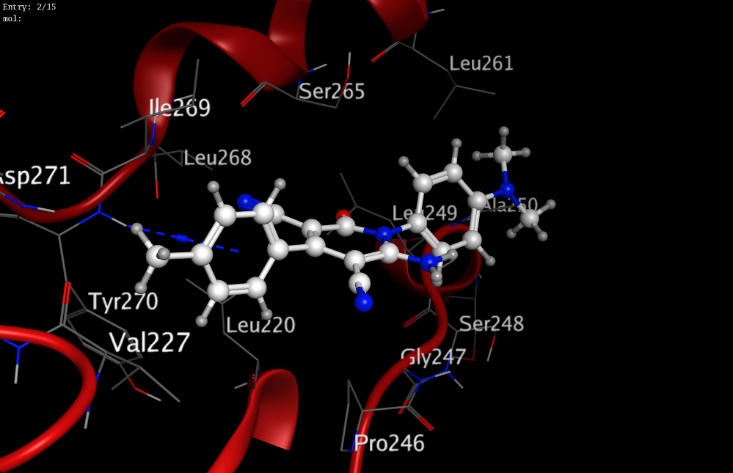 |
| --- | --- |
| **2D** | **3D** |
| 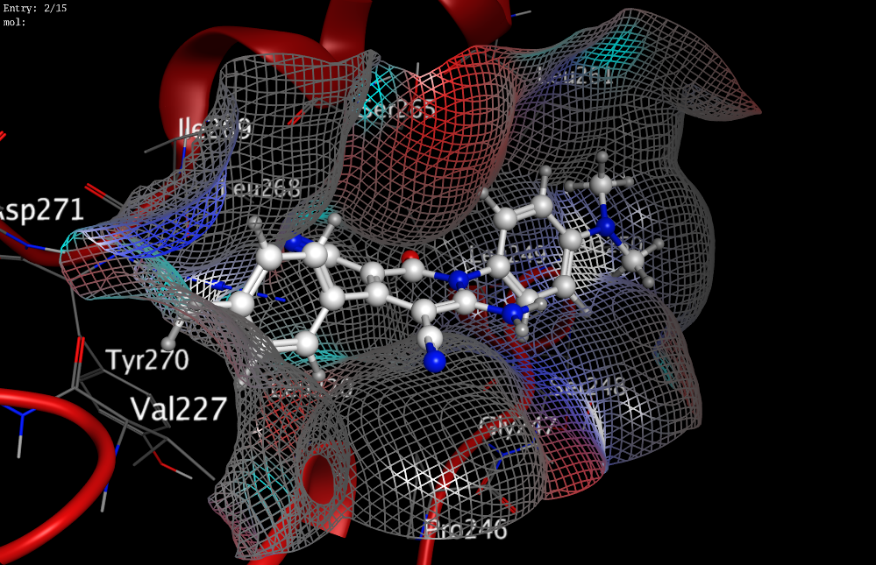 | |
| **Surface map** | |

**Figure S14:-** The binding interaction of **9a**with (PDB ID: 4y72).

| 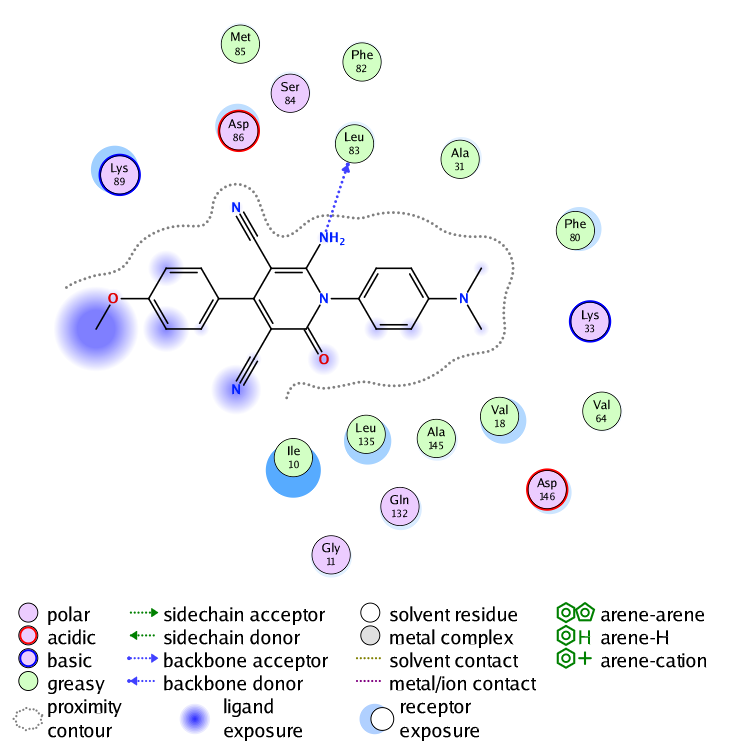 | 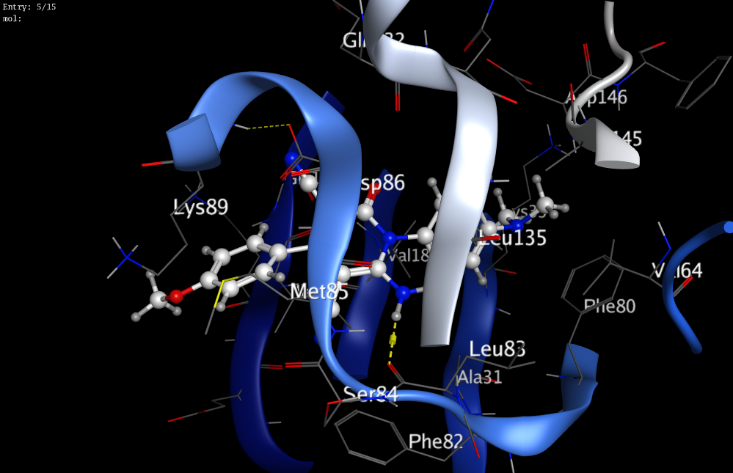 |
| --- | --- |
| **2D** | **3D** |
| 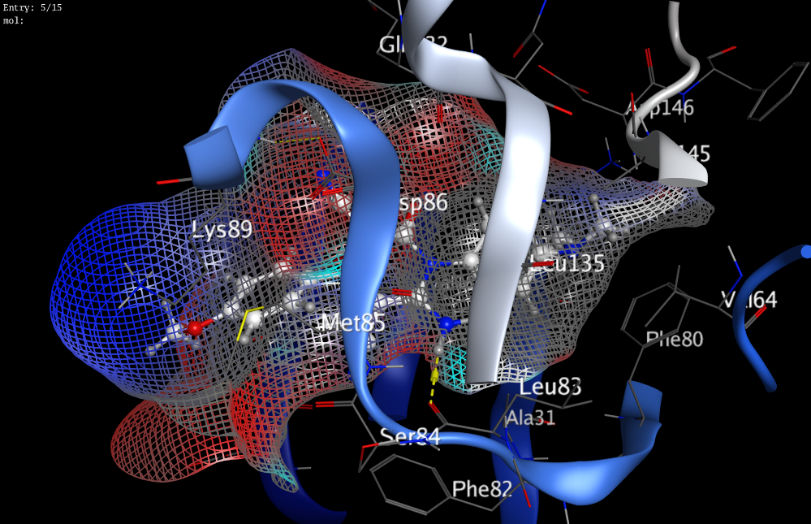 | |
| **Surface map** | |

**Figure S15:-** The binding interaction of **9b** with (PDB ID: 4y72).

| 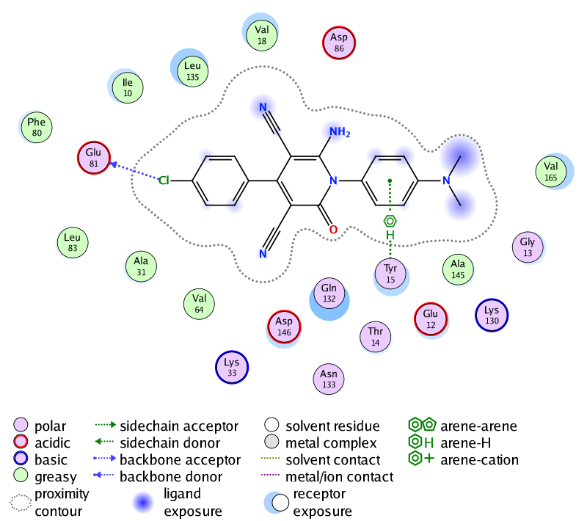 | 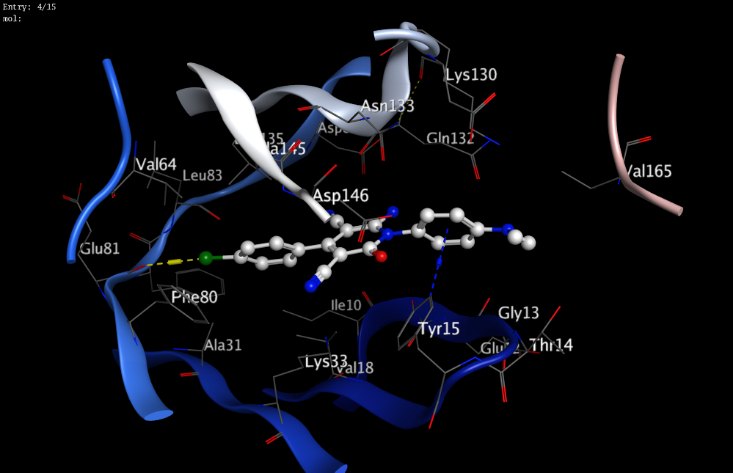 |
| --- | --- |
| **2D** | **3D** |
| **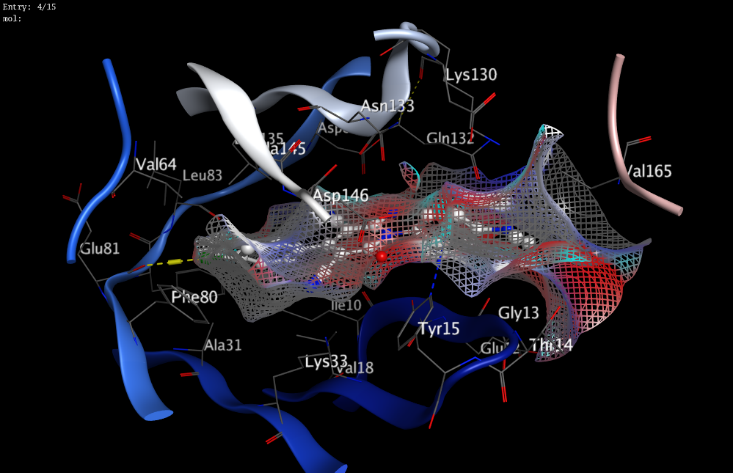** | |
| **Surface map** | |

**Figure S16:-** The binding interaction of **9c** with (PDB ID: 4y72).

| 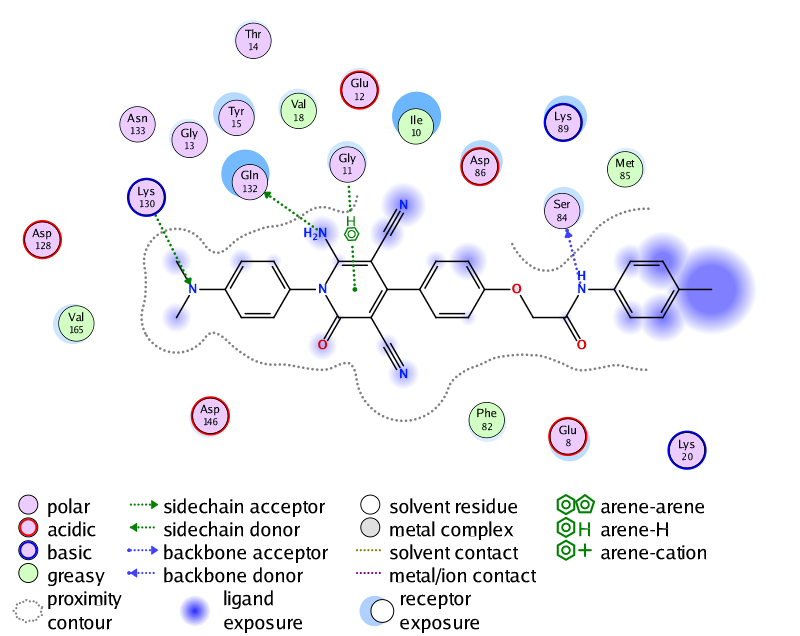 | 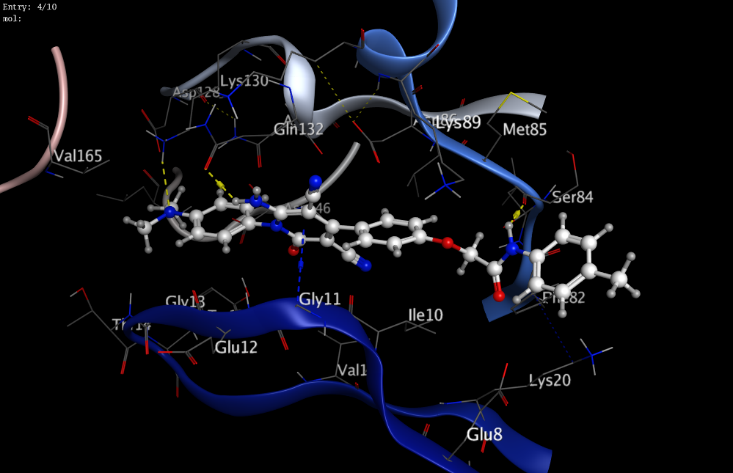 |
| --- | --- |
| **2D** | **3D** |
| 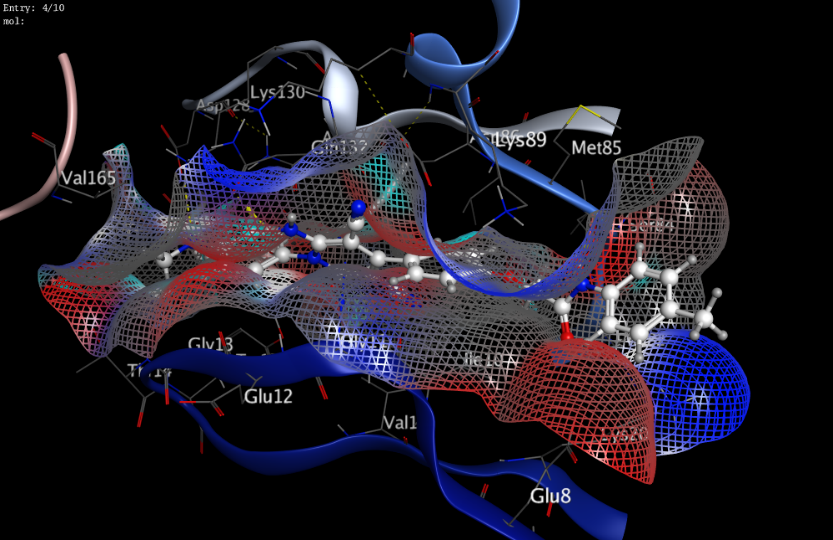 | |
| **Surface map** | |

**Figure S17:-** The binding interaction of **9d** with (PDB ID: 4y72).

| 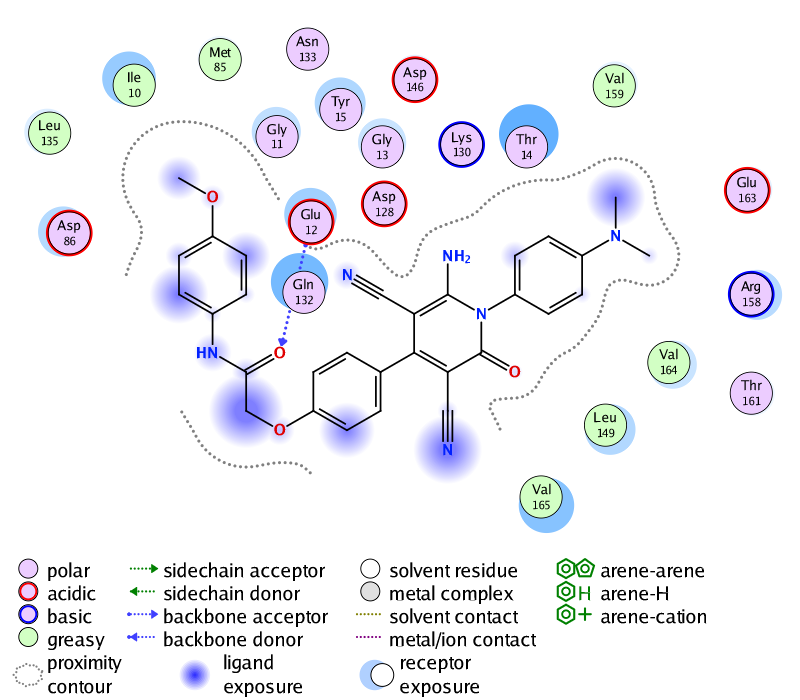 | 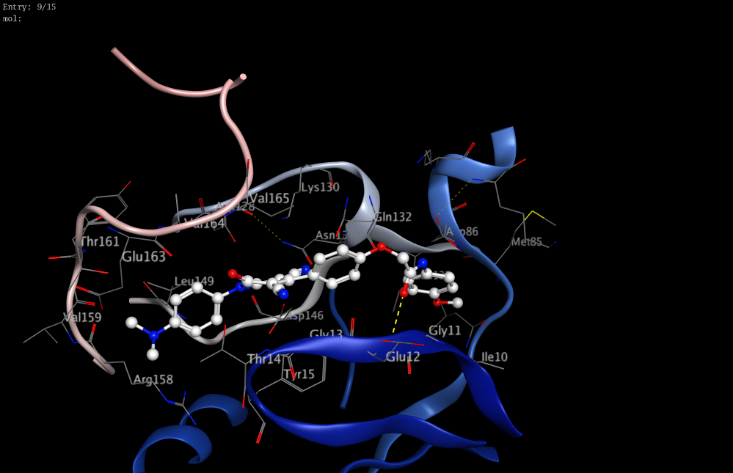 |
| --- | --- |
| **2D** | **3D** |
| 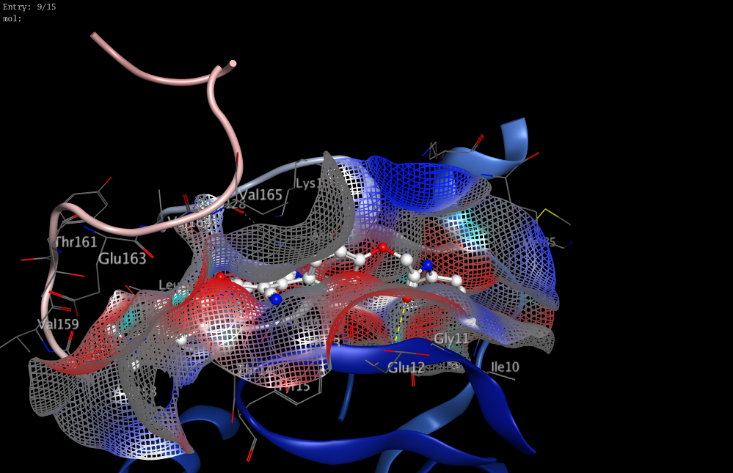 | |
| **Surface map** | |

**Figure S18:-** The binding interaction of **9e** with (PDB ID: 4y72).

| 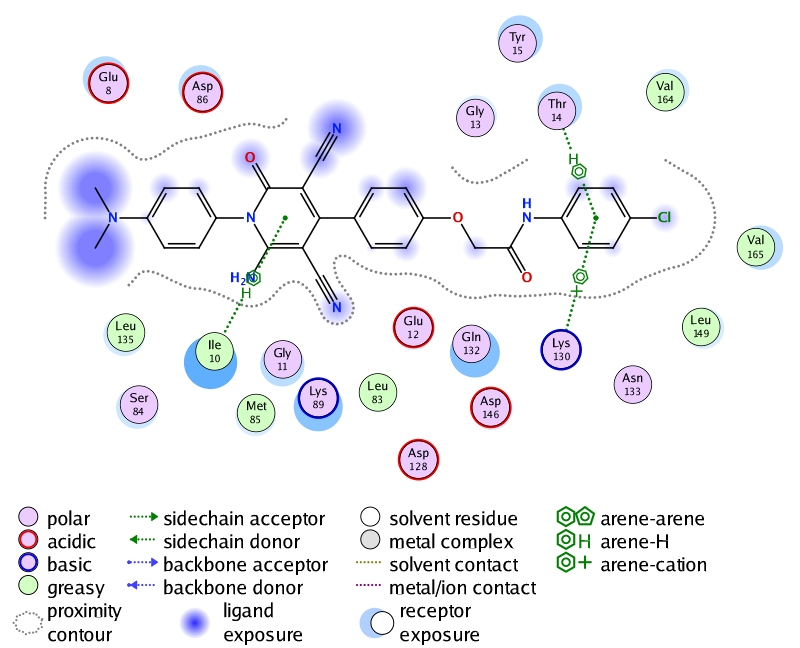 | 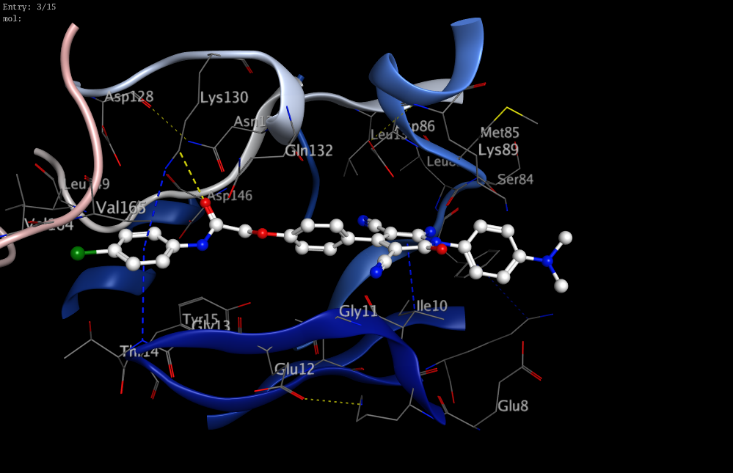 |
| --- | --- |
| **2D** | **3D** |
| 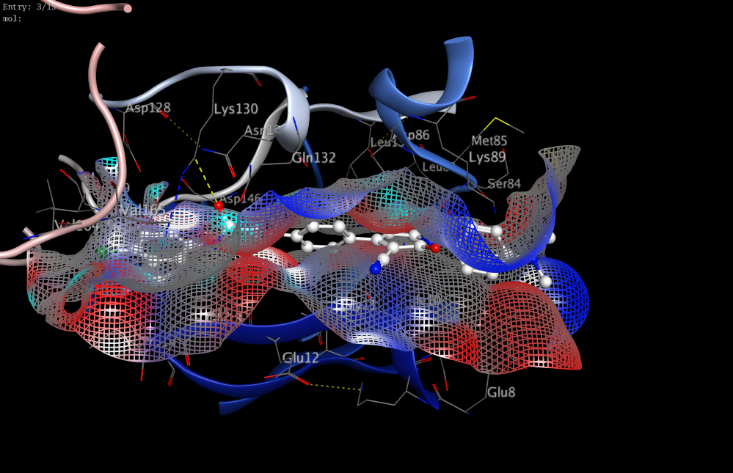 | |
| **Surface map** | |

**Figure S19:-** The binding interaction of **9f** with (PDB ID: 4y72).

| 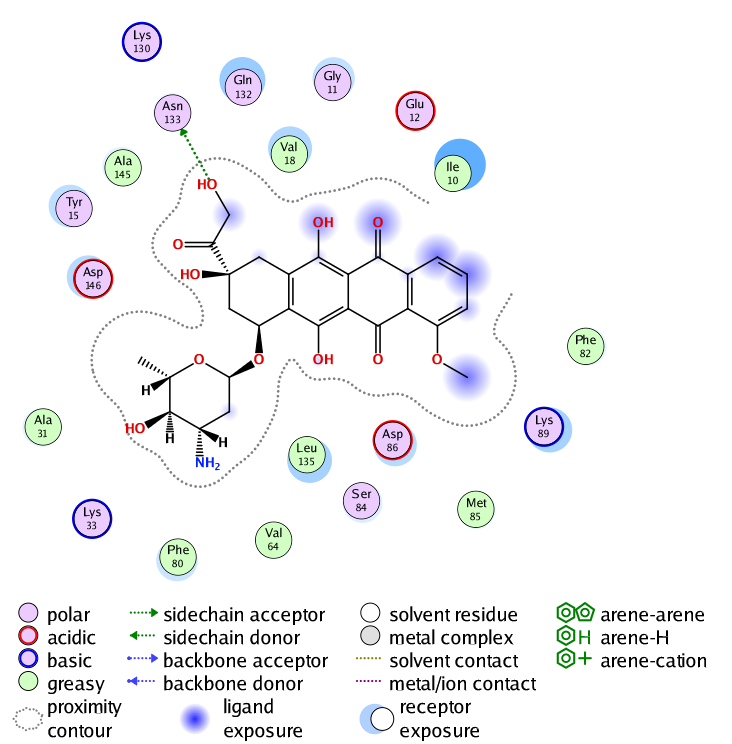 | 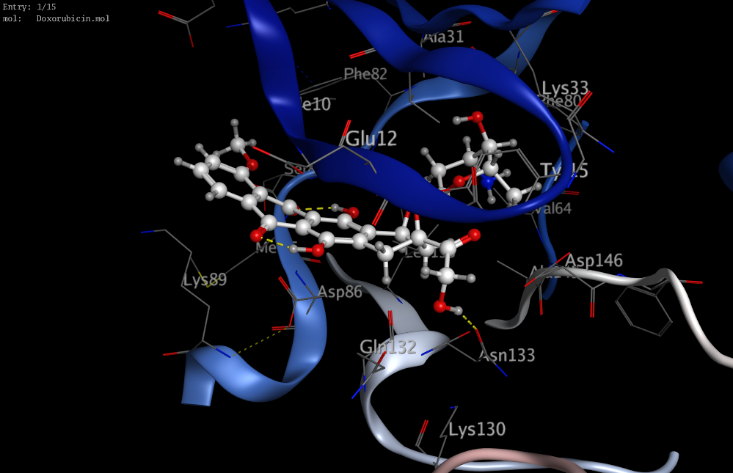 |
| --- | --- |
| **2D** | **3D** |
| 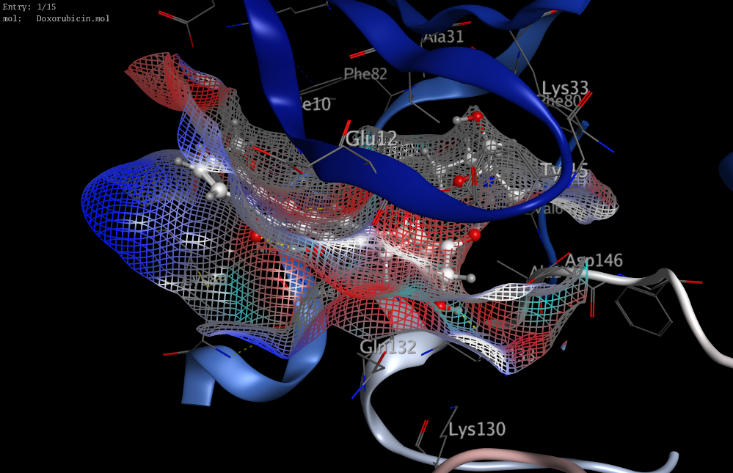 | |
| **Surface map** | |

**Figure S20:-** The binding interaction Doxorubicin with (PDB ID: 4y72).

**Molecular docking**

**PDB ID: 2ra3**

| 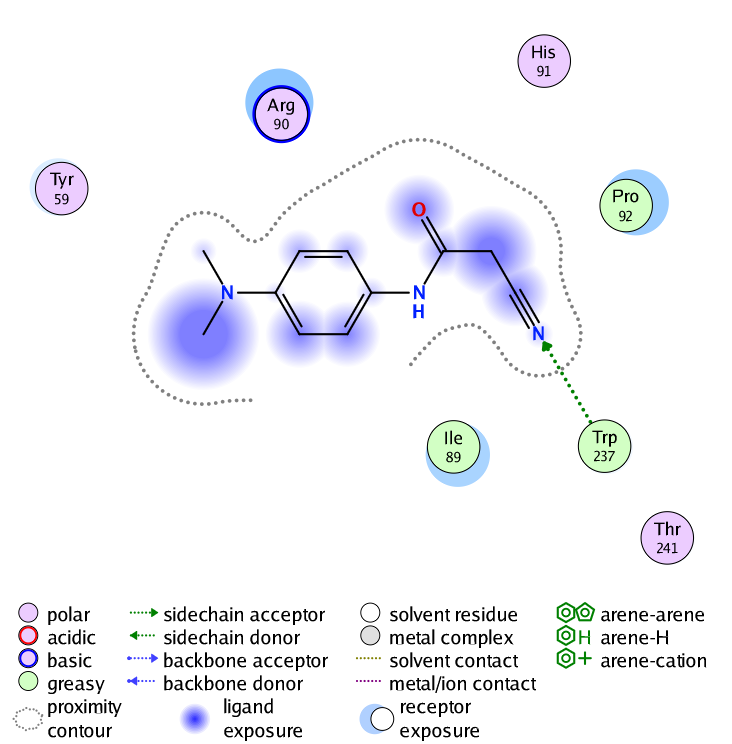 | 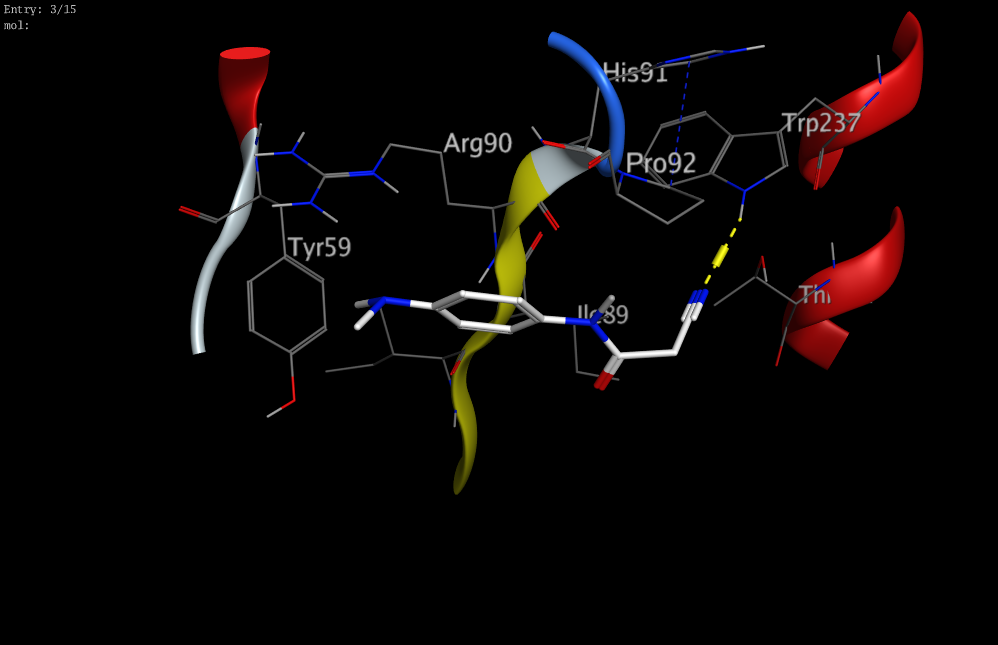 |
| --- | --- |
| **2D** | **3D** |
| **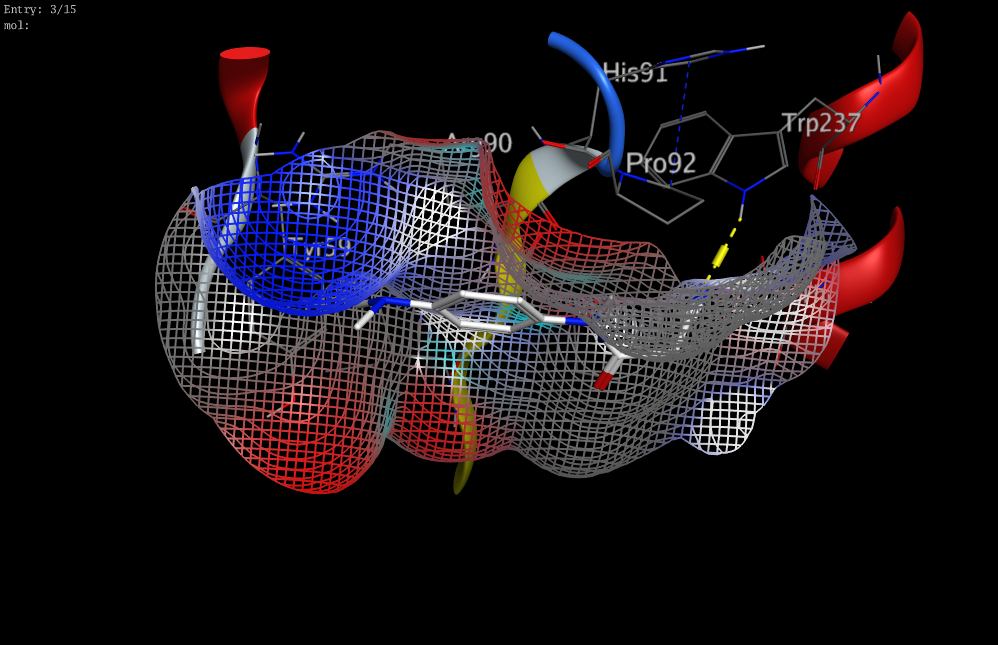** | |
| **Surface map** | |

**Figure S21:-** The binding interaction of **1** with (PDB ID: **2ra3**).

| 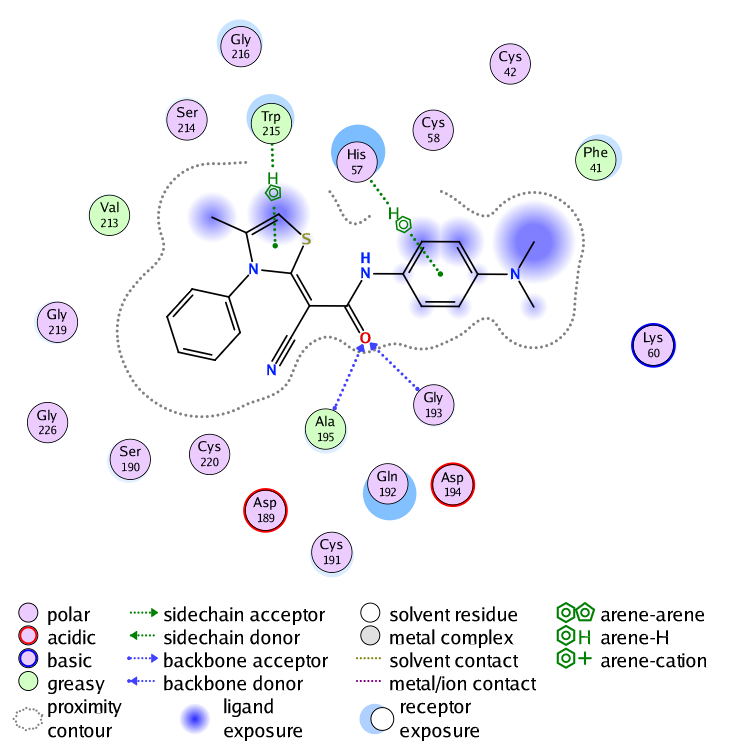 | 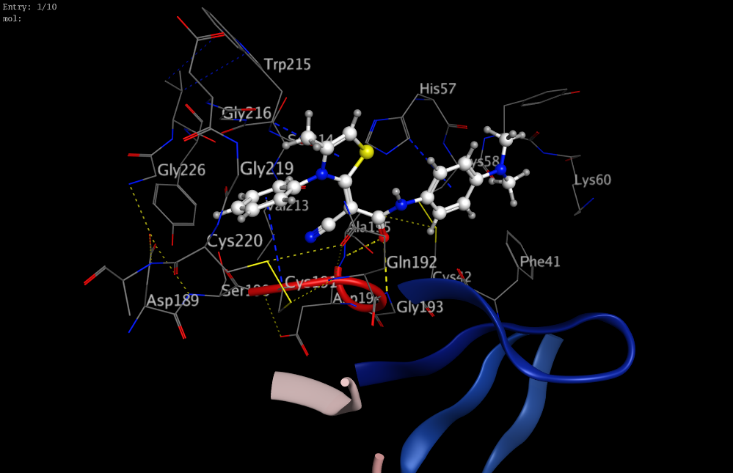 |
| --- | --- |
| **2D** | **3D** |
| **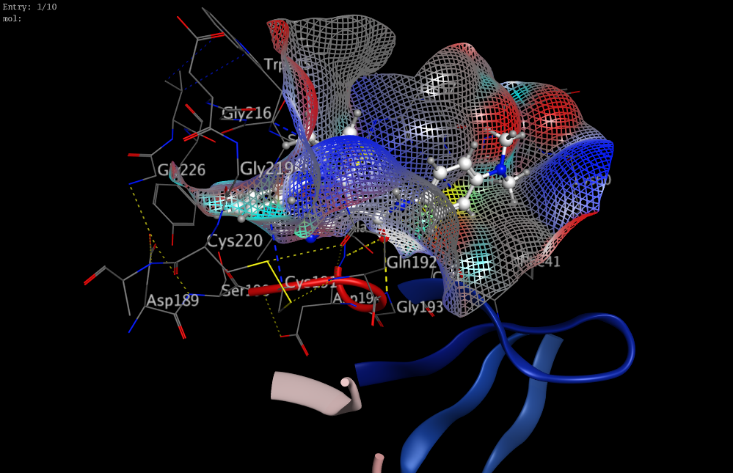** | |
| **Surface map** | |

**Figure S22:-** The binding interaction of **2** with (PDB ID: **2ra3**).

| 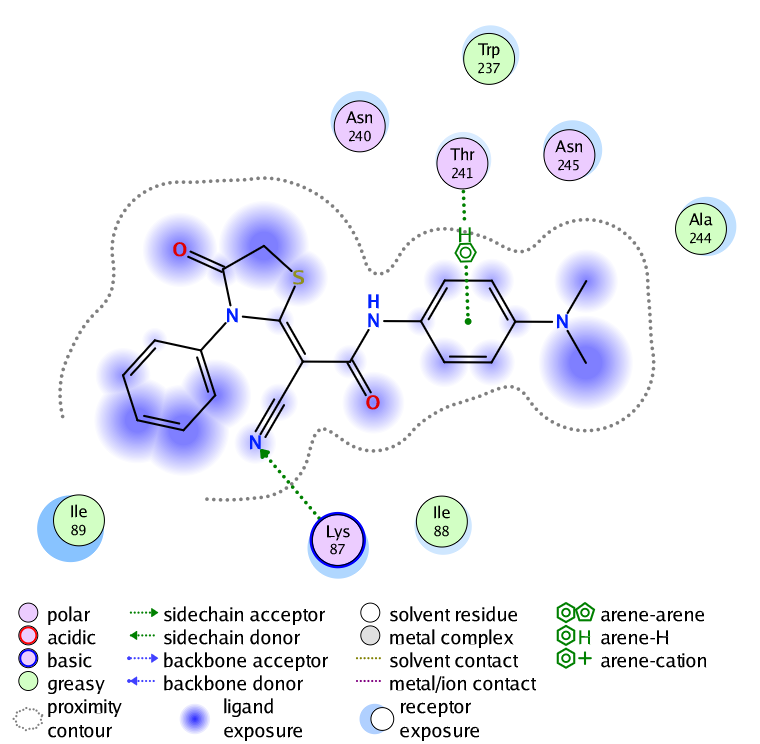 | 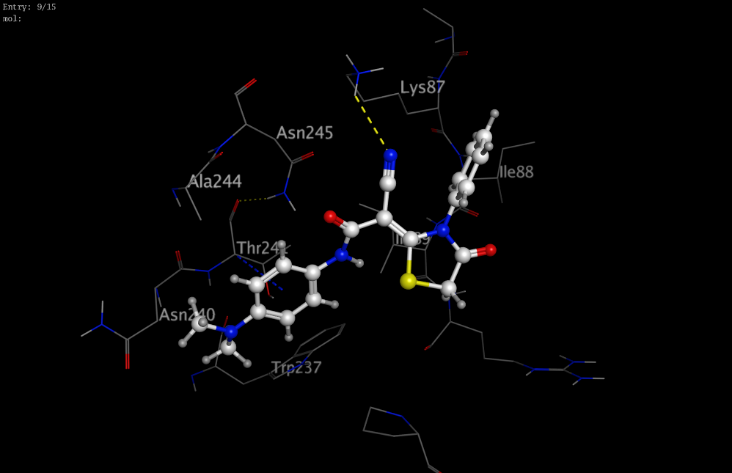 |
| --- | --- |
| **2D** | **3D** |
| **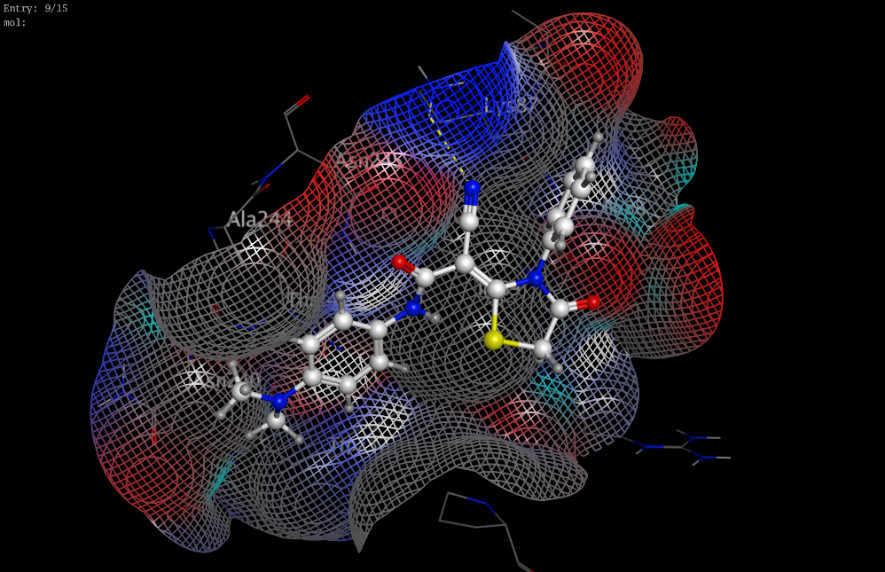** | |
| **Surface map** | |

**Figure S23:-** The binding interaction of **3** with (PDB ID: **2ra3**)

| 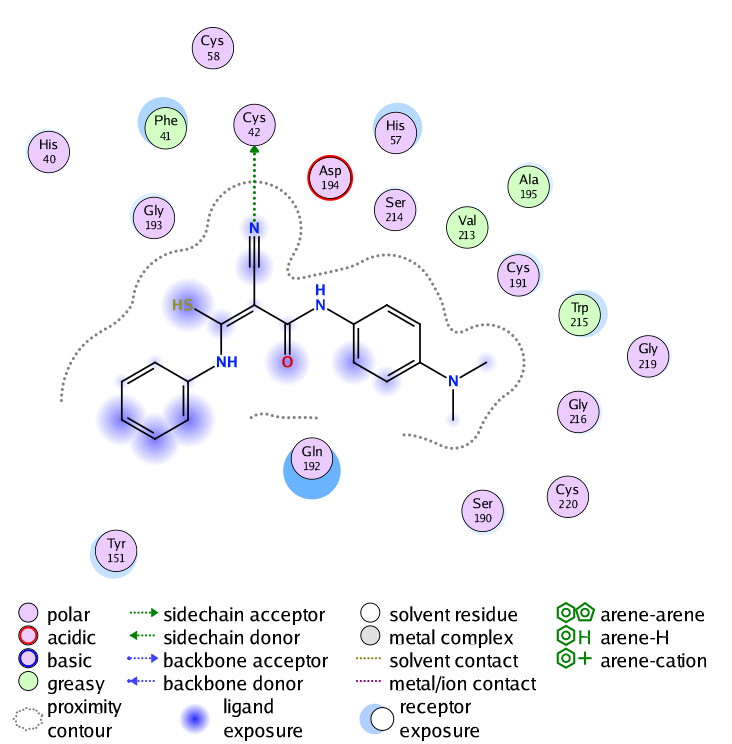 | 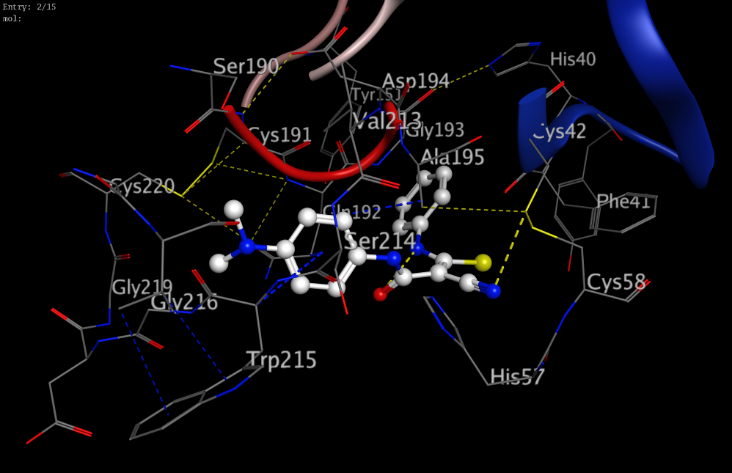 |
| --- | --- |
| **2D** | **3D** |
| **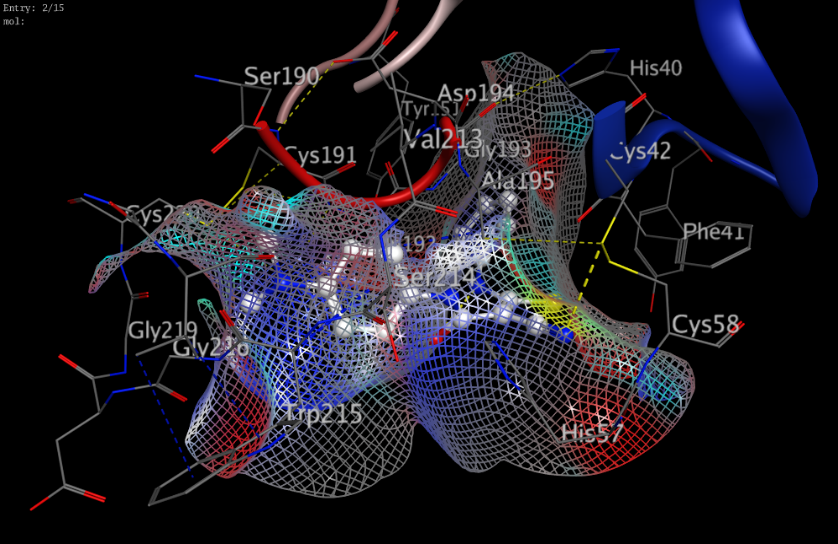** | |
| **Surface map** | |

**Figure S24:-** The binding interaction of **4** with (PDB ID: **2ra3**).

| 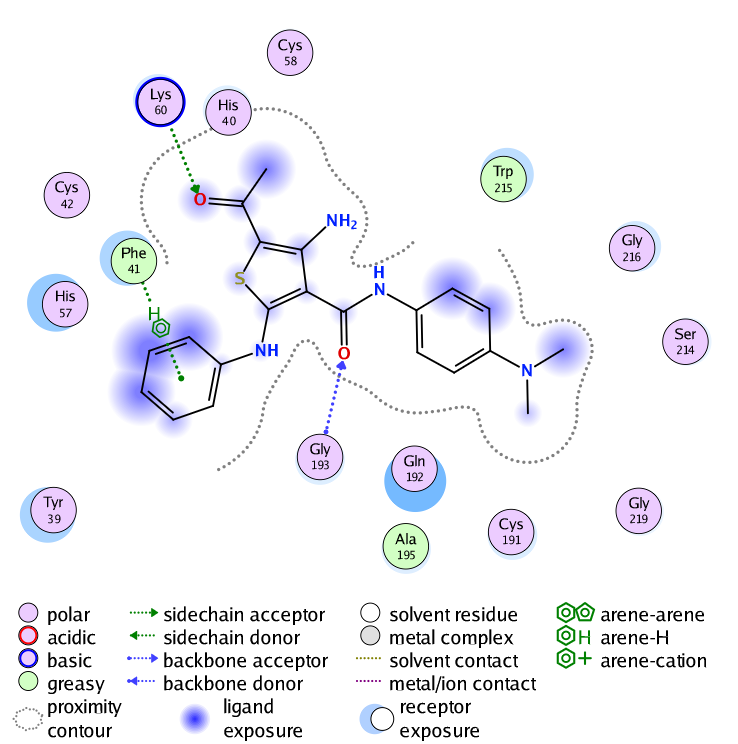 | 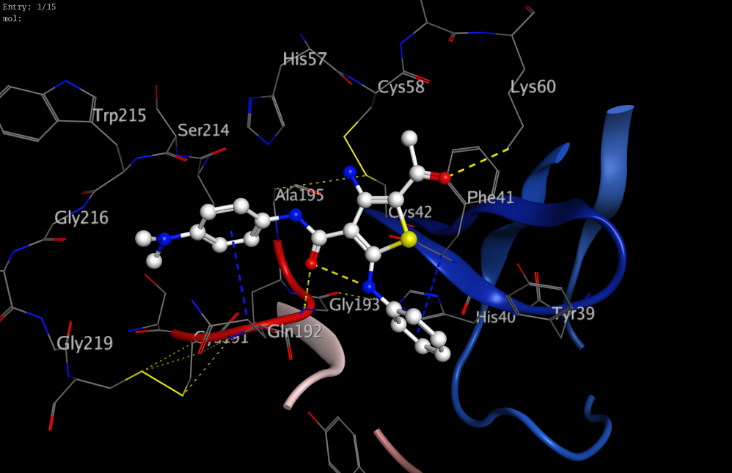 |
| --- | --- |
| **2D** | **3D** |
| **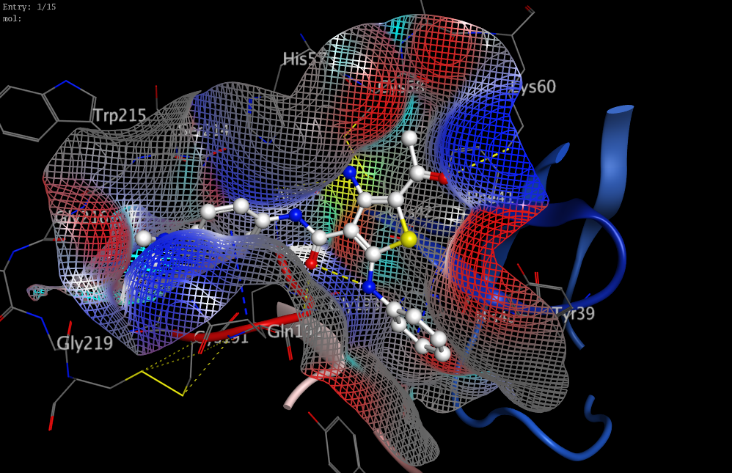** | |
| **Surface map** | |

**Figure S25:-** The binding interaction of **5** with (PDB ID: **2ra3**).

| 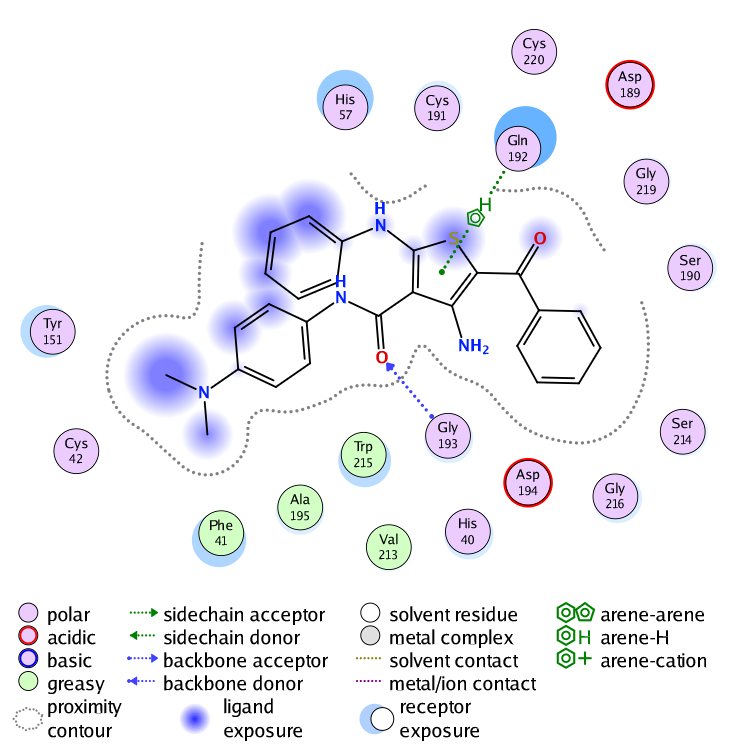 | 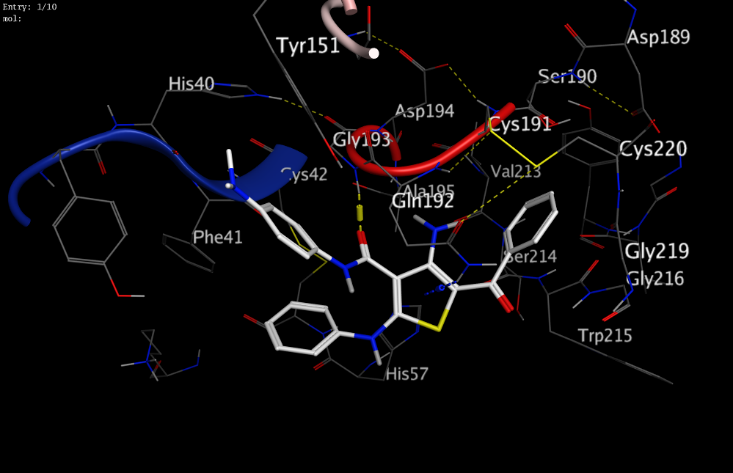 |
| --- | --- |
| **2D** | **3D** |
| 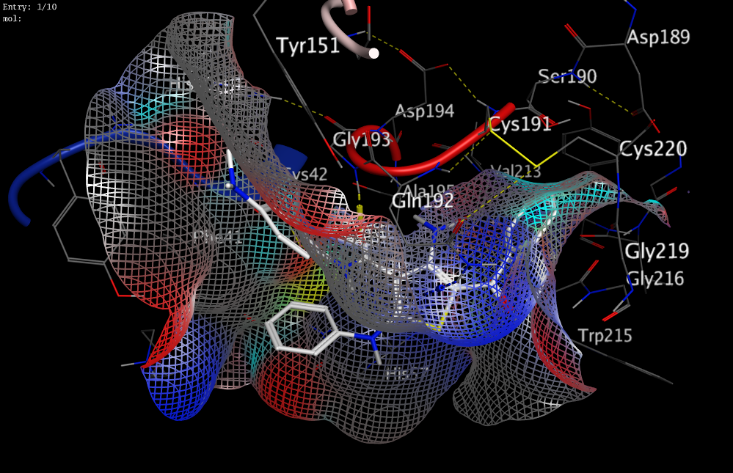 | |
| **Surface map** | |

**Figure S26:-** The binding interaction of **6** with (PDB ID: **2ra3**).

| 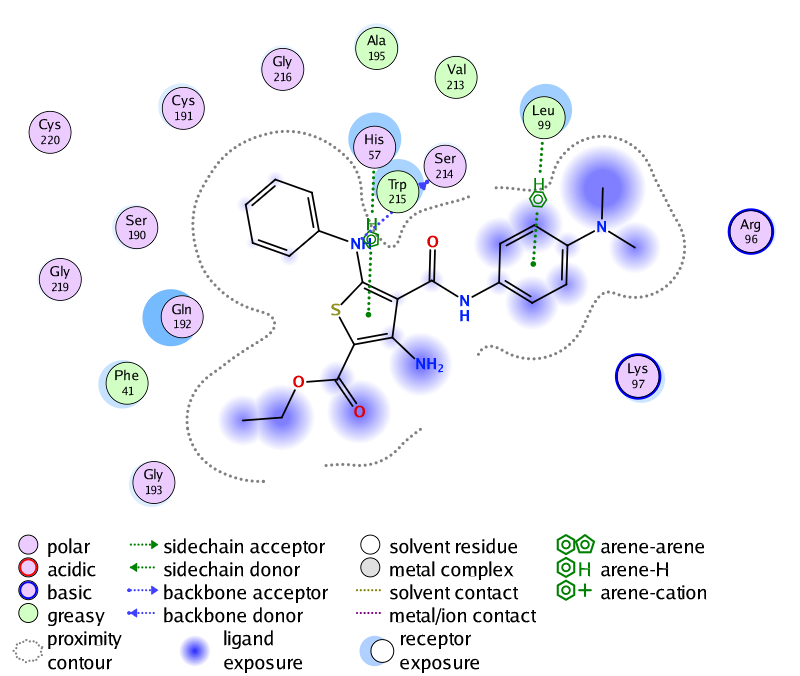 | 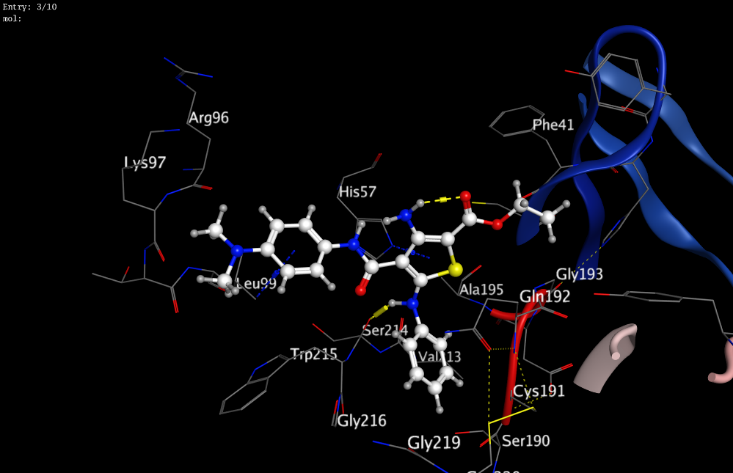 |
| --- | --- |
| **2D** | **3D** |
| 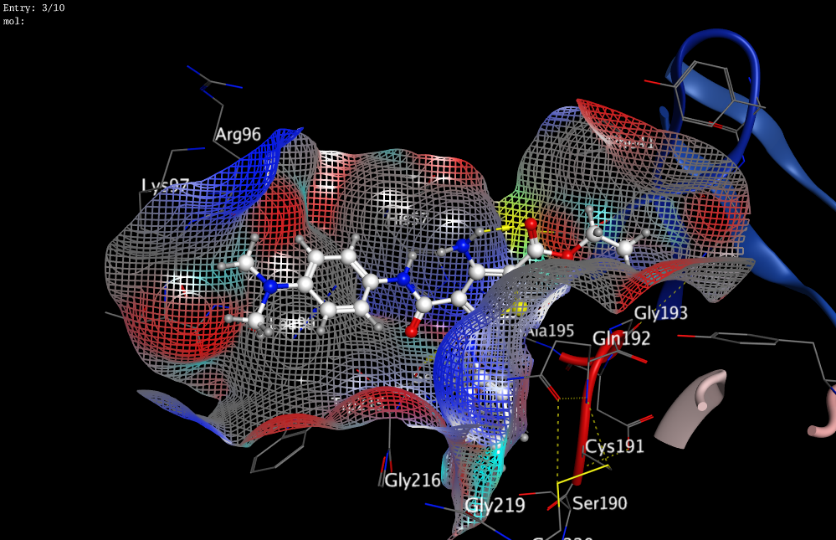 | |
| **Surface map** | |

**Figure S27:-** The binding interaction of **7**with (PDB ID: **2ra3**).

| 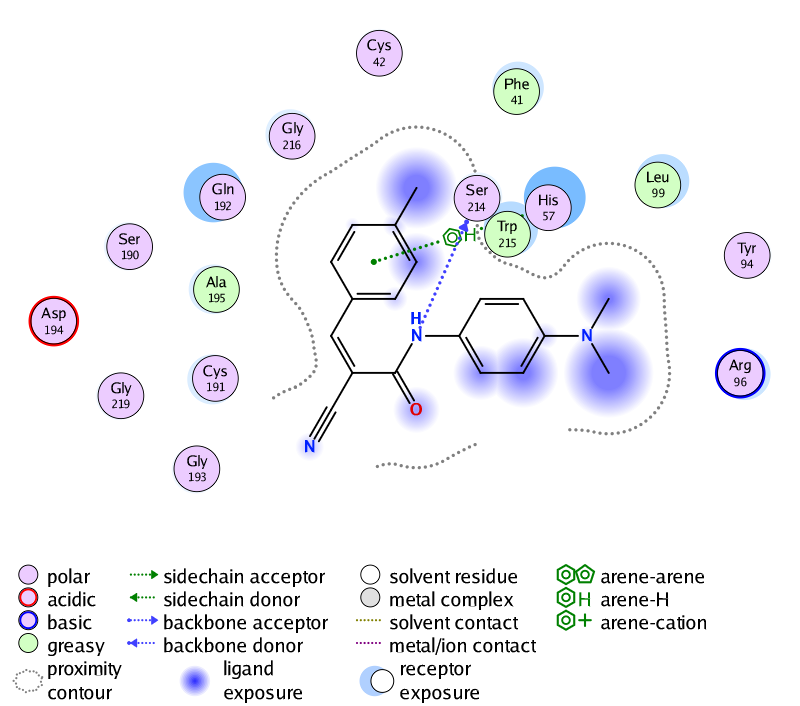 | 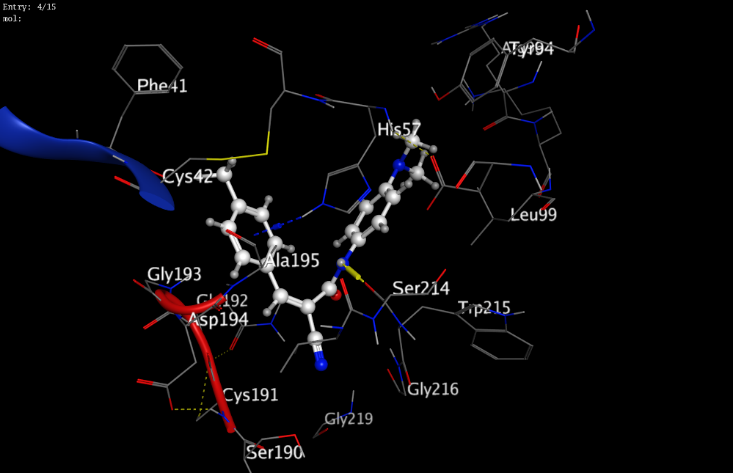 |
| --- | --- |
| **2D** | **3D** |
| 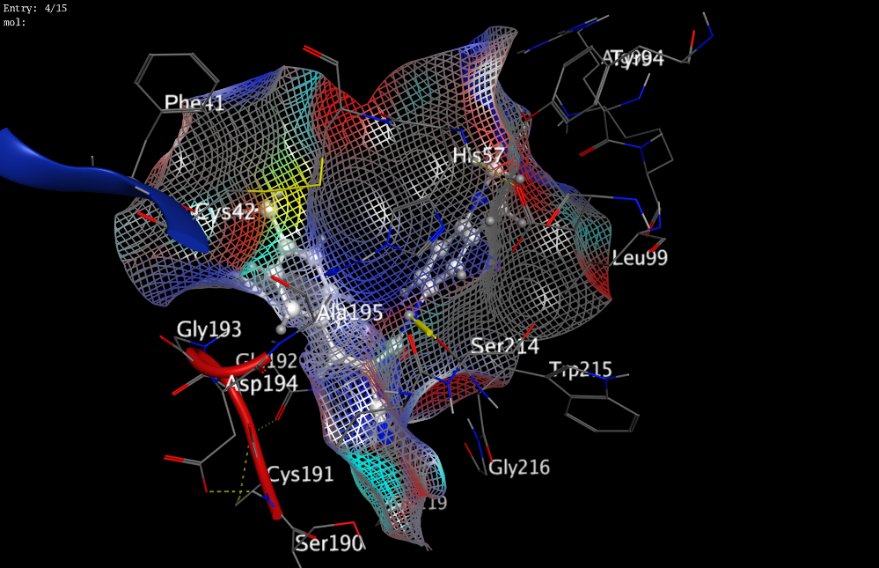 | |
| **Surface map** | |

**Figure S28:-** The binding interaction of **8a**with (PDB ID: **2ra3**).

| 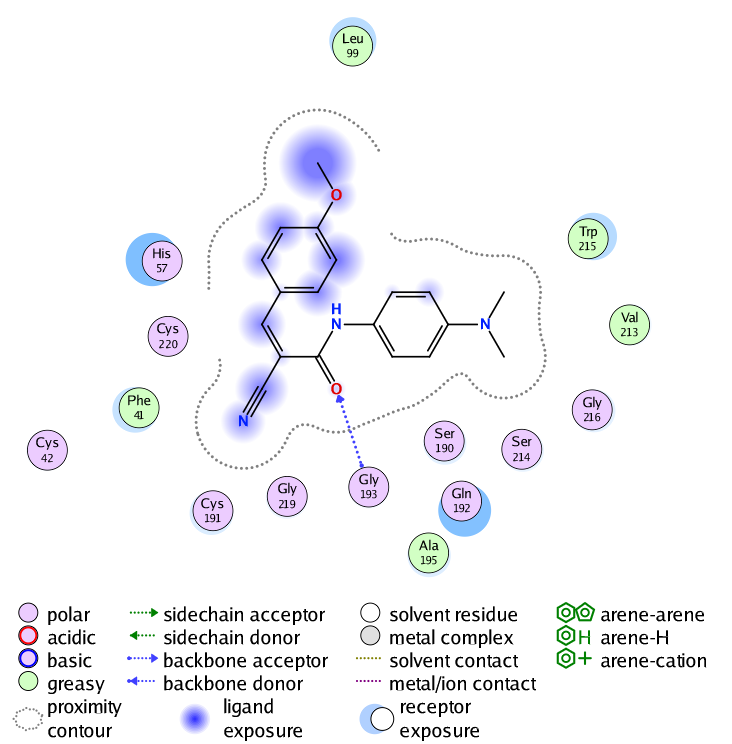 | 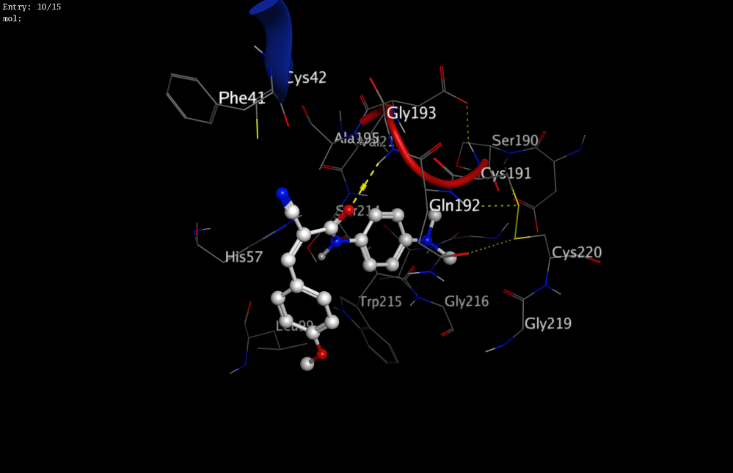 |
| --- | --- |
| **2D** | **3D** |
| 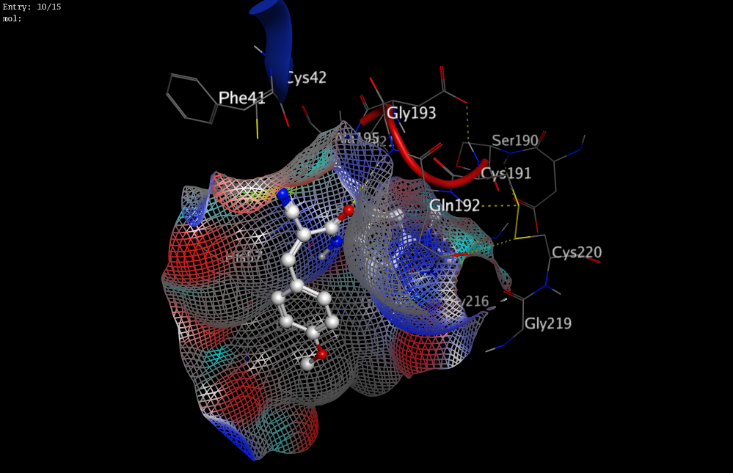 | |
| **Surface map** | |

**Figure S29:-** The binding interaction of **8b** with (PDB ID: **2ra3**).

| 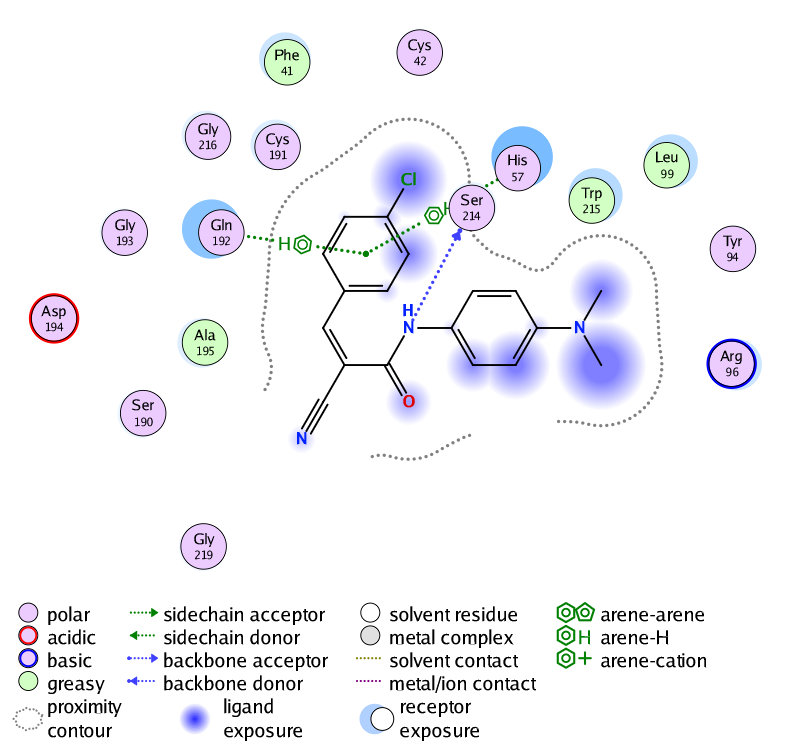 | 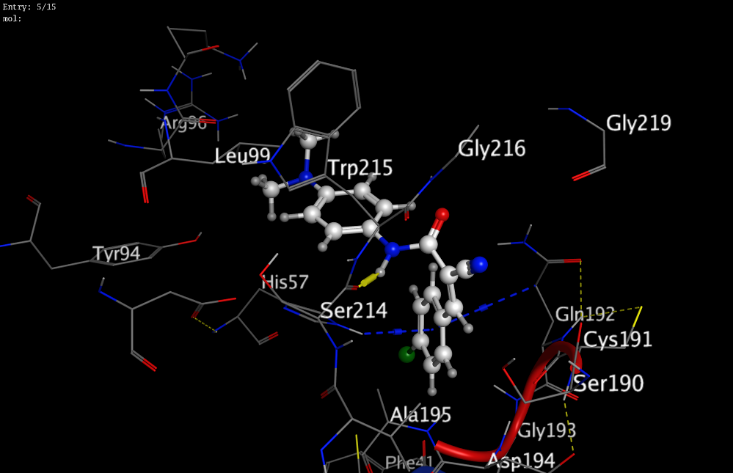 |
| --- | --- |
| **2D** | **3D** |
| 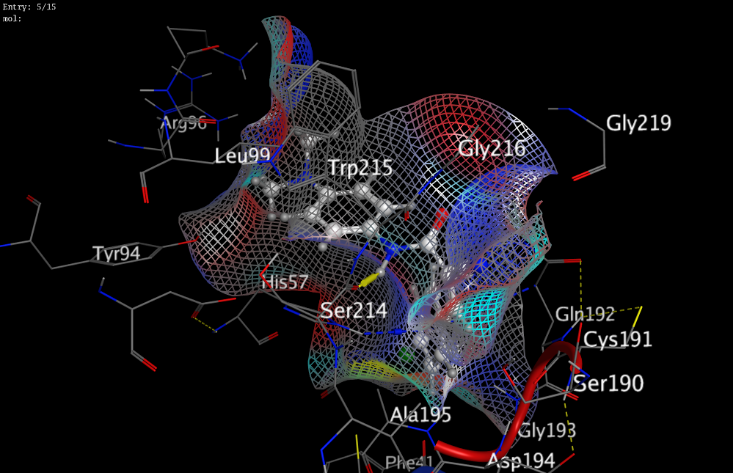 | |
| **Surface map** | |

**Figure S30:-** The binding interaction of **8c** with (PDB ID: **2ra3**).

| 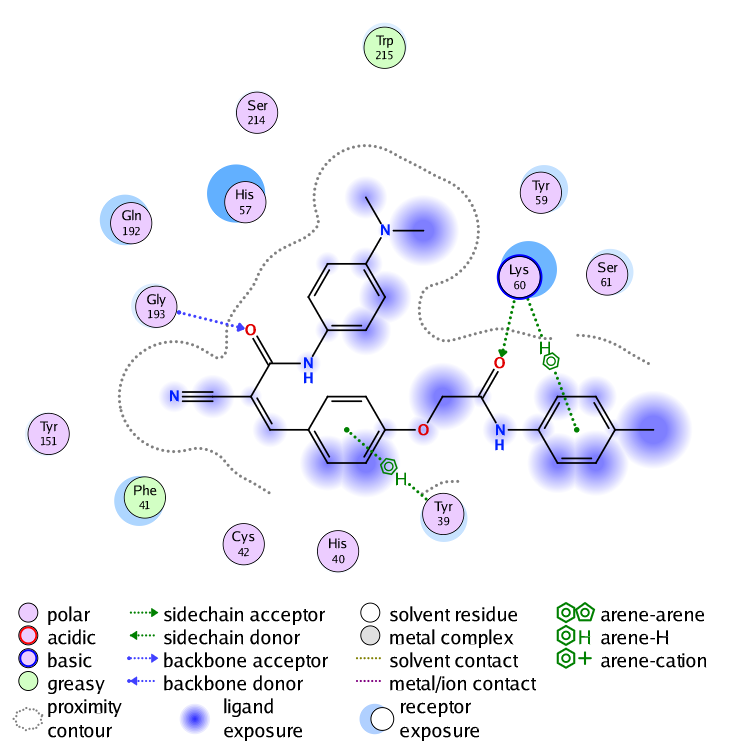 | 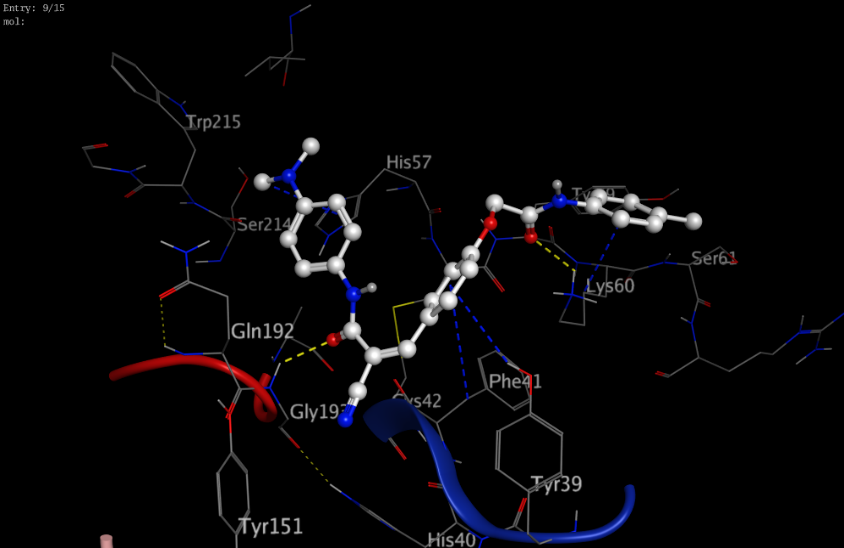 |
| --- | --- |
| **2D** | **3D** |
| 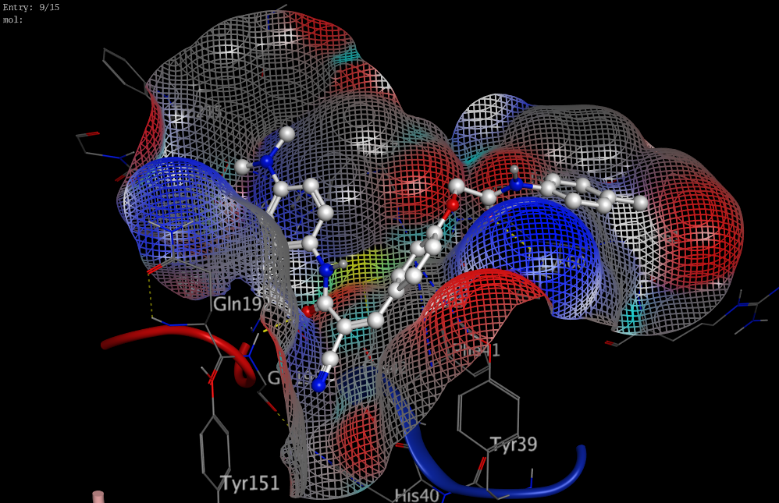 | |
| **Surface map** | |

**Figure S31:-** The binding interaction of **8d** with (PDB ID: **2ra3**).

| 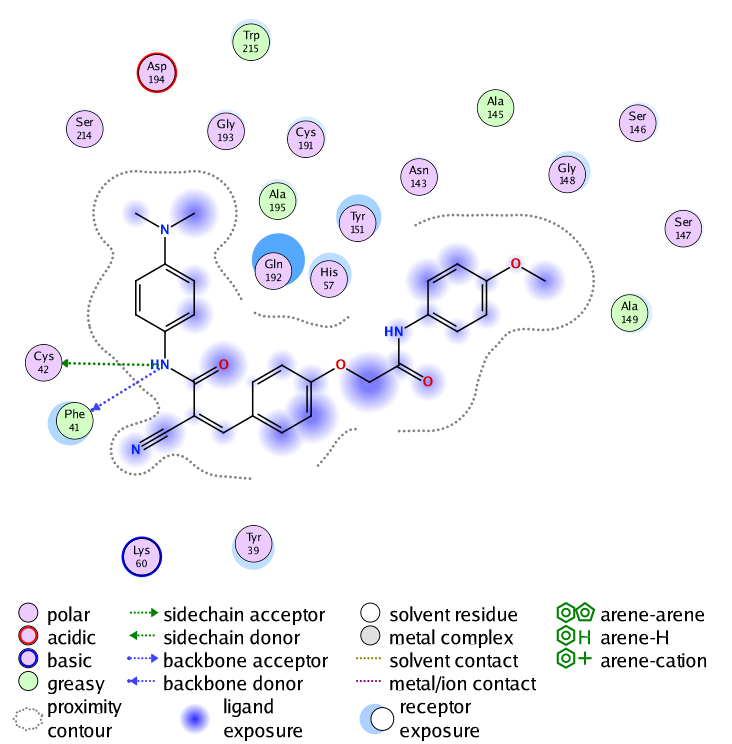 | 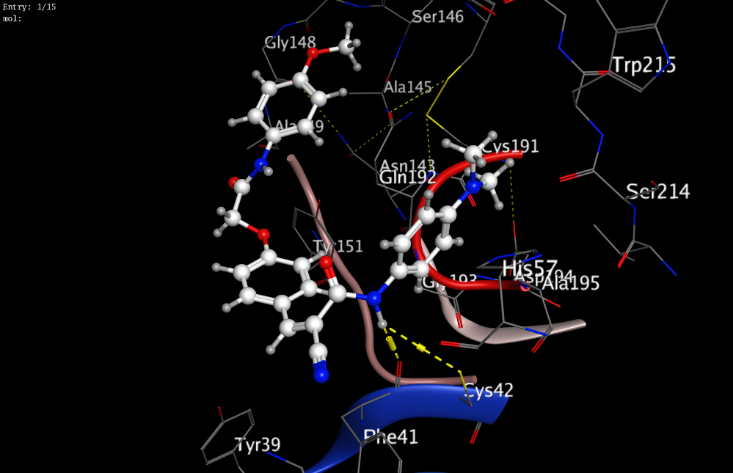 |
| --- | --- |
| **2D** | **3D** |
| 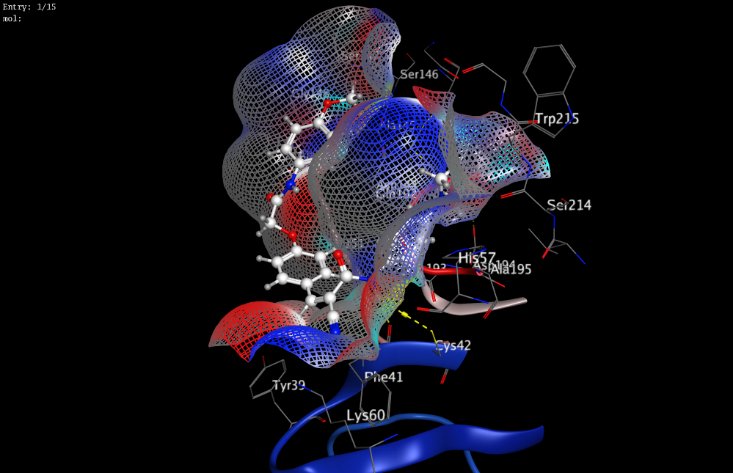 | |
| **Surface map** | |

**Figure S32:-** The binding interaction of **8e** with (PDB ID: **2ra3** ).

| 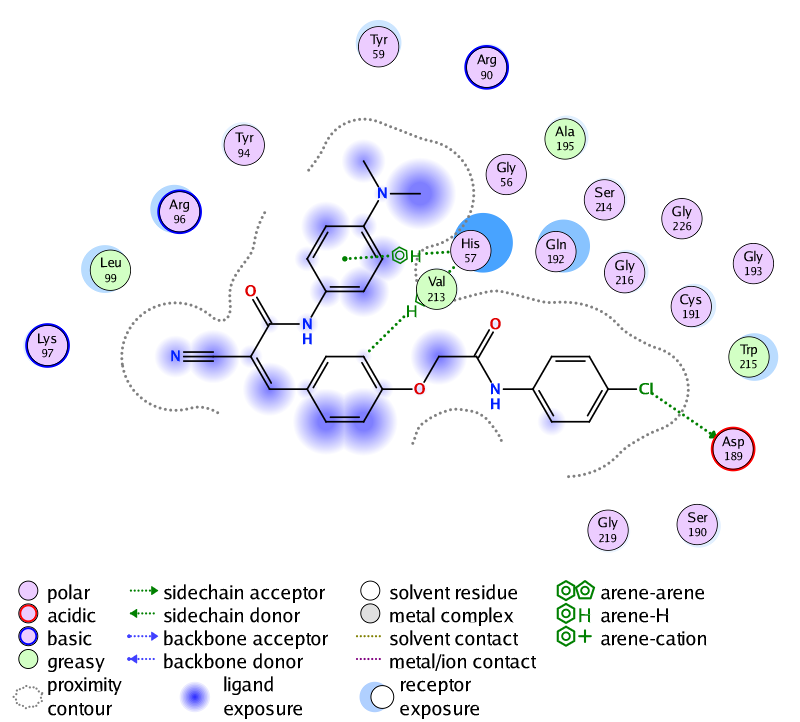 | 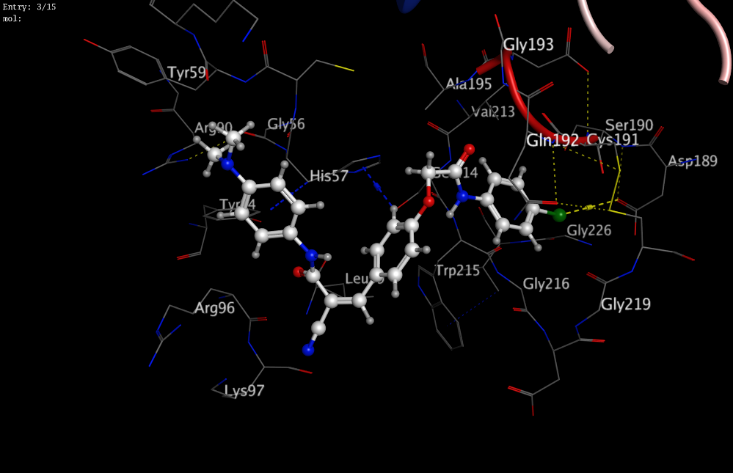 |
| --- | --- |
| **2D** | **3D** |
| 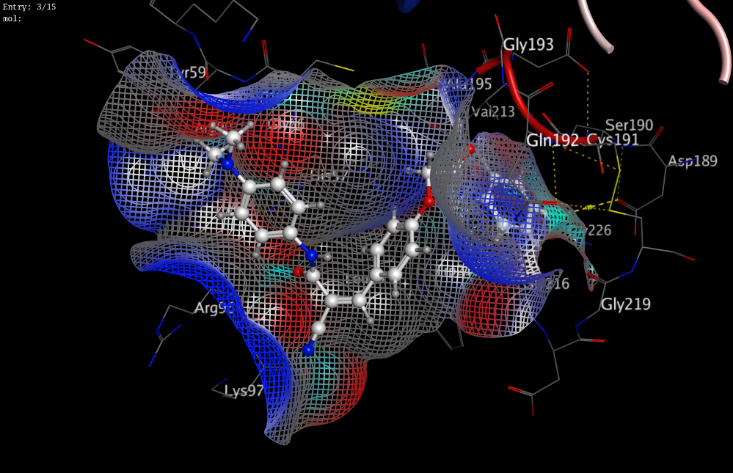 | |
| **Surface map** | |

**Figure S33:-** The binding interaction of **8f** with (PDB ID: **2ra3**).

| 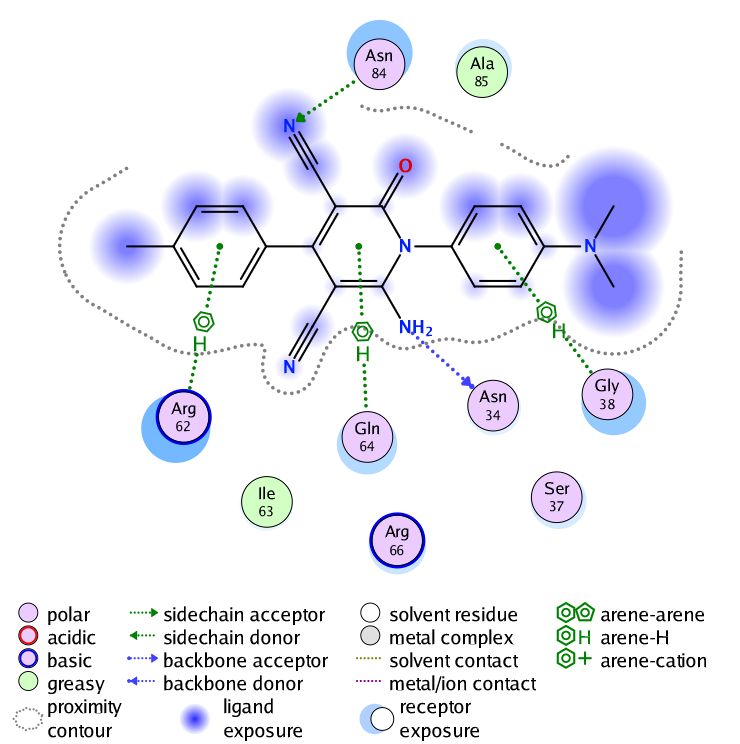 |  |
| --- | --- |
| **2D** | **3D** |
|  | |
| **Surface map** | |

**Figure S34:-** The binding interaction of **9a**with (PDB ID: **2ra3**).

|  |  |
| --- | --- |
| **2D** | **3D** |
|  | |
| **Surface map** | |

**Figure S35:-** The binding interaction of **9b** with (PDB ID: **2ra3**).

|  |  |
| --- | --- |
| **2D** | **3D** |
|  | |
| **Surface map** | |

**Figure S36:-** The binding interaction of **9c** with (PDB ID: **2ra3**).

|  |  |
| --- | --- |
| **2D** | **3D** |
|  | |
| **Surface map** | |

**Figure S37:-** The binding interaction of **9d** with (PDB ID: **2ra3**).

|  |  |
| --- | --- |
| **2D** | **3D** |
|  | |
| **Surface map** | |

**Figure S38:-** The binding interaction of **9e** with (PDB ID: **2ra3**).

|  |  |
| --- | --- |
| **2D** | **3D** |
|  | |
| **Surface map** | |

**Figure S39:-** The binding interaction of **9f** with (PDB ID: **2ra3**).

|  |  |
| --- | --- |
| **2D** | **3D** |
|  | |
| **Surface map** | |

**Figure S40:-** The binding interaction Doxorubicin with (PDB ID: **2ra3**).

| **Table S3** Interaction between drugs **1-9(a-f)** and target proteins (4y72 and 2ra3) and their docking scores. | | | | | | | | | | | | |
| --- | --- | --- | --- | --- | --- | --- | --- | --- | --- | --- | --- | --- |
|  | **(PDB ID: 4y72)** | | | | | | **(PDB ID: 2ra3)** | | | | | |
| **Cpd No** | **S (Energy binding score)**  **(Kcal/mol)** | **RMSD** | **Distance (A)** | **Binding Interaction** | | | **S (Energy binding score)**  **(Kcal/mol)** | **RMSD** | **Distance (A)** | **Binding Interaction** | | |
|  |  |  |  | **Ligand** | **Receptor** | **Type** |  |  |  | **ligand** | **Receptor** | **Type** |
| **1** | -5.2795 | 1.4068 | 3.30 | *N*-amide | His 252 | H-donor | -4.7415 | 1.2997 | 3.24 | *N*-Cyano | Trp 237 | H-acceptor |
| **2** | -6.9109 | 1.0970 | 3.25  3.53  3.06  3.17 | S-Thiazole  *N*-Amide  O-Amide  *N*-Cyano | Gln 132  Asp 146  Tyr 15  Lys 130 | H-donor  H-donor  H-acceptor  H-acceptor | -6.8192 | 0.8671 | 2.90  3.21  3.95  4.13 | O-Amide  O-Amide  Benzene ring  Thiazole ring | Gly 193  Ala 195  His 57  Trp 215 | H-acceptor  H-acceptor  π-H  π-H |
| **3** | -7.6510 | 1.1019 | 3.80 | Benzene ring | Ile 10 | π –H | -5.6054 | 0.9981 | 3.64  4.28 | *N*-Cyano  Benzene ring | Lys 87  Thr 241 | H-acceptor  π-H |
| **4** | -7.3130 | 1.5519 | 3.12  4.03  4.22 | N-dimethylamino  Benzene ring  Benzene ring | Tyr 15  Ile 10  Ile 10 | H-acceptor  π -H  π -H | -6.7160 | 0.8251 | 3.30 | *N*-Cyano | Cys 42 | H-donor |
| **6** | -7.4300 | 1.3082 | 3.08  3.27  3.47  4.13 | *N*-Amino  *N*-Amide  C-Benzene  Benzene ring | Asp 86  Ile 10  Asp 86  Val 18 | H-donor  H-donor  H-donor  π –H | -7.5094 | 1.3200 | 2.93  3.96 | *O*-Amide  Thiohene ring | Gly 193  Gln 192 | H-acceptor  π-H |
| **7** | -7.7892 | 1.2244 | 3.22  3.54  4.03 | *N*-Amino  C-Methylamine  Thiophene ring | Tyr 15  Phe 80  Glu 12 | H-acceptor  H-π  π –H | -6.9313 | 1.2279 | 2.84  3.68  4.15 | *N*-aniline  Thiophene ring  Benzene ring | Ser 214  His 57  Leu 99 | H-donor  π-H  π-H |
| **8a** | -6.5701 | 1.1832 | 2.91  4.07  4.53 | *N*-Amide  Benzene ring  Benzene ring | Ile 10  Val 18  Val 18 | H-donor  π –H  π –H | -6.1480 | 1.0473 | 2.97  3.87 | *N*-Amide  Benzene ring | Ser 214  His 57 | H-donor  π-H |
| **8b** | -7.2915 | 0.9063 | 3.62  3.90 | *N*-Nitrile  Benzene ring | Leu 83  Met 85 | H-acceptor  π –H | -5.9672 | 1.2666 | 3.49 | *O*-Amide | Gly 193 | H-acceptor |
| **8c** | -7.2328 | 1.1532 | 3.67  3.57  3.05  4.50  3.88 | Cl-atom  *N*-Nitrile  *N*-Dimethylamino  Benzene ring  Benzene ring | Glu 51  Leu 83  Tyr 15  Ala 145  Phe 80 | H-donor  H-acceptor  H-acceptor  π –H  π – π | -6.0349 | 0.9904 | 2.98  3.85  4.36 | *N*-Amide  Benzene ring  Benzene ring | Ser 214  His 57  Gln 192 | H-donor  π-H  π-H |
| **8e** | -7.8557 | 1.0221 | 2.98  4.48  4.28  4.09 | *N*-Amide  Benzene ring  Benzene ring  Benzene ring | Gln 132  Thr 14  Val 18  Lys 130 | H-donor  π -H  π -H  π -cation | -7.0988 | 1.3180 | 3.23  4.20 | *N*-Amide  *N*-Amide | Phe 41  Cys 42 | H-donor  H-donor |
| **9a** | -6.0175 | 0.6665 | 4.90 | Benzene ring | Tyr 270 | π -H | -5.8431 | 0.9731 | 3.12  3.06  3.07  4.00  4.12  4.05 | *N*-Amino  *N*-Amino  *N*-Nitrile  Benzene ring  Benzene ring  Benzene ring | Asn 34  Asn 34  Asn 84  Gly 38  Arg 62 Gln 64 | H-donor  H-donor  H-acceptor  π-H  π-H  π-H |
| **9b** | -6.8176 | 1.4412 | 3.50 | *N*-Amino | LEU 83 | H-donor | -5.4408 | 0.5528 | 3.52  3.15 | *N*-Nitrile  *N*-Nitrile | Ile 89  Arg 90 | H-acceptor  H-acceptor |
| **9c** | -6.7848 | 0.8277 | 3.07  3.65 | Cl-atom  Benzene ring | Glu 81  Tyr 15 | H-donor  π –H | -6.3615 | 0.8980 | 3.00  3.40  3.19  4.19 | *N*-Amino  *O*-Pyridone  *N*-Nitrile  Benzene ring | Ser 214  Gly 193  Gly 193  Ala 195 | H-donor  H-acceptor  H-acceptor  π –H |
| **9e** | -7.6026 | 1.1598 | 2.93 | *O-*Amide | Glu 12 | H-acceptor | -6.8987 1 | .1985 | 3.02  2.99 | *N*-Amide  *O-*Amide | Ala 86  Arg 62 | H-donor  H-acceptor |
| **Dox** | -8.8931 | 1.4144 | 2.72 | *O-*hydroxy | Asn 133 | H-donor | -6.2060 | 1.4534 | 3.00  3.67  4.53 | *N*-Amino  Benzene ring  Quninone ring | Arg 96  His 57  Gln 192 | H-donor  π –H  π –H |

**Anticancer Activity**

**Table S4** Cell viability and growth inhibition percent after treatment of cells with 25 µM of the tested compounds

|  | HepG2 | | MDA-MB-231 | |
| --- | --- | --- | --- | --- |
|  | Cell viability (%) | Growth Inhibition (%) | Cell viability (%) | Growth Inhibition (%) |
| 1 | 86 | 14 | 68 | 32 |
| 2 | 20 | 80 | 46 | 54 |
| 3 | 85 | 15 | 64 | 36 |
| 4 | 94 | 6 | 72 | 28 |
| 5 | 61 | 39 | 47 | 53 |
| 6 | 45 | 55 | 31 | 69 |
| 7 | 36 | 64 | 33 | 57 |
| 8a | 64 | 36 | 60 | 40 |
| 8b | 70 | 30 | 67 | 33 |
| 8c | 72 | 28 | 75 | 25 |
| 8d | 81 | 19 | 80 | 20 |
| 8e | 73 | 27 | 64 | 36 |
| 8f | 68 | 32 | 60 | 40 |
| 9a | 63 | 37 | 50 | 50 |
| 9b | 66 | 34 | 70 | 30 |
| 9c | 48 | 52 | 32 | 58 |
| 9d | 75 | 25 | 76 | 24 |
| 9e | 67 | 33 | 58 | 42 |
| 9f | 70 | 30 | 63 | 37 |

**Figure S41.** IC_50_% for the compound 2 against HepG2, MDA-MB-231 cell lines

**Figure S42.** Microscopic images of HepG2 cells following 48 h of exposure to compounds 2, 6, 7, and 9c with different concentrations (50, 25, and 12.5 µM)

**Figure S43.** Microscopic images of MDA-MB-231 cells following 48 h of exposure to exposure to compounds 2, 6, 7, and 9c with different concentrations (50, 25, and 12.5 µM)

**Figure S44.** ^1^H-NMR spectrum of compound **1.**

**Figure S45.** ^13^C-NMR spectrum of compound **1.**

**Figure S46.** ^1^H-NMR spectrum of compound **2.**

**Figure S47.** ^13^C-NMR spectrum of compound **2.**

**Figure S48.** ^1^H-NMR spectrum of compound **3.**

**Figure S49.** ^13^C-NMR spectrum of compound **3.**

**Figure S50.** ^1^H-NMR spectrum of compound **4.**

**Figure S51.** ^13^C-NMR spectrum of compound **4.**

**Figure S52.** ^1^H-NMR spectrum of compound **5.**

**Figure S53.** ^13^C-NMR spectrum of compound **5.**

**Figure S54.** ^1^H-NMR spectrum of compound **6.**

**Figure S55.** ^13^C-NMR spectrum of compound **6.**

**Figure S56.** ^1^H-NMR spectrum of compound **7.**

**Figure S57.** ^13^C-NMR spectrum of compound **7.**

**Figure S58.** ^1^H-NMR spectrum of compound **8a.**

**Figure S59.** ^13^C-NMR spectrum of compound **8a.**

**Figure S60.** ^1^H-NMR spectrum of compound **8b.**

**Figure S61.** ^13^C-NMR spectrum of compound **8b.**

**Figure S62.** ^1^H-NMR spectrum of compound **8c.**

**Figure S63.** ^13^C-NMR spectrum of compound **8c.**

**Figure S64.** ^1^H-NMR spectrum of compound **8d.**

**Figure S65.** ^13^C-NMR spectrum of compound **8d.**

**Figure S66.** ^1^H-NMR spectrum of compound **8e.**

**Figure S67.** ^13^C-NMR spectrum of compound **8e.**

**Figure S68.** ^1^H-NMR spectrum of compound **8f.**

**Figure S69.** ^13^C-NMR spectrum of compound **8f.**

**Figure S70.** ^1^H-NMR spectrum of compound **9a.**

**Figure S71.** ^1^H-NMR spectrum of compound **9b.**

**Figure S72.** ^1^H-NMR spectrum of compound **9c.**

**Figure S73.** ^1^H-NMR spectrum of compound **9d.**

**Figure S74.** ^1^H-NMR spectrum of compound **9e.**

**Figure S75.** ^1^H-NMR spectrum of compound **9f.**
